# Arctic introgression and chromatin regulation facilitated rapid Qinghai-Tibet Plateau colonization by an avian predator

**DOI:** 10.1038/s41467-022-34138-3

**Published:** 2022-10-27

**Authors:** Li Hu, Juan Long, Yi Lin, Zhongru Gu, Han Su, Xuemin Dong, Zhenzhen Lin, Qian Xiao, Nyambayar Batbayar, Batbayar Bold, Lucia Deutschová, Sergey Ganusevich, Vasiliy Sokolov, Aleksandr Sokolov, Hardip R. Patel, Paul D. Waters, Jennifer Ann Marshall Graves, Andrew Dixon, Shengkai Pan, Xiangjiang Zhan

**Affiliations:** 1 Key Laboratory of Animal Ecology and Conservation Biology, Institute of Zoology, Chinese Academy of Sciences, 100101 Beijing, China; 2 Cardiff University - Institute of Zoology Joint Laboratory for Biocomplexity Research, Chinese Academy of Sciences, 100101 Beijing, China; 3 University of the Chinese Academy of Sciences, 100049 Beijing, China; 4 Ministry of Education Key Laboratory for Biodiversity Science and Ecological Engineering, College of Life Sciences, Beijing Normal University, 100875 Beijing, China; 5 Wildlife Science and Conservation Center, Union Building B-802, Ulaanbaatar, 14210 Mongolia; 6 Raptor Protection of Slovakia, Trhová 54, SK-841 01, Bratislava, Slovakia; 7 Wild Animal Rescue Centre, Krasnostudencheskiy pr., 21-45, Moscow, 125422 Russia; 8 Institute of Plant and Animal Ecology, Ural Division Russian Academy of Sciences, 202-8 Marta Street, Ekaterinburg, 620144 Russia; 9 Arctic Research Station of the Institute of Plant and Animal Ecology, Ural Division Russian Academy of Sciences, 21 Zelenaya Gorka, Labytnangi, Yamalo-Nenetski District 629400 Russia; 10 The John Curtin School of Medical Research, Australian National University, Canberra, ACT 2601 Australia; 11 School of Biotechnology and Biomolecular Science, Faculty of Science, UNSW Sydney, Sydney, NSW 2052 Australia; 12 School of Life Sciences, La Trobe University, Melbourne, Australia; 13 Emirates Falconers’ Club, Al Mamoura Building (A), P.O. Box 47716, Muroor Road, Abu Dhabi, UAE; 14 International Wildlife Consultants, P.O. Box 19, Carmarthen, SA33 5YL UK; 15 Center for Excellence in Animal Evolution and Genetics, Chinese Academy of Sciences, Kunming, 650223 China

**Keywords:** Evolutionary genetics, Gene regulation, Genome evolution, Genetic hybridization

## Abstract

The Qinghai-Tibet Plateau (QTP), possesses a climate as cold as that of the Arctic, and also presents uniquely low oxygen concentrations and intense ultraviolet (UV) radiation. QTP animals have adapted to these extreme conditions, but whether they obtained genetic variations from the Arctic during cold adaptation, and how genomic mutations in non-coding regions regulate gene expression under hypoxia and intense UV environment, remain largely unknown. Here, we assemble a high-quality saker falcon genome and resequence populations across Eurasia. We identify female-biased hybridization with Arctic gyrfalcons in the last glacial maximum, that endowed eastern sakers with alleles conveying larger body size and changes in fat metabolism, predisposing their QTP cold adaptation. We discover that QTP hypoxia and UV adaptations mainly involve independent changes in non-coding genomic variants. Our study highlights key roles of gene flow from Arctic relatives during QTP hypothermia adaptation, and *cis*-regulatory elements during hypoxic response and UV protection.

## Abstract (web summary)

The Qinghai-Tibet Plateau is as cold as the Arctic, but presents unique hypoxia and high ultraviolet conditions. Here the authors find that gene flow from Arctic gyrfalcons aids plateau saker falcons’ cold adaptation, and independent non-coding genomic changes underlie hypoxic and ultraviolet responses.

## Introduction

The Qinghai-Tibet Plateau, also known as the Third Pole, has a climate as cold as the Arctic. To cope with cold extremes, animals living in these two poles have evolved similar morphologies such as large body size, and long, thick wintering fur^1^. Previous fossil analyses even suggested that cold adaptive traits of some Arctic mammals (e.g. Arctic fox and woolly rhinoceros) may have occurred already in their QTP ancestors^1,2^. Recent phylogenetic studies have found that QTP and Arctic animals are closely related^3,4^, but to date it is unclear whether there was gene flow between the two poles that facilitated cold adaptation of QTP animals, or whether these adaptations were gained independently.

Different from Arctic relatives, QTP animals have also evolved unusual morphological and physiological traits (e.g. increased hemoglobin-oxygen affinity, protective pigmentation) to cope with stresses of low oxygen and UV radiation experienced at high altitude^5,6^. Accumulating evidence has demonstrated a key role of natural selection in the evolution of these unique QTP traits. Previous studies have attributed a few coding variations to these QTP phenotypes^7^, but it was recently found that the majority of selective loci occurred in non-coding regions^8^, implying that gene regulation elements may have an important role in QTP hypoxic adaptation and response. Among them, *cis*-regulatory elements are a group of well recognized non-coding DNAs, and variations on them, especially promoter modifications, were found to alter gene transcription^9,10^. However, promoter variations generally account only for a small proportion (about 1%)^8^ of non-coding selective loci, how the selected variants on non-coding regions regulate gene expression related to QTP adaptation or response remains largely unknown.

Saker falcons (*Falco cherrug*), provide an ideal model to address these issues because sakers have a broad breeding distribution across Eurasia^11^ and a recent colonization to the QTP^12^. The origin and evolution of saker populations have been explored using limited sequence information. Comparison of the 460 bp mitochondrial control region sequences suggested a very recent and central European origin of this species^13,14^, but revealed no genetic partitions across Eurasian populations. Our pilot cDNA-based study covering about 4% of genomic regions indicated that sakers may have initially inhabited central Europe, dispersed to central Asia, and finally colonized to the QTP^12^. However, our current understanding of saker colonization processes may be compromised by the non-neutral nature of cDNA variants^15^. Moreover, studies of mitochondrial DNA have produced some evidence of gene flow between sakers and gyrfalcons (*Falco rusticolus*), the largest falcon species, which is distributed mainly in the Arctic and subarctic tundra^16^. The evidence for this is debated, however, as the mixed mitochondrial ancestry^13,14^ may also result from a recent divergence^17^.

**Fig. 1:**
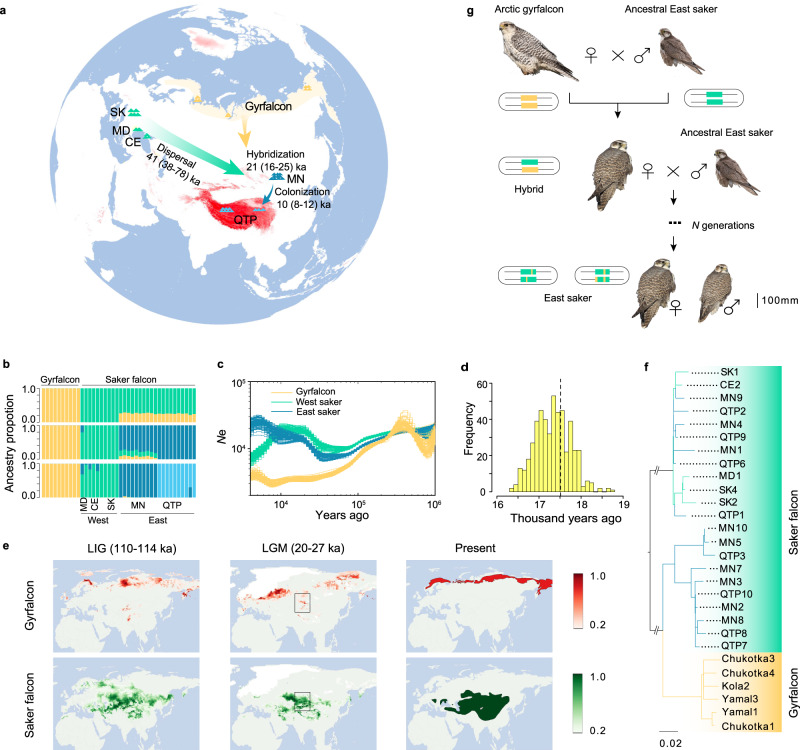
Stepwise colonization of saker falcons onto the Qinghai-Tibet Plateau. **a** Sampling localities for sakers and gyrfalcons and the colonization route of sakers. SK Slovakia, MD Moldova, CE Crimea, MN Mongolia, QTP Qinghai-Tibet Plateau. **b** Population structure of falcons (*K* = 2-4). **c** Demographic histories for both species (*μ* = 1.1E–08, *g* = 6.6 years). **d** Introgression time estimation (500 bootstraps). The dashed line shows the estimated maximum likelihood time. **e** Simulation of potential breeding areas with suitable climate for sakers and gyrfalcons in the last interglacial (LIG) and last glacial maximum (LGM). The rectangles show overlapped breeding areas between gyrfalcons and sakers in the LGM. The bars represent suitability scores. The present breeding areas for gyrfalcons and sakers were derived from previous studies^11,17^. **f** Phylogenetic tree reconstructed using the variants of W chromosome. **g** Illustration showing maternally biased hybridization (female gyrfalcon × male saker). The bar scales body length. The gyrfalcon photo is taken by Aleksandr Sokolov, and the saker photo used as the ancestral East saker is taken by Jozef Chavko. Other saker photos are taken by our lab. Source data are provided as a Source Data file.

Here, we *de novo* assemble a chromosome-level saker genome as reference and perform whole genome resequencing of 30 sakers across their main breeding distribution:^11^ western Eurasia (West) comprising Moldova (MD), Slovakia (SK) and Crimea (CE), eastern Eurasia (East) including Mongolia (MN) and QTP (Fig. 1a). We have also sequenced 10 gyrfalcons sampled across northern Russia (Eurasian Arctic, Fig. 1a). With these population genomic data, we verify the occurrence of introgression from gyrfalcons to sakers during the last glacial maximum (LGM), and elucidate its indispensable role in colonization of the low temperature QTP by sakers. Combined with the comparative 3D genome and functional genomics analysis, we highlight a unique contribution of mutations on *cis*-regulatory elements to plateau hypoxic response (enhanced chromatin interaction) and UV protection (elevated melanin synthesis).

## Results

### Stepwise colonization of sakers onto the QTP

Although sakers have a wide distribution across Eurasia, and their prominent roles in grassland ecosystem balance and human culture have been well recognized for a long time^14,18^, we still know little about their detailed evolutionary history. To clarify the history of saker colonization, we assembled a complete saker genome and resequenced the whole genomes of sakers from different geographic populations, as well as gyrfalcons from the Eurasian Arctic (Fig. 1a). We sequenced a female saker and assembled a 1.23 Gb genome by integrating PacBio, HiSeq and Bionano data (scaffold N50, 36.05 Mb), and anchored 1.20 Gb sequences to 38 super-scaffolds using Hi-C (Supplementary Figs. 1, 2; Supplementary Tables 1–5). Finally, we identified 24 autosomes (10 macro- and 14 micro- chromosomes) and ZW chromosomes by aligning the super-scaffolds against four bird genomes^19^ (“Methods”; Supplementary Fig. 3). We then generated an average of 26.38 Gb sequences (21×; Supplementary Data 1) for each of the 40 studied falcons using Illumina short read sequencing technology.

Both population genetic structure (*K* = 2; Fig. 1b; Supplementary Fig. 4) and principal component analysis (PCA; Supplementary Fig. 4) separated the studied falcon individuals into two clusters, gyrfalcons and sakers, with the latter further splitting into West and East populations (*K* = 3; Fig. 1b). We then used *SMC++*^20^ to reconstruct the demographic histories, and inferred that sakers and gyrfalcons had begun to diverge about 300 thousand years ago (ka) due to the observation of differentiation in effective population size (*N*e) (Fig. 1c), and supporting evidence comes from the fossil record^21^ and previous mitochondrial variations^22^.

Previous reports suggested a hybridization event between sakers and gyrfalcons after their divergence^13,14,22^, but could not pinpoint how, when, and where hybridization occurred. Our analysis of saker and gyrfalcon population genomic data showed that East sakers share more than 21.3% autosomal and 25.4% *Z* chromosomal alleles with gyrfalcons whereas West sakers share less than 0.2% and 0.0001% (*K* = 2; Fig. 1b; Supplementary Fig. 5), respectively, suggesting an asymmetric gene flow from gyrfalcons to East sakers. We also confirmed this using an *f*_3_-statistic method^23^and a significantly negative *f*_3_ (East saker; gyrfalcon, West saker) with a mean *Z*-score of -23.96 (Supplementary Table 6), strongly supported admixture between gyrfalcons and East sakers.

We next wanted to know when and where the hybridization had occurred. Using *Ancestry_HMM*^24^ to trace the ancestry of discrete genomic segments, we estimated the introgression time to be 17.5 ka (confidence interval, 16–19 ka) (Fig. 1d; “Methods”), falling into the LGM period (16–27 ka)^25^. Using Ecological Niche Modeling (ENM)^26^, we reconstructed potential breeding areas that had suitable climate for sakers and gyrfalcons in Eurasia during the last interglacial (LIG) and LGM, respectively (Fig. 1e). During the LGM, we found the breeding areas of gyrfalcons had shifted to southern Siberia, which overlapped with 370,488 km² of those predicted for sakers. We therefore propose that the introgression occurred in southern Siberia during the LGM. Supporting evidence comes from the discovery in the Altai Mountains of falcon fossils dated 25-45 ka, with the mixed characteristics of both species^27^. Our *MSMC*^28^ simulation implied that gene flow between the two species ceased *ca*. 10 ka (Supplementary Fig. 6). This may have been due to the northwards retreat of glaciers and geographical isolation between the two species as a result of the formation of the Siberian boreal forest (*ca*. 12 ka)^29^.

We then investigated whether hybridization between the two species was sexually biased. We estimated the contribution of female gyrfalcons to the gene pool in ancient East sakers by analyzing maternally inherited SNPs from falcons’ W chromosomes. We found that West sakers and gyrfalcons were clearly separated into two clades (Fig. 1f), again confirming distinct genetic differentiation between West sakers and gyrfalcons. In contrast, the East sakers occurred in both clades with 58.8% (10/17) of the examined female sakers in the gyrfalcon clade, and 41.2% (7/17) in the West saker clade (Fig. 1f). This result indicated that more female sakers in East populations possessed genetic backgrounds from gyrfalcons. Because W chromosomes do not recombine (except for pseudoautosomal regions (PARs)), this proportion (58.8%) could proxy the contribution of female gyrfalcons to the ancient gene pool of female East sakers until hybridization ceased. Assuming that the ancestral saker population had the same sex ratio (1: 1) as present^30^, we estimated that female gyrfalcons contributed about 29.4% of the gene pool of the East saker population.

To estimate the contribution of male gyrfalcons to the East saker gene pool, we assumed the loss of ancient introgressed alleles at a constant rate and designed a method based on the observed introgression rates on autosomes and *Z* chromosome (“Methods”). We estimated that male gyrfalcons contributed only 13.6%, half of that from female gyrfalcons. This biased hybridization between female gyrfalcons and male sakers might reflect a size advantage for gyrfalcons in female competition for access to males and/or breeding territories^31^ (Fig. 1g).

The identification of the hybridization event between gyrfalcons and East sakers enabled us to reconstruct a map of saker colonization onto the QTP. Based on the detected population structure, population histories, and the identified introgression time with gyrfalcons, we used *fastsimcoal2*^32^ (Supplementary Table 7) to simulate population divergence and reconstructed a stepwise colonization route of QTP sakers: (1) *ca*. 41 ka (38-78 ka), sakers gradually dispersed from central Europe to East Asia (e.g. MN); (2) during the LGM, gyrfalcons shifted to southern Siberia from the Arctic and gene flow occurred between gyrfalcons and eastern Eurasia sakers at *ca*. 21 ka (16–25 ka); (3) *ca*. 10 ka (8–12 ka), sakers colonized the QTP, probably from the MN population (Fig. 1a; Supplementary Fig. 7). The elucidation of these stepwise processes provides an opportunity to systematically study unique genetic underpinnings that helped sakers colonize the QTP.

### Hybridization with Arctic gyrfalcons facilitated sakers’ adaptation to cold extremes

Paleoclimatic data^33^ analysis demonstrated that from *ca*. 50 ka to the present, East Asia (e.g. MN) had a lower annual mean temperature than central Europe. The lowest temperature in the coldest month (−30 °C) occurred during the LGM (Supplementary Fig. 8), coinciding with the time when gyrfalcons hybridized with ancestral East sakers. To live in cold Arctic conditions, gyrfalcons evolved adaptive traits such as the largest body size in extant Falconidae^16^. In line with recent work suggesting that genetic introgression has played an important role during the colonization of recipient species to new environments^34^, we hypothesize that gene flow from gyrfalcons to East sakers during the LGM promotes low temperature adaptation in East sakers.

**Fig. 2:**
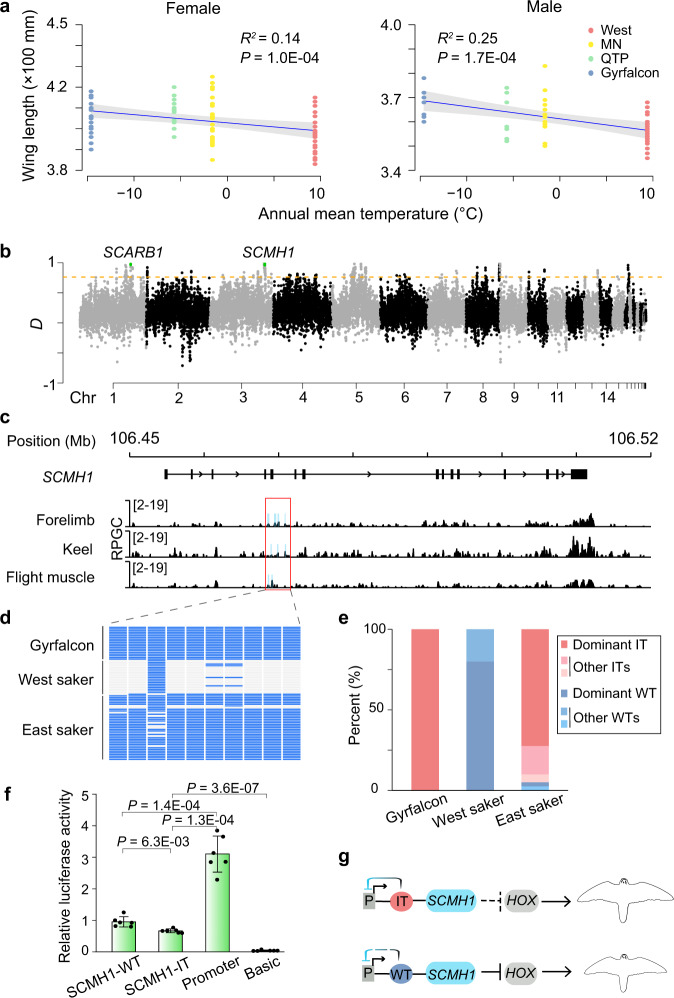
Larger body size in East sakers due to hybridization with Arctic gyrfalcons. **a** Correlation between wing length and annual mean temperature for both female (left; 27 gyrfalcons, 50 sakers including 19 from West, 20 from MN and 11 from QTP) and male falcons (right; nine gyrfalcons, 43 sakers including 17 from West, 18 from MN and eight from QTP) using a linear regression model. The mean (blue line) and 95% CI (gray band) are shown. Significance level was calculated using *F* test. **b** Distribution of *D* values along chromosomes. The orange line is the threshold of top 1% value (*D* = 0.73). **c** ATAC-seq tracks (normalized using reads per genome coverage (RPGC)) around the *SCMH1* gene. The sky-blue blocks show the identified peaks in each sample. The red box shows the *cis*-regulatory element (CRE) used for the luciferase reporter assay. The window size is 200 bp. **d** Haplotypes of the focal CRE in falcons. **e** Haplotype frequencies of the focal CRE in gyrfalcons, West and East sakers. IT introgressed type, WT wild type. **f** Relative luciferase activity comparison between dominant IT- and dominant WT- CREs in duck embryonic fibroblast cells. The SCMH1-IT and SCMH1-WT groups were cloned into pGL3-Promoter vectors. Promoter (pGL3-Promoter) and Basic (pGL3-Basic) groups were used as controls respectively. The bars display mean ± SD (*N* = 6 technical replicates). Three biologically independent replicates of luciferase experiment are shown in Supplementary Fig. 14. A two-sided *t* test was used. **g** A working model showing how the focal CRE haplotype affects the development of body size. “P” means promoter. Source data are provided as a Source Data file.

To test this hypothesis, we compared the body size (wing length as the indicator^35^) among East sakers (MN and QTP), West sakers and gyrfalcons. We found that sex-matched adult East sakers have a larger body size than West sakers (Fig. 2a; Supplementary Fig. 9). Our results, thus, concord with the Bergmann’s rule, that within a species, individuals living in a colder environment generally have a larger body size^36^.

We then wanted to know whether introgression from Arctic-adapted gyrfalcons contributed to the size differentiation between the two main saker populations. To do this, we applied an ABBA-BABA model^37^, and found five outstanding genomic islands (> 200 Kb) on the five chromosomes (Chr 1, 3, 5, 8 and 16) exhibiting adaptive introgression signatures (top 1% *D* = 0.73, top 1% *f*_d_ = 0.70) (Fig. 2b; Supplementary Fig. 10) which were much longer than the expected length of fragments (26.0 Kb) from incomplete lineage sorting (ILS; “Methods”). The strongest introgression signature came from the *sex comb on midleg homolog 1* (*SCMH1*) gene (*D* = 0.97, *f*_d_ = 0.89; Fig. 2b; Supplementary Figs. 10, 11) with all the variants located in non-coding regions (Supplementary Fig. 12). This gene appears to be associated with skeletal growth. *SCMH1*^*–/–*^ mutated mice were reported to have skeletal abnormalities^38^, and mutations on *SCMH1* were correlated with adult heights in European humans^39^ and body sizes in horses^40^.

We therefore investigated the roles of introgressed *SCMH1* variants in the development of body size in sakers. We detected transposase-accessible chromatin around the *SCMH1* gene using the ATAC-seq data from forelimb, keel and flight muscle of a saker embryo sample (Supplementary Table 8). We found a peak that spanned 3.3 Kb in the Intron 5 and covered 10 introgressed SNPs, which provided experimental support for its existence as a *cis*-regulatory element (CRE) (Fig. 2c; Supplementary Figs. 12c, 13). With the Hi-C data generated for sakers (Supplementary Table 2), we further found this element is co-located in the same topologically associating domain (TAD) with the *SCMH1* promoter (Supplementary Fig. 12), a fundamental chromatin topology. Contacts between CREs and promoters are mainly constrained within TADs^41^, so our findings imply that the active element could regulate the expression of *SCMH1*.

We phased this fragment using *BEAGLE*^42^ (Fig. 2d) and performed a functional study of the *SCMH1 cis*-regulatory element by comparing activities of dominant wild and dominant introgressed haplotypes (Fig. 2e) with a luciferase reporter assay expressed in duck embryonic fibroblast cells (CCL-141, ATCC). Our experiments showed that both haplotypes had suppressing functions, but the introgressed one had a stronger effect (*P* = 6.3E-03; Fig. 2f; Supplementary Fig. 14). Since SCMH1 acts as an E3 ubiquitin ligase to suppress the expression of growth-promoting *HOX* genes in mammals^43^, we suggest that the greater repressive effect of the *cis*-regulatory element on *SCMH1* in East sakers may relieve the suppression of *HOX* expression, which could lead to larger body size in East sakers (Fig. 2g). Across the genome, we also identified six other adaptively introgressed genes previously reported to be involved in animal body size development (Supplementary Table 9; Supplementary Data 2). These genes, together with *SCMH1*, comprised three gene blocks (*SCMH1/FOXO6*, *HMGA2/MSRB3/LEMD3*, and *FBXL15/NFKB2*), and our Hi-C analysis showed that each block was located in a different TAD (Supplementary Figs. 12, 15, 16). Collectively, our results suggest that hybridization with the Arctic gyrfalcons, the largest falcon species, provides new gene variants that promote larger body size and relieve hypothermia stress in the East saker population.

**Fig. 3:**
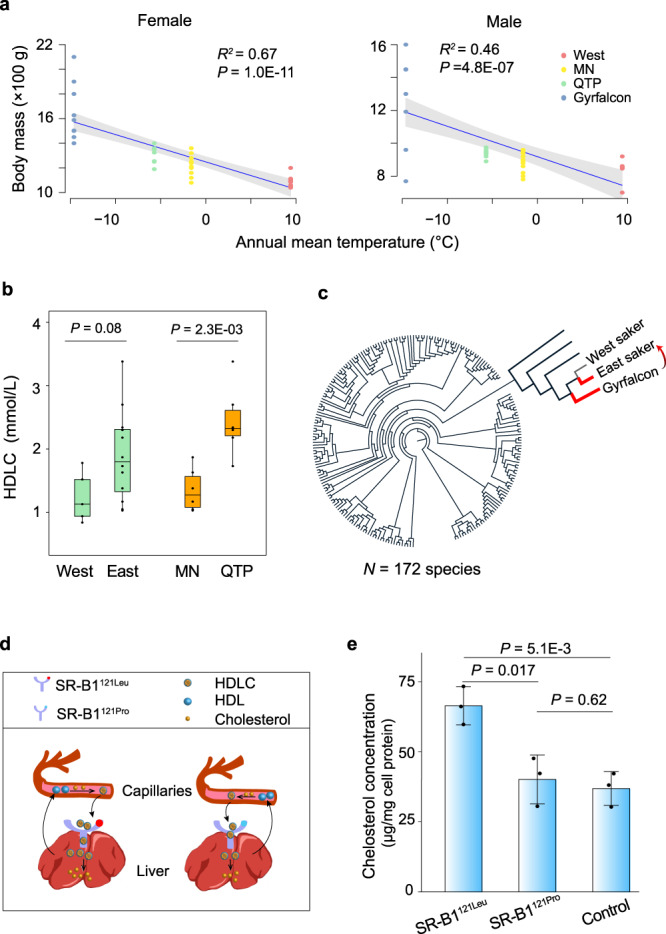
Higher body mass in East sakers due to introgressed SR-B1^121Leu^ from gyrfalcons. **a** Correlation between body mass and annual mean temperature for both female (left; 10 gyrfalcons, 34 sakers including 11 from West, 15 from MN and eight from QTP) and male (right; nine gyrfalcons, 34 sakers including eight from West, 16 from MN and ten from QTP) falcons using a linear regression model. The mean (blue line) and 95% CI (gray band) are shown. Significance level was calculated using *F* test. **b** High-density lipoprotein cholesterol (HDLC) concentration of plasma in comparisons of West (*N* = 6 biologically independent samples) and East sakers (*N* = 12 biologically independent samples), MN (*N* = 6 biologically independent samples) and QTP sakers (*N* = 6 biologically independent samples). A two-sided *t* test was used. In the box plots, the center line represents the median, whiskers represent maximum and minimum values, and box boundaries represent 75th and 25th percentiles. **c** Amino acid on SR-B1^121^ in 172 avian species; black and red branches represent species featuring SR-B1^121Pro^ and SR-B1^121Leu^, respectively. **d** Scheme showing how SR-B1^121Pro^ promotes HDLC uptake from blood to liver. HDL high-density lipoprotein. **e** Cellular cholesterol concentrations in HeLa cells transfected with SR-B1^121Leu^, SR-B1^121Pro^ and control plasmids, respectively. The bars display mean ± SD (*N* = 3 independent experiments). A two-sided *t* test was used. Source data are provided as a Source Data file.

Polar animals such as polar bears and penguins feature higher body mass and fat storage relative to temperate animals as classical adaptations to meet high energy demands under the extremely cold polar environments^44,45^. To test whether this is the case for East sakers, we compared the body mass of East (MN and QTP) with West sakers, and found that the former was significantly heavier in both sexes (Fig. 3a; Supplementary Fig. 9), in line with observations that birds living in the cold regions are heavier^46^. However, a larger body size may also cause a higher body mass. To control this effect, we checked the body mass index (BMI, body mass/wing length^47^) of Mongolian sakers^48^ and obtained a BMI coefficient for adult males and females, respectively (Supplementary Fig. 17a). Based on this coefficient, we used the observed wing length data of adult West and QTP sakers to predict the expected distribution of body mass. We then compared the expected body masses with those observed in the field, and found the observed ones were significantly higher than expected in QTP sakers, but lower than expected in West sakers (Supplementary Fig. 17b). Our results thus suggest a higher BMI in East sakers (QTP + MN). Since BMI is positively correlated with fat content in birds^49^, a high body mass in East sakers may result from either lower physical activities, increased intestinal absorption, higher intake of food^50–52^ or intake of their higher fat-containing mammalian prey (e.g. Brandt’s vole *Lasiopodomys brandtii* in MN and plateau pika *Ochotona curzoniae* in QTP; Supplementary Table 10).

Dietary animal fat (e.g. visceral fat) usually has a high cholesterol level, co-transported with triglyceride by blood lipoproteins^53^, which could affect the blood cholesterol level^54^. Usually, cholesterol from normal diet is sufficient for utilization (e.g. component of cell membrane, precursor of steroid hormones) since about 65% cholesterol is endogenously synthesized^55–57^. In contrast, excessive exogenous cholesterol from a high fat diet will cause an elevated blood cholesterol level^54^ after triglyceride release and absorption by other tissues. We therefore compared the total cholesterol in blood of East and West sakers and found a relatively higher average total cholesterol level in East sakers (Supplementary Fig. 18b). This physiological phenomenon, interestingly, is similar to that reported in polar bears (the ref. 58 and Supplementary Figs. 18a, b).

We further measured the high-density lipoprotein cholesterol (HDLC) and low-density lipoprotein cholesterol (LDLC) concentration in the studied sakers since cholesterols were dominantly bound to high-density lipoprotein and low-density lipoprotein to form HDLC and LDLC in blood. Our results showed that HDLC generally accounted for most bound cholesterols (60% on average; Supplementary Fig. 18c), consistent with observations in other bird species^59^. Interestingly, we found that East sakers had a higher level of HDLC, but comparable LDLC relative to West sakers (Fig. 3b; Supplementary Fig. 18d). The elevated level of cholesterol in blood of East sakers is expected to pose stresses such as atherosclerosis^60^, and it might be expected that East sakers have evolved a strategy to combat this negative stressor.

Indeed, the analysis of adaptive introgressed genes related to lipid metabolism (Supplementary Table 11) appears to reflect adaptation to a colder environment in East sakers. The top one of these enriched genes, *scavenger receptor class B member 1* (*SCARB1*), exhibited the third strongest signal of adaptive introgression across the whole genome (*D* = 0.96, *f*_d_ = 0.82; Fig. 2b; Supplementary Figs. 10, 11). This gene encodes scavenger receptor class B type I (SR-B1) protein which is a surface receptor of hepatocytes and mediates selective uptake of HDLC from blood, contributing to the removal of excessive cholesterol^61^. Further analysis narrowed an introgressed SNP into the 121^st^ amino acid residue of this protein, which is leucine (Leu) in East sakers (*SCARB1*^*362CTT*^) as in gyrfalcons, but proline (Pro) in West sakers (*SCARB1*^*362CCT*^). Alignment of the SR-B1 orthologs among 172 avian species (Fig. 3c; Supplementary Fig. 19) showed that SR-B1^121Leu^ occurred only in gyrfalcons (introgressed to East sakers), suggesting this genetic innovation may have benefits for adaptation to the extreme cold of the Arctic.

To assess the effect of this substitution in East sakers, we performed crystal structure simulations^62^, which suggested that the SR-B1 protein formed a large hydrophobic tunnel transporting lipophilic molecules (e.g. HDLC) into cells, with its *N*- and *C*-terminus forming transmembrane domains anchoring on the membrane. The 121^st^ amino acid is located on a helix, forming a ferrule-like structure that fasten the tunnel (Supplementary Fig. 20), so the substitution of Pro (cyclic structure) with Leu (branched-chain) may loosen the ferrule-like structure and enlarge the tunnel. We therefore hypothesize that the introgressed *SCARB1* mutation leads to a more efficient absorption of HDLC into the liver in East sakers (Fig. 3d**)**.

To test this hypothesis, we compared the extracellular HDLC uptake efficiency into cells expressing, respectively, the West saker wild type (SR-B1^121Pro^) and East saker substituted type (SR-B1^121Leu^) in vitro (Supplementary Fig. 21; “Methods”). Our high-performance liquid chromatography (HPLC) analysis observed a significantly higher cellular cholesterol content in the HeLa cells transfected with SR-B1^121Leu^ plasmids (*P* = 0.017; Fig. 3e), suggesting a higher uptake of HDLC. Our functional results are therefore consistent with our hypothesis that the introgressed SR-B1^121Leu^ enhances the efficiency of blood HDLC removal (Fig. 3d), imparting a lower risk of blood vessels blockage during fat accumulation in East sakers.

Moreover, within East sakers, we found QTP sakers were significantly heavier (also higher BMI) than MN sakers (Supplementary Figs. 9, 17b), coinciding with colder annual mean temperature on the plateau (Supplementary Fig. 8). Notably, both total cholesterol and HDLC levels were also significantly higher in QTP sakers, and no differences in triglyceride (Fig. 3b; Supplementary Figs. 18a, b), suggesting an even higher pressure related to accumulated cholesterol in blood. We found that the introgressed allele *T* on the *SCARB1*^*362*^ (i.e. SR-B1^121Leu^) was subject to positive selection in plateau sakers (Frequency = 0.9 in QTP *vs*. 0.55 in MN) (*hapFLK*^63,64^ test; *P* = 0.03; Supplementary Fig. 22), suggesting that HDLC uptake in QTP sakers was further enhanced by selection of this variation. We propose that this combination of genotype-phenotype has promoted the ability of sakers to cope with QTP’s cold extremes, facilitating their plateau colonization.

### Local adaptation and response to a hypoxic environment

Our analyses revealed a rapid population expansion for QTP sakers, with a 1.4-fold *N*e increase after their arrival on the plateau *ca*. 10 ka (Supplementary Fig. 7). This could be attributed to the loss of ice sheet and the expansion of main food resource (plateau pikas) of QTP sakers^65^ after the LGM. However, such rapid colonization also required surmounting physiological limitations set by low oxygen, and strong UV environment, as well as low temperature, at high elevations.

**Fig. 4:**
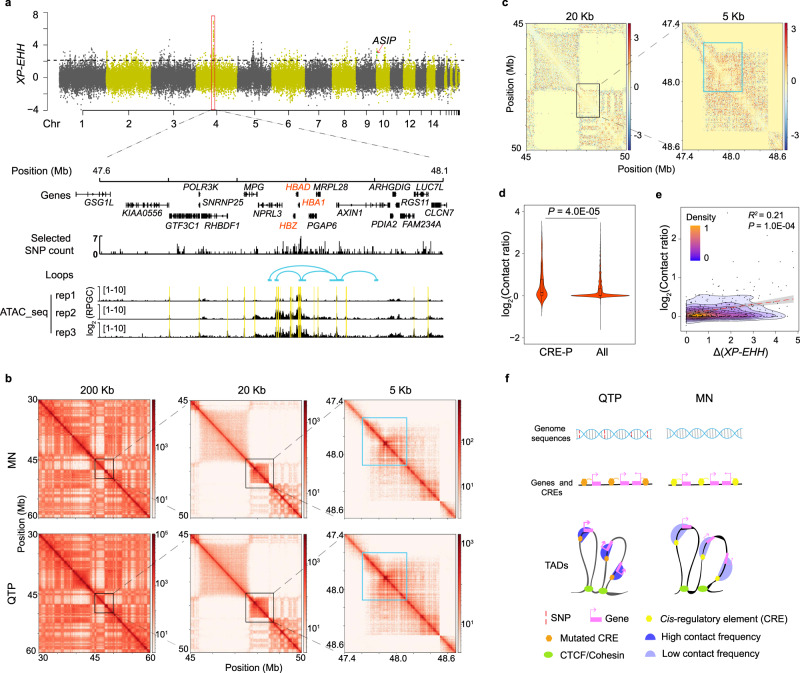
Local adaptation and response of QTP sakers to hypoxia. **a** Positively selected signatures between MN and QTP populations identified by *XP-EHH* (top 1% value = 2.13). The red box shows the focal hard sweep. The window size is 1 Kb. The ATAC-seq tracks (normalized using reads per genome coverage (RPGC) with log_2_ transformed) and Hi-C loops show the *cis*-regulatory elements (CREs) and 3D structure in the focal sweep from blood samples. The yellow blocks show the peaks identified in at least two biological replicates. **b** Hi-C contact maps at bin sizes of 200 Kb, 20 Kb and 5 Kb in the focal sweep. The blue square shows the focal sweep**. c** Hi-C contact maps showing the contact ratio, QTP (*N* = 2) / MN sakers (*N* = 2), in the sweep at bin sizes of 20 Kb and 5 Kb, respectively. The blue square shows the focal sweep. **d** Comparison of contact ratio, QTP (*N* = 2) / MN sakers (*N* = 2), between the bins covering both CREs and gene promoters (P) and all bins in the selective sweep (5 Kb size). A two-sided *Wilcox* test was used. In the box plots, the center line represents the median, whiskers represent maximum and minimum values, and box boundaries represent 75th and 25th percentiles. **e** Correlation between contact ratio and difference of *XP-EHH* values (*XP-EHH* value in QTP population minus that in MN population) for each bin (20 Kb size) across the focal sweep using a linear regression model. The mean (red line) and 95% CI (gray band) are shown. Significance level was calculated using *F* test. Bar shows the density of bin count. **f** A working model showing how the variants affect the chromatin contacts between CREs and promoters. Source data are provided as a Source Data file.

We wished to understand how QTP sakers adapted to their extreme environment in such a short period of time (< 10 ka). We therefore conducted a positive selection analysis between the QTP and MN saker populations across the whole genome using *XP-EHH*^66^ (top 1% value = 2.13; Fig. 4a; Supplementary Data 3) and *F*_ST_ (top 1% value = 0.17; Supplementary Fig. 22). We identified eight selective sweeps containing 27 genes that were significantly enriched for functions in oxygen transport (GO: 0005344, 0019825, 00015671) (Supplementary Table 12), as expected if hypoxia is the most significant stressor for QTP sakers^12^.

The strongest selective sweep covers a ~500 Kb region of Chr 4 (Fig. 4a; Supplementary Data 4). It is outside of the introgressed regions (Supplementary Fig. 10b) and there are no selection signatures in the West saker population (Supplementary Fig. 23), implying that the selection event happened after sakers’ QTP colonization. Of the 293 selective SNPs identified on the sweep, 282 were located in non-coding regions and 11 were coding variations (Fig. 4a; Supplementary Fig. 24; Supplementary Table 13). We thus sought to explore how these non-coding variants could regulate gene expression in QTP sakers. Furthermore, since gene expression regulation, at the genome level, is realized through the contacts between *cis*-regulatory elements that are constrained by the chromatin topology^41^, we examined both *cis*-regulatory elements and chromatin topologies in the focal sweep.

On the sweep, we identified a total of 22 CREs (Fig. 4a; Supplementary Fig. 24) based on our ATAC-seq data from three blood samples of QTP saker (Supplementary Table 8; Supplementary Figs. 13, 25), among which 14 CREs had selected SNPs (*N* = 26). Significantly, most of the 14 CREs were enriched near the cluster of three hemoglobin genes *HBZ, HBAD, HBA1*, together forming a loop that is a small domain in the same TAD^67^. Furthermore, our Hi-C (Supplementary Table 2) analysis showed that all the detected CREs and promoters of other genes embedded in this sweep were co-located within a TAD (bin size = 20 Kb) without changing the TAD boundaries either in QTP or MN sakers (Fig. 4b; Supplementary Fig. 24), so this long selected region shared conserved chromatin structure in the two populations. However, when compared the contact frequency for each CRE and each gene promoter between the two populations, we found that QTP sakers always had a significantly higher contact frequency (*P* = 4.0E–05, *Wilcox* test; Figs. 4c, d; Supplementary Fig. 26). The contact frequency was found to be positively correlated with the strength of selection pressure (*R*^2^ = 0.21, *P* = 1.0E-04; Fig. 4e). Given that the intra-TAD contact frequency correlates with chromatin accessibility^68^, our result indicates that the focal genomic fragment is more accessible in QTP sakers (Fig. 4f).

A change in chromatin openness is expected to alter expression levels of these embedded genes^69^. Since avian blood contains a certain proportion (~10%) of immature erythrocytes (the ref. 70 and Supplementary Fig. 27) that transcribe genes^71^ and the expression pattern in circulating blood is correlated with bone marrow (e.g. chicken, Supplementary Fig. 28), we compared the expression profiles of the embedded genes between plateau and lowland saker blood samples. For the 49 full-length (Iso-Seq) transcripts of 11 genes (Supplementary Table 14) on the sweep, we have identified 14 transcripts from six genes (*HBA1, HBAD, NPRL3, MRPL28, LUC7L, POLR3K*) that were differentially expressed between the QTP and MN populations (Supplementary Fig. 29). Of these, the most highly expressed transcript was *HBA1.2*, accounting for more than 92% of the total *HBA1* expression, and 30% (average) of the whole transcriptome (Supplementary Fig. 30a). This gene was marginally up-regulated in the QTP compared with MN sakers (*TPM* (*Transcripts Per Million*): (3.59 $\pm$ 0.36) E+05 *vs*. (3.06 $\pm$ 0.26) E+05, *q* = 0.01; Supplementary Fig. 30b). We also measured physiological attributes of saker blood samples by principles of colorimetic and electrical impedance^72^, respectively, finding significantly higher hemoglobin concentration (194.83 $\pm$ 18.56 *vs*. 160.50 $\pm$ 25.17 g/L, *P* = 4.5E-03; Supplementary Fig. 31) and comparable hematocrit (HCT, *P* = 0.09, Supplementary Fig. 31) in the QTP relative to MN sakers, consistent with elevated hemoglobin concentration in many plateau birds^70^. We therefore propose that the selected non-coding SNPs of QTP sakers have impacted on the regulation of hypoxia relevant genes in response to plateau hypoxic stress.

### Local adaptation and response to strong UV radiation

**Fig. 5:**
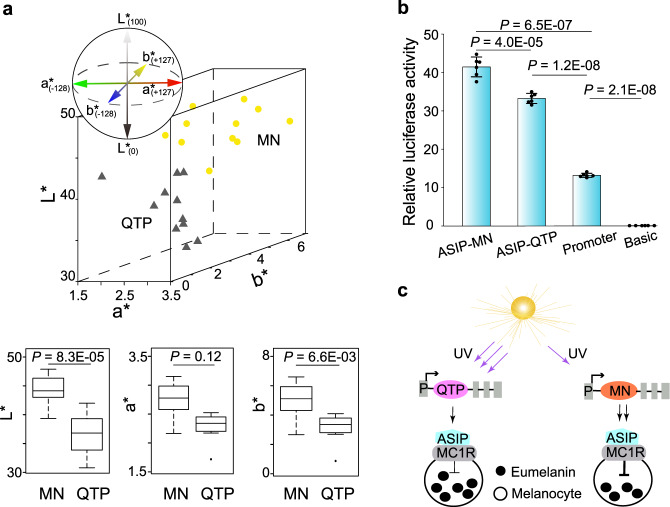
Local adaptation and response of QTP sakers to intense UV radiation. **a** L*a*b* values of plumage color in feathers from MN (*N* = 11) and QTP sakers (*N* = 11). The yellow dots and black triangles represent MN and QTP sakers, respectively. In the box plots, the center line represents the median, whiskers represent maximum and minimum values, and box boundaries represent 75th and 25th percentiles. A two-sided *t* test was used. **b** Relative luciferase activity comparison between dominant MN- and dominant QTP- CREs in duck embryonic fibroblast cells. The ASIP-MN and ASIP-QTP groups were cloned into pGL3-Promoter vectors. Promoter (pGL3-Promoter) and Basic (pGL3-Basic) groups were used as controls, respectively. The bars display mean ± SD (*N* = 6 technical replicates). Three biologically independent replicates of luciferase experiment are shown in Supplementary Fig. 35. A two-sided *t* test was used. **c** A working model showing how dominant MN- and dominant QTP- CREs affect the eumelanin synthesis. “P” means promoter. Source data are provided as a Source Data file.

At high elevations, UV radiation is stronger and can induce DNA damage in the skin^73^. It has been reported that avian feathers provide the first defense line for a bird against UV, because pigments (e.g. melanin and carotenoid) in feathers are able to absorb UV^74^. However, there are few investigations of whether and how feathers protect highland birds from intense UV radiation. To answer this question, we evaluated plumage differences (dorsal, wing and tail feathers) between QTP and MN saker populations using a HR2000CG-UV-NIR spectrometer (Fig. 5a; Supplementary Fig. 32). Our results showed that the lightness (L*) values were significantly lower in QTP chicks (Fig. 5a), suggesting a darker plumage.

To investigate the potential molecular basis of this plumage difference, we scanned positively selected genes in QTP sakers. From the total of 15 genes that have been reported to be related to avian melanin synthesis^75^ (no carotenoids-pigmented feathers in hierofalcon^76^), we have found only one gene under selection, *agouti signaling protein* (*ASIP*) (Fig. 4a; Supplementary Data 3), which suppresses eumelanogenesis by binding to melanocortin-1 receptor (MC1R)^77^. We then detected *cis*-regulatory elements around the *ASIP* gene using ATAC-seq data (Supplementary Table 8; Supplementary Figs. 13, 25) from three QTP saker dorsal skin samples. We found that the chromatin of an intronic fragment with dense variations (ten selected SNPs in 2.1Kb) of *ASIP* gene was accessible in sakers (Supplementary Fig. 33c). Using H3K27ac ChIP-seq data generated from embryonic chicken leg scale skin, we found the orthologous region in chicken is located on the start of a long enhancer-like element (Supplementary Figs. 33b, c). The fragment should be a weak regulatory element as reflected by its regional specific lower intensity either in sakers or chickens (Supplementary Figs. 33b, c). It is also noted that relatively low signal intensity associated with ATAC-seq peaks in saker skin samples is probably because they were collected from dead animals in the field. This *cis*-regulatory element is co-located with the *ASIP* promoter in the same TAD (Supplementary Fig. 33), so it could affect the gene activity. The phasing of this element showed that one haplotype has high frequency (90%) in QTP sakers, in contrast with a medium frequency (40%) in MN sakers (Supplementary Fig. 34).

We compared the activity of this QTP-dominant haplotype with that of the MN-main haplotype by designing a luciferase experiment in CCL-141 cells. Our experiments showed that both haplotypes have enhancing functions (Fig. 5b; Supplementary Fig. 35), however, the QTP haplotype has a lower enhancing effect (*P* = 4.0E–05; Fig. 5b; Supplementary Fig. 35). Thus our results suggested that the QTP dominant haplotype of *cis*-regulatory element causes relatively lower expression of *ASIP*, leading to more eumelanin synthesis in melanocytes. This may comprise a molecular basis for the darker plumage observed in QTP sakers (Fig. 5c) that will limit UV penetration through the feathers and thus minimize UV-induced damage.

## Discussion

Colonization of the QTP by humans and other animals have been explored genetically^78^, but most studies of high-altitude adaptation of QTP animals were conducted simply by comparing highland populations with their lowland counterparts. This produced incomplete or even contradictory evolutionary inferences for many species including humans^79,80^. In this study, we figured out a stepwise colonization of wild sakers onto the highest plateau in the world (Fig. 1a), and untangled the roles of multiple evolutionary processes during this process. We demonstrated that the rapid QTP colonization and adaptation was not realized by simple dispersal from lowland, but took place with different processes (introgression from sister species, natural selection, *etc*.) that played varied spatial-temporal roles in response to different environmental stressors.

The QTP and Arctic share similar extreme weather conditions, and similar morphological traits have been observed in animals from both poles^1^. Combining paleo-climatological, ecological and genetic evidence, we found that a secondary contact occurred between Arctic-adapted gyrfalcons and Asian sakers in the LGM, and this hybridization allowed ancestral sakers to develop larger body size and helped overcome negative effects of higher fat storage by more effectively removing excessive cholesterols from blood. This may facilitate sakers to cope with cold stress in eastern Eurasia and also predispose their survival in even colder QTP environments.

Recently, numerous studies have suggested important roles for key genes in high-altitude adaptation or response, but few have addressed the roles of *cis*-regulatory elements and their interaction with target genes^81^. Our comparative 3D genome analyses identified a hard sweep within which expression of multiple genes was regulated through altering chromatin interactions of *cis*-regulatory elements (e.g. enhancers, suppressors) and gene promoters within a TAD. Notably, this may represent a general paradigm of gene regulation for non-coding variants because we also observed similar patterns in three other selective sweeps associated with hypoxia and UV responses (Supplementary Fig. 36). Natural selection, may favor a conserved mode that not only clusters genes with similar functions, but also constrains genes and regulatory elements within a higher-order genome architecture such as TAD.

Qinghai-Tibet Plateau is commonly conceived as a ‘natural laboratory’ for organism adaptation to extreme environments, but there are still few systematic investigations of the three main stresses (hypothermia, hypoxia and strong UV radiation). Our work shows that adaptation to environmental extremes on the ‘third pole’ has resulted from adaptive introgression from hypothermia-adapted Arctic relatives (bigger body size and resistance to high fat loads), as well as local adaptation or response to hypoxia mainly through changes on higher-order genome architecture and UV protection high likely through melanin production. Identification of essential genes relevant to stress resistance (e.g. *SCARB1* target lipid-lowering drug) opens new avenues to investigate the genomics of adaptation, and may also have potential medical applications in future. Since saker falcons prefer cooler habitats in east Eurasia, our study will also be relevant to the conservation of QTP sakers (supporting the largest wintering population^30^), especially in the context of ongoing global warming^82^.

## Methods

### Ethics oversight

All lab experiment procedures were under the guidance of the Ethics Committee of the Institute of Zoology, Chinese Academy of Sciences. The collection and processing of falcon tissues in this study were conducted in accordance with the guidelines of Institutional Animal Care and Use Committee of the Institute of Zoology, Chinese Academy of Sciences.

### *De novo* assembly of the saker falcon genome

We assembled a chromosome-level reference genome of an adult female saker falcon (a QTP saker rescued by Xining Wildlife Park) (2*n* = 52) using a multi-platform sequencing strategy (PacBio, Illumina, Bionano).

First, genomic DNA was extracted from a blood sample following the protocol of Blood & Cell Culture DNA Midi Kit (QIAGEN) and quantified by Qubit 2.0 Fluorometer (Thermo Fisher Scientific). A long-read library was constructed through 26G Needle fragmentation, fragments selection (20 Kb), adaptor linkage and subjected to sequencing on a PacBio Sequel System (Pacific Biosciences)^83^. The output raw subreads were filtered using *SMRTlink* (version 5.0, https://www.pacb.com/support/software-downloads/) with parameters “-minLength 50 -minReadScore 0.8”. The filtered subreads were used for the contig assembly by *wtdbg2*^84^ (version 1.2.8) with parameters “–tidy-reads 5000 –edge-min 4 –rescue-low-cov-edges”, followed by two rounds of polishing using *wtdgb-cns*. The assembled contigs were corrected using all the subreads by *Pbalingn* with parameters “–minAccuracy 70.0 –minLength 100 –hitPolicy randombest” and the consensus sequences were obtained by *variantCaller* with parameters “–minConfidence 40 –minCoverage 5 –algorithm arrow”. We further re-corrected the consensus sequences with 89.58 Gb short reads generated by a HiSeq X Ten sequencer (Illumina) using *BWA*^85^ (version 0.7.12) and *pilon*^86^ (version 1.22) to obtain a primary genome assembly.

Second, we used an optical mapping method to order and orient the primarily assembled contigs to scaffolds and validate the assembly^87^. Briefly, the isolated megabase genomic DNA was labeled following the Nick-Label-Repair-Stain protocol with Nt.BspQI enzyme to construct libraries, which were subjected to sequencing on a Bionano Saphyr System (BioNano Genomics). The raw sequenced molecules were filtered with: (1) length < 150 Kb; (2) molecule SNR (ratio of brightness of DNA intercalator to background noise for the dsDNA molecule (Signal to Noise)) < 2.75 and label SNR (corresponding ratio of label brightness) < 2.75; (3) label intensity > 0.8. The clean data were aligned with the assembled contigs using *Solve* (version 3.1) with parameters “pipelineCL.py -i 1 -minlen 150 -minsites 11 -MapRate 0.45”. The mapped molecules were used to correct and link these contigs into scaffolds.

Third, we performed Hi-C^88^, an all-*vs*-all chromosome conformation capture technique, to assign the scaffolds to groups (super-scaffolds). The Hi-C libraries were constructed sequentially through formaldehyde crosslinking, *Mbo*I enzyme digestion, biotin marking, ligation and purification^89^. The qualified libraries (200–600 bp insert size) were then subjected to sequencing on a HiSeq X Ten platform. The raw sequencing data were filtered by removing reads with: (1) low quality data (more than 50% bases with *PHRED* values < 19); (2) ‘*N*’ rate higher than 5%; (3) adaptor sequences. The clean data were aligned with the assembled scaffolds using *HiC-Pro*^90^ (version 2.7.8) to retrieve the uniquely mapped paired-end reads to identify chromatin interactions. *LACHESIS*^91^, a method based on the agglomerative hierarchical clustering algorithm, was used to cluster, order and orient scaffolds to super-scaffolds according to the interaction information.

Finally, we identified the macro-chromosomes (length > 50 Mb) and micro-chromosomes (length < 50 Mb) from super-scaffolds by aligning the saker assembly against with those of *Aquila chrysaetos* (golden eagle)^92^, *Falco peregrinus* (peregrine falcon)^18,93^, *Gallus gallus* (chicken; GRCg7b) and *Deomaius novaehollandiae* (emu)^94^ using the method we have developed^19^. To further confirm W chromosomal sequences, we compared the sequencing depth of each assembled chromosome for each resequenced individual. The assembled sequences with depth less than one in male falcons but half of the mean whole genome sequencing depth in females were considered as W chromosome sequences.

### Gene annotation

Gene prediction was performed using our previous pipeline^18^. Briefly, for the homolog predictions, we first masked the transposable elements of the assembled genome using *RepeatMasker* (version 4.0.8, http://www.repeatmasker.org/). Then, we mapped the protein sequences of *Gallus gallus, Taeniopygia guttata, Falco peregrinus* against the assembled genome using *TBLASTN*^95^ (version 2.2.23) with an E-value threshold of 1E-05 and determined gene models using *GENEWISE*^96^ (v2.2.0). For the transcriptome-based prediction, we aligned the blood transcriptome data with the assembled genome using *Tophat*^97^ (version 2.1.2) and identified transcripts using *Cufflinks*^98^^.^ For the *de novo* predictions, we trained the parameters using the well annotated genes from homolog and RNA-seq evidence. The trained parameters were used to predict candidate genes using *AUGUSTUS*^99^ (version 2.5.5) and *GENESCAN*^100^ (version 1.0). Function annotations were conducted by aligning each protein sequence to SwissProt and TrEMBL databases^101^ using *BlastP*. Domains of genes were searched using *InterProScan*^102^ (version 4.7).

### Sampling and genomic DNA extraction for genome resequencing

A total of 30 saker samples (27 blood and three plucked chick feathers) were collected in the wild. These included two MD, three CE, five SK, 10 from MN and 10 QTP individuals. Approximately 0.2 mL fresh blood was collected from each bird and immediately put into the vacutainer containing 7.2 mg of K2 EDTA (BD). Plucked chick feathers were collected and stored in 75% ethanol. In addition, blood samples of 10 wild gyrfalcons were collected from Arctic Russia with three individuals from Kola, three from Yamal and four from Chukotka. These samples were collected during our field work from 2007 to 2017.

DNA was extracted using DNeasy Blood & Tissue kit (QIAGEN) and quantified by Qubit 2.0 Fluorometer. One library with 350 bp insert size was constructed for each sample following the manufacturer’s protocol and subjected for sequencing on a HiSeq X Ten platform. The raw sequencing data were filtered by removing (1) low quality reads (more than 50% bases with *PHRED* <7); (2) ‘*N*’ rate higher than 5%; (3) reads with adaptors.

### SNP calling

The clean reads of each individual were aligned against the assembled saker genome using *BWA*. The reads with low mapping quality (*PHRED* < 20) were excluded using *Samtools*^103^ (version 1.9) and duplicates were removed using *Picard* (https://www.broadinstitute.github.io/picard/) (version 1.95). The variants were identified using the Genome Analysis Toolkit^104^ (GATK, version 3.3.0) (parameter “-stand_call_conf 30”) and filtered with parameters “MQ0 > 1 or MQ < 30 or BaseQRankSum < −8 or ReadPosRankSum < −8 or FS > 40”. Alleles that were not covered in all samples and minor allele frequency (MAF) less than 0.05 were removed.

### Demographic history reconstruction and relative cross coalescent rate estimation

We used *SMC++*^20^ (version 1.9.2) to reconstruct the demographic histories of falcons. The autosomal unphased bi-allelic SNPs of each falcon were used to infer the demographic history using *SMC++* with 20 EM iterations and 1E-4 for the threshold of terminating the EM algorithm. 100 bootstraps were conducted.

For the estimation of relative cross coalescent rate using *MSMC* (version 2.1.2)^28^, we first phased the genotypes using *BEAGLE*^42^ (version 4.1), which applies a Hidden Markov model (HMM) to locally cluster the haplotypes. We then randomly selected eight phased haplotypes to infer relative cross coalescent rates between each two genetically separated populations.

### Population structure detection

We used the autosomal bi-allelic SNPs to detect the potential population genetic structure using the PCA method and a maximum likelihood approach *Frappe*^105^ (version 1.1). To reduce the impacts of linkage disequilibrium (LD) on the analysis, only intergenic sites for which the distance of any two neighboring sites was at least 10 Kb were considered. For PCA, we converted eigenvectors from the covariance matrix (calculated from the SNP matrix) by *R* function *EIGEN* and examined significance by *Tracy-Widom* test implemented in *EIGENSOFT*^106^ (version 3.0). For *Frappe*, genetic cluster *K* was predefined from 2 to 6 without assuming any prior information and the maximum iteration of expectation-maximization was set as 10,000.

### Phylogeny of the W chromosome

The variants on the W chromosome were identified using the *BWA/GATK* pipeline above mentioned. Considering the W chromosome is haploid except for PARs (regions homologous to Z chromosome of saker genome), we masked the SNPs in PARs and gene regions^107^, and retained neutral haploid SNPs that existed in each individual by filtering the loci potentially affected by purifying selection (MAF < 0.05)^108,109^. A phylogenetic tree was reconstructed using a neighbor-joining method with *p*-distance by *fneighbor* implemented in *EMBOSS* package^110^ with a female peregrine as the outgroup^111^ and the tree was plotted by *Figtree* (version 1.4.3, http://www.tree.bio.ed.ac.uk/software/figxtree/).

### Contributions of female and male gyrfalcons to the gene pool of ancient East saker population

Since the W chromosome lacks recombination (except for PARs), which would not cause the loss of ancient introgressed alleles, the contribution of female gyrfalcons to the ancient gene pool of female East sakers could be estimated by the observed proportion of female East sakers that were clustered with female gyrfalcons in the W chromosomal phylogeny. We assumed that the effects of genetic drift were negligible since the effective population size of East sakers was large and increasing after the LGM (Fig. 1c). In addition, we removed the variants with MAF < 0.05 to exclude the potential effects of purifying selection, the main selection model in avian W chromosome^108,109^. After assuming that the adults in ancient East saker populations had the same sex ratio (1: 1) as present^30^, the contribution of female gyrfalcons to the gene pool of ancient East saker populations was half of the proportion.

Also, we assumed recombination will lead to loss of ancient neutral introgressed alleles at a constant rate *r* per generation, the proportion of the ancient East saker gene pool contributed by male gyrfalcons could be estimated following the two formulas:

1 $$\frac{1}{2}(x+y){(1-r)}^{N}=A$$

2 $$\frac{1}{3}y+\frac{2}{3}x{(1-r)}^{N}=Z$$

where *x* and *y* represent the contributions of male and female gyrfalcons to the gene pool in the ancient East saker population at the time when hybridization ceased, *r* the rate at which ancient introgressed alleles are lost per generation, *N* the number of generation estimated by (the time when hybridization ceased to now) / (generation time of the saker falcon), *A* and *Z* the proportions of introgressed alleles observed in autosome and Z chromosome at present. In the formula (1), the coefficient 1/2 means half of the autosomal genetic material comes from males and the other half from females. In the formula (2), the coefficients 1/3 and 2/3 mean 1/3 of the genetic material on Z chromosome comes from females and 2/3 from males.

### Admixture estimation by *f*_3_-statistic

The *f*_3_-statistic implemented in *Admixtools* (version 5.1)^23^ emerges from a test of three populations (A; B, C) that explicitly asks whether A, is the result of admixture between B and C. It measures the covariance of the differences in allele frequencies of A–C and B–C population pairs across all genomic loci. If *f*_3_(A; B, C) is significantly negative, then B and C contribute to the admixed population A. Here, we used West saker to proxy the ancestral East saker to test *f*_3_ (East saker; gyrfalcon, ancestral East saker).

### Admixture time estimation

To estimate the admixture time, we used a local ancestry inference method *Ancestry_HMM*^24^ (version 0.94) to trace the ancestry of discrete genomic segments. We fitted a single pulse admixture model to genome-wide variation data^112,113^ and gave the ancestry types in the introgressed populations (East saker) with the one (saker type) with the proportion of 0.7 and the other (gyrfalcon type) with the proportion of 0.3. We quantified uncertainties by 500 bootstraps.

### Simulation of potential breeding areas for gyrfalcons and sakers

To predict potential breeding areas for gyrfalcons and sakers, we performed an Ecological Niche Modeling (ENM) analysis using *MaxEnt*^26^ (version 3.3.3k) in the *R dismo* package. We downloaded the occurrence data of sakers and gyrfalcons from GBIF (https://www.gbif.org/). The breeding records were limited to those from June to August to avoid bias due to the presence of potential migrants. For the GBIF data, we firstly removed the occurrence points located in ocean or having low accuracy. Then, we removed the points if there is only one individual recorded. Finally, we used a spatial filter distance of 40 km between the points to minimize the effects of over-sampling. Due to the limited data for gyrfalcons in GBIF, we additionally used 79 randomly selected occurrence sites in breeding areas across Eurasian Arctic reported in two previous studies^17,114^.

For climate variables, we downloaded the bioclimate variables (0.5° resolution) from a previous study^115^ and cropped the spatial extent of the ENMs to include all known occurrence sites, covering an area ranging from 10° S to 90° N and 20° W to 180° E. To train the model, we selected 80% of occurrence sites to fit the *MaxEnt* species distribution model, and kept the remaining 20% sites for model testing. We used all layers to predict the current distribution and then selected the variables which contributed greater than 10% to the predicted distribution. Finally, we projected the ENMs built under current climate to paleoclimates, including the LIG and LGM. To account for the uncertainty of paleoclimates on single snapshot, we selected paleoclimates of multiple snapshots at the LGM (20 to 27 ka) and LIG (110 to 114 ka) to project the ENMs and then calculated the maximum suitability scores by using corresponding snapshots. The present breeding areas of saker and gyrfalcon were obtained from two previous studies^11,18^. To calculate the overlapping breeding areas between sakers and gyrfalcons during the LGM, we cropped the spatial extent to an area ranging from 46° N to 60° N and 68° E to 98° E, and calculated the overlapped area in QGIS (http://www.qgis.osgeo.org/).

### Simulation of colonization scenarios

We used the software *fastsimcoal2*^32^ (version 2.6) to simulate colonization scenarios of saker populations, taking account of the inferred hybridization event. We used *easySFS* (https://www.github.com/isaacovercast/easySFS) package to convert the unlinked autosomal intergenic SNPs (one SNP per 10 Kb) to the site frequency spectrum and fed it into *fastsimcoal2*. The parameters (Supplementary Table 7) were set based on the detected population structure (*K* = 4; Fig. 1b; Supplementary Fig. 4), population histories (Fig. 1c; Supplementary Fig. 6) and the identified introgression time with gyrfalcons. The software was run with 1,000,000 coalescent situations and 40 ECM cycles. The model with the largest maximum likelihood was selected.

### Biometric data of falcons

We measured the wing lengths of 19 adult QTP saker specimens (11 females and eight males): 11 from the National Zoological Museum of China, three from the Northwest Institute of Plateau Biology, Chinese Academy of Sciences, one from the Xinjiang Institute of Ecology and Geography, Chinese Academy of Sciences, one from the Tibet Museum of Natural Science and three from our field surveys. We measured the body masses of eight adult females (one specimen and seven wild falcons) and ten males (one adult specimen and nine fledging birds) in the QTP population. The adult specimens were collected from the Qinghai-Tibet Plateau during multiple surveys from 1935 to 2007. Our field surveys on the QTP were conducted from 2018 to 2020. The wing length and body mass of Mongolian adult specimens were obtained from the field surveys in 2013-2014^48^. The wing length and body mass of West sakers and gyrfalcons were derived from previous references^116–119^. The correlation between biometrics and temperature was modeled using a linear regression model. The correlation between body mass and wing length in MN sakers was also obtained using a linear regression model, and the expected and observed body masses were compared using *t* tests.

### Estimation of adaptively introgressed signals and discrimination from ILS

We used the ABBA-BABA model^37^ to test the Patterson’s *D* statistic and *f*_d_ values in 100 Kb sliding window size with 50 Kb step size along the autosomes. The (((P1, P2), P3), O) topology was set as (((West saker, East saker), gyrfalcon), peregrine). We considered fragments in the top 1% *D* and *f*_d_ values as candidate regions adaptively introgressed from gyrfalcons to East sakers. Introgressed genomic islands were determined as a cluster of at least three consecutive adaptively introgressed regions. To confirm the detected introgression, we also calculated the fixation index (Weir and Cockerham’s *F*_ST_) using *VCFtools* (version 0.1.13, https://www.vcftools.github.io/), and the genetic divergence *d*_XY_^120^ between gyrfalcons and East sakers, gyrfalcons and West sakers, West and East sakers, respectively, and nucleotide diversity *θ*_*π*_ for each of the three clusters with a sliding window size of 20 Kb by using *VCFtools*. We considered true signals as those regions with significantly different *F*_ST_/*d*_XY_ in the gyrfalcon/West saker and West/East saker comparisons but not in the gyrfalcon/East saker comparison.

We used a strategy described below to determine whether the signatures identified above resulted from introgression or ILS. Because ILS blocks were randomly distributed in the genome and fragmented following recombination, their lengths should be shorter than those fragments caused by introgression^121^. Given an observed length of a fragment, we calculated its probability as an ILS block using the formula *P* = exp (-*k*/*L*)^122^, where *L* is the expected length of a shared sequence between East sakers and gyrfalcons, which equals [(1 - *m*) *r* (*t -* 1)]^−1^ (*t* is the admixture time in generations, *m* is the admixture fraction and *r* is the recombination rate per base pair per generation). In our case, we set the parameters according to the *fastsimcoal2* result: *t* = 3,181 generations (21 ka), *m* = 0.213 (the minimum introgression rate in *Frappe* result), *r* = 2E-08 (assuming that each of the 26 chromosomes experiences on average one crossover per generation^123^). When the *P* was larger than 0.05, the fragments shorter than 26.0 Kb were considered as those that may be influenced by ILS. Accordingly, fragments longer than 26.0 Kb were considered from introgression.

### Luciferase reporter assay

We used the luciferase reporter assay to validate the activities of the target REs of *SCMH1* and *ASIP* genes. The primers were designed using *PRIMER3*^124^. For the *SCMH1* gene, the primers were: forward, 5ʹ- CGACGCGTGGTGATGATGGTTCATGGGTG −3ʹ and reverse, 5ʹ- GAAGATCTGCTAAACGTGCACCTTCCTTT −3ʹ. For the *ASIP* gene, the primers were: forward, 5ʹ- CGACGCGTACAGAGGTAAGTGCACCAG −3ʹ and reverse, 5ʹ- GAAGATCTTTATTTTCTTCCTTTTCAACCC −3ʹ. The primers were used to amplify the target sequences from genomic DNA extracts (dominant introgressed and dominant wild haplotypes of *SCMH1* gene were amplified from MD1 and QTP7, respectively; main MN- and dominant QTP- haplotypes of *ASIP* gene were amplified from MN6 and QTP7, respectively). The amplified DNA was cloned into the pGL3-Promoter vector (Promega) digested with *Mlu*I and *Bgl*II. After confirmed by Sanger sequencing, the successfully constructed plasmids were isolated using Endo-free Plasmids Maxi Kit (OMEGA). The pGL3-Basic and pGL3-Promoter plasmids were used as controls. When the CCL-141 cells grew up to 70% confluent in the 24-well plate (Falcon), the constructed and control plasmids were respectively co-transfected into the cells together with pRL-TK (Promega) using Lipofectamine 2000 (Invitrogen). Cell lysis was collected using the Dual-Glo Luciferase Assay Kit (Promega) for the following assessments after 24 hours. The normalized luciferase activities were measured using the Dual-Luciferase Report Assay System (Promega) and GloMax® Explorer Multimode Microplate Reader (Promega) according to the manufacturer’s instructions. Experiments were performed in hexaplicates and independently repeated three times.

### Measurements of triglyceride and cholesterol content in plasma

We took blood samples from 17 saker chicks at 4–6 weeks old (including five SK, six MN and six QTP sakers) for the measurements of lipid components including total triglyceride, total cholesterol, HDLC and LDLC. Blood was centrifuged at 500 × *g* for 10 min at 4 °C. The contents of each lipid components were measured using assay kits^125^ (BioSino Bio-technology and Science Inc.).

In brief, for the triglyceride assay (GPO-PAP method), Reagent1 and Reagent2 were mixed and reacted with plasma to hydrolyze triglyceride. The generated glycerin was then reacted with enzymes to produce quinone imine followed by measurements of the absorbance values at 505 nm using a SpectraMax i3 microplate reader (Molecular Devices). Triglyceride standard and water were used as control and blank respectively. The triglyceride content was determined by the differences in absorbance as:

3 $$\frac{{Sample}-{blank}}{{Control}-{blank}}\,\times \,{TG}\,{standard}\,{concentration}$$

For the total cholesterol assay (CHOD-PAP method), Reagent1’ and Reagent2’ were mixed and reacted with plasma to degrade cholesterol to quinone imine followed by measurements of the absorbance values at 505 nm. Cholesterol standard and water were used as control and blank respectively. The cholesterol content was determined using a formula below:

4 $$\frac{{Sample}-{blank}}{{Control}-{blank}}\,\times \,{TC}\,{standard}\,{concentration}$$

For the HDLC assay, Reagent1* (R1) was reacted with plasma to remove chylomicron, low-density lipoprotein cholesterol and very-low-density lipoprotein cholesterol, followed by the absorbance value measurement at 600 nm. Reagent2* (R2) was then mixed with the above residuals to release the HDLC, again followed by the absorbance value measurement at 600 nm. The HDLC concentration was determined by the differences in absorbance as:

5 $$\frac{\left(R2-R2\,{blank}\right)-(R1-R1\,{blank})}{\left(R2\,{control}-R2\,{blank}\right)-(R1\,{control}-R1\,{blank})}\\ \qquad \times \,{HDLC}\,{standard}\,{concentration}$$

For the LDLC assay (surfactant assay), Reagent1^#^ (R1) was reacted with low-density lipoprotein and protected the loaded cholesterol that was not degraded by enzymes, followed by measurements of the absorbance values at 600 nm. Reagent2^#^ (R2) was then mixed with the above residuals to release the LDLC, and was measured absorbance at 600 nm. The LDLC concentration was determined using a formula below:

6 $$\frac{\left(R2-R2\,{blank}\right)-(R1-R1\,{blank})}{\left(R2\,{control}-R2\,{blank}\right)-(R1\,{control}-R1\,{blank})}\\ \qquad \times \,{LDLC}\,{standard}\,{concentration}$$

Each sample was repeatedly measured three times for each experiment.

### Protein structure prediction of SR-B1

We annotated the protein sequences of *SCARB1* gene in a total of 319 avian species from previous references^126,127^ and this study. A multiple sequence alignment on these SR-B1 protein sequences was conducted using *MAFFT*^128^ (version 7.407).

We predicted the SR-B1 protein (belongs to the CD36 family) structure of falcons using *SWISS-MODEL*^129^ with the human homologous protein LIMP-2 (code: 4F7B)^130^ modeling as templates. The transmembrane domains of *N*- and *C*- terminals in the falcon’s SR-B1 proteins were predicted using *TMHMM* (version 2.0, http://www.cbs.dtu.dk/services/TMHMM/).

### Evaluation of the cholesterol uptake efficiency of SR-B1 proteins

To evaluate the cholesterol uptake of the wild type SR-B1^121Pro^ and mutated type SR-B1^121Leu^, we performed an over expression experiment in vitro using the method modified from Zanoni’s^61^. The full-length cDNA sequences of *SCARB1*^*362CCT*^ and *SCARB1*^*362CTT*^ were synthesized (without the stop codon) and cloned into pEGFP-N1 vectors (Clonetech) separately, with GFP expressed at the *C*-terminus as the index of protein expression. The successfully constructed plasmids were verified by Sanger sequencing. The human HeLa cells (CCL-2, ATCC) were cultured in Dulbecco’s modified Eagle’s medium (Hyclone) supplemented with 10% fetal bovine serum (FBS; Gibco; HDLC-enriched) and 1% Penicillin-Streptomycin (P/S) (Gibco) (complete medium) at 37 °C in a humidified 5% CO_2_ incubator and passaged using trypsin. Cells were then plated at a density of 9.5 × 10^5^ cells/cm^2^ in 6-well plates (each well 9.6 cm^2^) and prepared for transfection when they grew up to 90% confluency. The control (empty vector), *SCARB1*^*362CCT*^ and *SCARB1*^*362CTT*^ plasmids were transfected respectively using Lipofectamine 2000 (Invitrogen) following the manufacturer’s instructions. The transfected cells were incubated for 24 hours and assayed through GFP expression under an Eclipse Ti-s fluorescence optical microscope (Nikon). The successful expression of transfected SR-B1-GFP fusion proteins was further confirmed by Western blotting using GFP antibody (1: 10000, Abcam, ab183734) with β-Actin (1: 5000, Gene-Protein Link, P01L03) as the internal control. The secondary antibodies goat anti-rabbit IgG-HRP (1: 10000, Gene-Protein Link, P03S02S) and horse anti-mouse IgG-HRP (1: 3000, Cell Signaling Technology, 7076) were used for GFP and β-Actin respectively. The media was then removed, and the cells were washed three times with PBS, and harvested in 800 μL 0.9% NaCl. After lysing the cells and centrifuging (4 °C, 6210 × *g*, 10 min), the supernatant was obtained for measuring the protein content by a bicinchoninic acid (BCA) assay (Beyotime) and cholesterol content by an HPLC method, respectively.

Here, for the HPLC assay, we first extracted 400 μL cell suspension, added an equal volume of 15% KOH ethanol solution, vortexed for 5 min, and incubated in a 60 °C water bath for an hour. Next, we added 100 μL trichloroacetic acid and vortexed for 5 min, and added 400 μL of hexane-isopropanol mixture (3: 2, vol/vol). After centrifugation with 14,850 × *g* for 20 min at 4 °C, the solvent fraction was collected. The collected sample was dried under a stream of nitrogen and resuspended in 400 μL acetonitrile-isopropanol mixture (1: 1, vol/vol), centrifuged at 14,850 × *g* for 20 min at 4 °C. 20 µL suspension were extracted and injected into a LC-30A UHPLC system (Shimadzu) with Eclipse XDB-C18 column (4.6 mm × 250 mm, 5.0 µm) to examine the cellular cholesterol content. The samples were eluted with acetonitrile-isopropanol mixture (1: 1, vol/vol) for 10 min and the peak of cholesterol was detected at 206 nm. During the measurement, the column temperature was set at 40 °C and the flow rate was 1 ml/min.

The peak area of each sample was used to evaluate the abundance of cholesterol according to a standard calibration curve, which was obtained based on six standard concentrations (2, 5, 10, 60, 80 and 100 ng/μL) of cholesterol (Sigma-Aldrich).

Each experiment was repeated independently three times.

### Detection of positively selective signatures

We used a cross-population extended haplotype homozygosity (*XP-EHH*) method implemented in the *Selscan*^66^ (version 1.2.0) to identify the recent positively selected signals in a sliding window of 20 Kb between MN and QTP saker populations. The windows with top 1% *XP-EHH* values were considered as positively selected regions in QTP sakers. Hard sweeps were determined as a cluster of more than five consecutive windows with top 1% *XP-EHH* values. *F*_ST_ and *hapFLK* (version 1.4) methods were further used to confirm the selection signals. GO category enrichment of positively selected genes in QTP sakers was conducted using a *Chi-square* test and adjusted by False Discovery Rate (FDR) method (*q* < 0.05)^131^. The GO terms were excluded if the enriched gene number was less than three.

### Identification of immature erythrocytes in avian circulating blood and correlation analysis of gene expression between chicken blood and bone marrow

We examined the proportion of immature erythrocytes in avian blood by the Giemsa stain method^70^. About 50 µL blood was extracted from each of three saker falcons (one 6 months-old and two 4.5 years-old) and three budgerigars (*Melopsittacus undulatus*) (aged 1.5, 3 and 6 months-old), respectively. Five blood smears were produced from each individual, stained by Giemsa (Yeason) and scanned at × 40 magnification using Aperio VESA8 system (Leica). For each smear, more than 700 cells were randomly selected for counting and identifying the immature erythrocytes^70,132^.

We downloaded the chicken transcriptomes of blood (14-days-old and 35-weeks-old) and marrow tissues (19-days-old, 4-weeks-old and 8-months-old) from NCBI and compared these data to further check whether gene expression in circulating blood could proxy that in bone marrow. The RNA-seq data used in this analysis are available in the NCBI database under accession codes PRJNA542984, PRJEB44038, PRJNA323973, PRJNA279487, PRJNA412404, respectively. The sequences were aligned with the chicken genome (galGal4) using *Bowtie2*^133^ (version 2.3.4.3). The expression of each transcript was quantified as *transcripts per million* (*TPM*) using *RSEM*^134^ (version 1.3.1). After filtering the lowly expressed transcripts (*TPM* <1), the expression correlation between transcriptome of blood and bone marrow samples was calculated using ggcorrplot package in *R*.

### Detection of genome-wide chromatin accessibilities

In order to study the genome-wide chromatin accessibilities of sakers, we performed ATAC-seq for different tissues. We collected three blood samples from QTP saker chicks (5–6 weeks old). The blood was subjected to centrifugation at 4 °C, 800 × *g* for 10 min. After removing plasma, the remaining cells were washed by PBS. Each of 1 µL blood cells were mixed with 1 mL freeze medium (90% FBS and 10% DMSO). We dissected an embryo from an un-hatched egg (about 28 days incubated) of QTP saker and sequenced tissues of forelimb, keel and flight muscle. We got the dorsal skin from three QTP saker juveniles which naturally died in the field in 2019 and 2022.

The ATAC-seq libraries for these samples were constructed following the Omni-ATAC protocol^135^. Lysate buffer was added to obtain the nuclei and the libraries were conducted by *Tn*5 enzyme transposition mix buffer reaction. The qualified libraries were subjected to pair-end sequencing on a Novaseq 6000 platform (Illumina). The raw sequences were filtered by *Trimmomatic*^136^ with default parameters.

To detect the chromatin accessibility, clean reads were aligned with the saker genome using *BWA*. The reads with low mapping quality (*PHRED* < 20) were filtered and duplicates were removed. The candidate accessible open chromatin regions (peaks) were identified using *MACS2*-callpeak^137^ with parameters “-nomodel -f BAMPE -p 0.05”. For the blood samples, the reproducible peaks were identified using an irreproducibility discovery rate (IDR ≤ 0.05) method^138^ and the signals were kept when it occurred in at least two samples. For the dorsal skin samples from dead sakers, the reproducible peaks were identified using BEDTools^139^ (version 2.25.0) with overlapping rate of peaks larger than 50% in at least two samples.

ATAC-seq data of chicken bone and muscle tissues were downloaded from NCBI (data are available in the NCBI database under accession code PRJNA433154) to find the *cis*-regulatory elements around *SCMH1* gene. H3K27ac ChIP-seq data of chicken leg scale skin samples were downloaded from NCBI (data are available in the NCBI database under accession code PRJNA561632) to identify the *cis*-regulatory elements around *ASIP* gene. The downloaded data were aligned with chicken genome (Galgal4) using *BWA* and peaks were identified using *MACS2*-callpeak. The homologous *cis*-regulatory elements in chickens were identified by aligning the assembled saker genome sequences against the chicken genome (Galgal4) using *LASTZ*^140^ (version 1.04.00).

We have normalized all of the ATAC-seq using reads per genome coverage (RPGC) calculated from *bamCoverage* (*deepTools*^141^, v3.5.0), and ChIP-seq data using read count ratio (log_2_ scale) between H3K27ac and input data calculated from *bamCompare* (*deepTools*, v3.5.0) respectively to show the tracks.

### Detection of chromatin architectures using the Hi-C technique

To compare the chromatin architectures of the focal sweep between MN and QTP sakers, we performed Hi-C sequencing on blood samples of QTP (*N* = 2) and MN chicks (*N* = 2) (aged 5–6 weeks old). The sequenced reads were aligned with the assembled saker genome using *HiC-Pro*. *HiCExplorer3*^142^ was used to generate the contact matrix, identified TADs and computed contact ratio. The loops were identified using *HICCUPS*^143^ (v1.0.0). The TADs of *SCMH1* and *ASIP* genes were identified following the *HiC-Pro/HiCExplorer3* pipeline. The correlation between contact ratio (QTP/MN) and difference of *XP-EHH* values (*XP-EHH* value in QTP population minus that in MN population) for each bin (20 Kb size) was simulated using a linear regression model. We also compared the contact ratio of QTP/MN sakers (log_2_ scale) between the TAD region and flanking 500 Kb regions, between the TAD region and the whole Chr 4, and between the TAD region and the whole genome, respectively, for testing the enhanced chromatin interactions of the 500 Kb focal sweep.

### Identification of full-length transcripts and differentially expressed transcripts

We performed an Iso-Seq using a PacBio platform from a QTP saker chick (aged 5–6 weeks old) to obtain full-length transcripts in blood. The sequenced reads were aligned with the assembled saker genome assembly using *Minimap2*^144^ (version 2.13). After filtering the low quality mapping (*PHRED* < 10) reads and removing duplicates, we identified the gene isoforms using *cDNA_Cupcake* (version 5.8, https://www.github.com/Magdoll/cDNA_Cupcake). To identify the differentially expressed transcripts **(**DETs) in the focal sweep between MN and QTP saker populations, we calculated the expression of each transcript from our published blood RNA-seq data (data are available in the CNCB database under accession code PRJCA008052^12^) (the MN population transcriptomic data were generated from samples collected in central Asia including Kazakhstan and Mongolia). The RNA sequencing reads were aligned with all of the transcripts from whole genome annotation and Iso-Seq using *Bowtie2*^133^ (version 2.3.4.3). The expression of each transcript was quantified as *TPM* using *RSEM*^134^ (version 1.3.1). The DETs were detected using *edgeR*^145^ (version 3.32.0) based on an exact test and *P*-values were adjusted by the FDR method. A transcript was identified as a DET when the fold change was larger than 1 (or less than 1) and *q*-value was required less than 0.05.

### Hemoglobin concentration measurements

Blood samples were extracted from eight MN and six QTP chicks aged 4–6 weeks old during our field surveys in Mongolia and Qinghai-Tibet Plateau in 2017 and 2022. The hemoglobin concentration was measured using an automated Auto Hematology Analyzer BC-2600Vet^72^ (Mindray). Each sample was measured for three repeats.

### Plumage color measurements

We used an HR2000CG-UV-NIR spectrometer^146^ (Ocean Optics) with an HL 10000-Mini halogen lamp (Oceanhood) and a QR400-7-VIS-NIR fiber probe (Ocean Optics) to measure the plumage coloration. The spectra acquisition software package *OceanView* (version 1.6.7) was applied with the parameters set as: (1) integration time 100 ms; (2) the average number of spectra 5; (3) the electric dark correction on. The color module was selected and the CIELAB color space (L*a*b*) values were used to describe the plumage color. L* represents the lightness from black (0) to white (100), a* represents color from green (−128) to red (+127), and b* represents color from blue (−128) to yellow (+127).

For the comparison between MN and QTP saker populations, we randomly scanned the coloration of dorsal, wing and tail feathers (excluding spots/bands) of 11 chicks at 5–7 weeks old from each population during our field surveys in Mongolia and Qinghai-Tibet Plateau in 2019. Each feather was measured five times. The plumage color of each individual was assessed by the values of L*, a* and b*. The averaged L*, a* and b* values for each individual were plotted by *scatterplot3d* package and the PCA was conducted by *FactoMineR* and *factoextra* packages in *R* (version 4.0.3).

### Statistical analysis

All *P* values were calculated from Student’s *t* tests (two-sided) unless specified. For the *t* test, Cohen’s *d* is determined by calculating the mean difference between two groups, and dividing the result by the pooled standard deviation.

### Reporting summary

Further information on research design is available in the Nature Research Reporting Summary linked to this article.

## Supplementary information

Supplementary Information

Description of Additional Supplementary Files

Supplementary Data 1

Supplementary Data 2

Supplementary Data 3

Supplementary Data 4

Reporting Summary

## Data Availability

The saker genome assembly sequences have been deposited in the CNCB database under accession code GWHBOUP00000000. The PacBio, Bionano, HiSeq, and Hi-C data for genome assembly; whole genome resequencing data of 30 saker falcons and 10 gyrfalcons; and functional genomics data of ATAC-seq, Hi-C, Iso-Seq have been deposited in the CNCB database under accession code PRJCA010321. The four bird genomes for chromosomal alignments used in this study are available in the NCBI database under accession codes GCA_900496995.4, GCA_001887755.1, GCA_016699485.1, GCA_016128335.1, respectively. The RNA-seq data of saker blood samples used in this study are available in the CNCB database under accession code PRJCA008052. The RNA-seq data of chicken blood and marrow samples used in this study are available in the NCBI database under accession codes PRJNA542984, PRJEB44038, PRJNA323973, PRJNA279487, PRJNA412404, respectively. The ATAC-seq data of chicken bone and muscle samples used in this study are available in the NCBI database under accession code PRJNA433154. The H3K27ac ChIP-seq data of chicken leg scale skin samples used in this study are available in the NCBI database under accession code PRJNA561632. The chicken genome for reference used in this study are available in the NCBI database under accession code GCA_000002315.2 [https://www.ncbi.nlm.nih.gov/assembly/GCF_000002315.3]. Source data are provided with this paper.

## Source data

Source Data
