# Author Correction: Arctic introgression and chromatin regulation facilitated rapid Qinghai-Tibet Plateau colonization by an avian predator

**DOI:** 10.1038/s41467-022-35205-5

**Published:** 2022-12-01

**Authors:** Li Hu, Juan Long, Yi Lin, Zhongru Gu, Han Su, Xuemin Dong, Zhenzhen Lin, Qian Xiao, Nyambayar Batbayar, Batbayar Bold, Lucia Deutschová, Sergey Ganusevich, Vasiliy Sokolov, Aleksandr Sokolov, Hardip R. Patel, Paul D. Waters, Jennifer Ann Marshall Graves, Andrew Dixon, Shengkai Pan, Xiangjiang Zhan

**Affiliations:** 1 Key Laboratory of Animal Ecology and Conservation Biology, Institute of Zoology, Chinese Academy of Sciences, 100101 Beijing, China; 2 Cardiff University - Institute of Zoology Joint Laboratory for Biocomplexity Research, Chinese Academy of Sciences, 100101 Beijing, China; 3 University of the Chinese Academy of Sciences, 100049 Beijing, China; 4 Ministry of Education Key Laboratory for Biodiversity Science and Ecological Engineering, College of Life Sciences, Beijing Normal University, 100875 Beijing, China; 5 Wildlife Science and Conservation Center, Union Building B-802, Ulaanbaatar, 14210 Mongolia; 6 Raptor Protection of Slovakia, Trhová 54, SK-841 01, Bratislava, Slovakia; 7 Wild Animal Rescue Centre, Krasnostudencheskiy pr., 21-45, Moscow, 125422 Russia; 8 Institute of Plant and Animal Ecology, Ural Division Russian Academy of Sciences, 202-8 Marta Street, Ekaterinburg, 620144 Russia; 9 Arctic Research Station of the Institute of Plant and Animal Ecology, Ural Division Russian Academy of Sciences, 21 Zelenaya Gorka, Labytnangi, Yamalo-Nenetski District 629400 Russia; 10 The John Curtin School of Medical Research, Australian National University, Canberra, ACT 2601 Australia; 11 School of Biotechnology and Biomolecular Science, Faculty of Science, UNSW Sydney, Sydney, NSW 2052 Australia; 12 School of Life Sciences, La Trobe University, Melbourne, VIC Australia; 13 Emirates Falconers’ Club, Al Mamoura Building (A), P.O. Box 47716, Muroor Road, Abu Dhabi, UAE; 14 International Wildlife Consultants, P.O. Box 19, Carmarthen, SA33 5YL UK; 15 Center for Excellence in Animal Evolution and Genetics, Chinese Academy of Sciences, Kunming, 650223 China

**Keywords:** Evolutionary genetics, Gene regulation, Genome evolution, Genetic hybridization

Correction to: *Nature Communications* 10.1038/s41467-022-34138-3 published online 27 October 2022

The original version of this Article contained an error in the Results subsection “Hybridization with Arctic gyrfalcons facilitated sakers’ adaptation to cold extremes”, which incorrectly read ‘To do this, we applied an ABBA-BABA model^37^, and found five outstanding genomic islands (>200 Kb) on the five chromosomes (Chr 1, 3, 5, 8 and 16) exhibiting adaptive introgression signatures (top 1% D = 0.73, top 1% f_d_ = 0.70) (Fig. 2b; Supplementary Fig. 10) which were much longer than the expected length of fragments (26.6 Kb) from incomplete lineage sorting (ILS; “Methods”).’ The correct version states ‘26.0 Kb’ in place of ‘26.6 Kb’.

The original version of this Article contained an error in the Methods subsection “De novo assembly of the saker falcon genome”, which incorrectly read ‘We assembled a chromosome-level reference genome of an adult female saker falcon (aQTP saker rescued by XiningWildlife Park) (2n = 56) using a multi-platform sequencing strategy (PacBio, Illumina, Bionano)’. The correct version states ‘2n = 52’ in place of ‘2n = 56’.

The original version of this Article contained an error in the Methods subsection “Estimation of adaptively introgressed signals and discrimination from ILS”, which incorrectly read ‘In our case, we set the parameters according to the *fastsimcoal2* result: *t* = 3181 generations (21 ka), *m* = 0.23 (the minimum introgression rate in *Frappe* result), r = 2E−08 (assuming that each of the 26 chromosomes experiences on average one crossover per generation^123^). When the *P* was larger than 0.05, the fragments shorter than 26.6 Kb were considered as those that may be influenced by ILS. Accordingly, fragments that longer than 26.6 Kb were considered from introgression.’ The correct version states ‘*m* = 0.213’ in place of ‘*m* = 0.23’, ‘fragments shorter than 26.0 Kb’ in place of ‘fragments shorter than 26.6 Kb’, and ‘fragments longer than 26.0 Kb’ in place of ‘fragments that longer than 26.6 Kb’.

These have been corrected in both the PDF and HTML versions of the Article.

