# Supplementary material for: Arctic introgression and chromatin regulation facilitated rapid Qinghai-Tibet Plateau colonization by an avian predator
Source: Nat Commun. 2022 Oct 27;13:6413. doi: 10.1038/s41467-022-34138-3 (PMC9613686; doi:10.1038/s41467-022-34138-3)
Supplement: Supplementary file 1 — Supplementary Information [file 41467_2022_34138_MOESM1_ESM.pdf]

Supplementary Information for Arctic introgression and chromatin regulation facilitated rapid Qinghai-Tibet Plateau colonization by an avian predator

Li Hu[1,2,3†], Juan Long[1,2,3†], Yi Lin[1,2,3†], Zhongru Gu[1,2], Han Su[1,2,3], Xuemin Dong[1,3], Zhenzhen Lin[1,2], Qian Xiao[1,3,4], Nyambayar Batbayar[5], Bold Batbayar[1,3,5], Lucia Deutschova[6], Sergey Ganusevich[7], Vasiliy Sokolov[8], Aleksandr Sokolov[9], Hardip R. Patel[10], Paul D. Waters[11], Jennifer Ann Marshall Graves[12], Andrew Dixon[13,14], Shengkai Pan[1,2*], Xiangjiang Zhan[1,2,3,15*]

1. Key Laboratory of Animal Ecology and Conservation Biology, Institute of Zoology, Chinese Academy of Sciences, Beijing 100101, China.
2. Cardiff University - Institute of Zoology Joint Laboratory for Biocomplexity Research, Chinese Academy of Sciences, Beijing 100101, China.
3. University of the Chinese Academy of Sciences, Beijing 100049, China.
4. Ministry of Education Key Laboratory for Biodiversity Science and Ecological Engineering, College of Life Sciences, Beijing Normal University, Beijing 100875, China.
5. Wildlife Science and Conservation Center, Union Building B-802, Ulaanbaatar 14210, Mongolia.
6. Raptor Protection of Slovakia, Trhová 54, SK-841 01 Bratislava 42, Slovakia.
7. Wild Animal Rescue Centre, Krasnostudencheskiy pr., 21-45, Moscow 125422, Russia.
8. Institute of Plant and Animal Ecology, Ural Division Russian Academy of Sciences, 202-8 Marta Street, Ekaterinburg 620144, Russia.
9. Arctic Research Station of the Institute of Plant and Animal Ecology, Ural Division Russian Academy of Sciences, 21 Zelenaya Gorka, Labytnangi, Yamalo-Nenetski District 629400, Russia.
10. The John Curtin School of Medical Research, Australian National University, Canberra, ACT 2601, Australia.
11. School of Biotechnology and Biomolecular Science, Faculty of Science, UNSW Sydney, Sydney, NSW 2052, Australia.
12. School of Life Sciences, La Trobe University, Melbourne, Australia.

13. Emirates Falconers' Club, P.O. Box 47716, Al Mamoura Building (A), Muroor Road, Abu Dhabi, UAE.
14. International Wildlife Consultants, P.O. Box 19, Carmarthen, SA33 5YL, UK.
15. Center for Excellence in Animal Evolution and Genetics, Chinese Academy of Sciences, Kunming 650223, China.

†These authors contributed equally: Li Hu, Juan Long, Yi Lin.

*Corresponding authors:
Shengkai Pan
E-mail: pansk@ioz.ac.cn
Xiangjiang Zhan
E-mail: zhanxj@ioz.ac.cn

Supplementary Figures

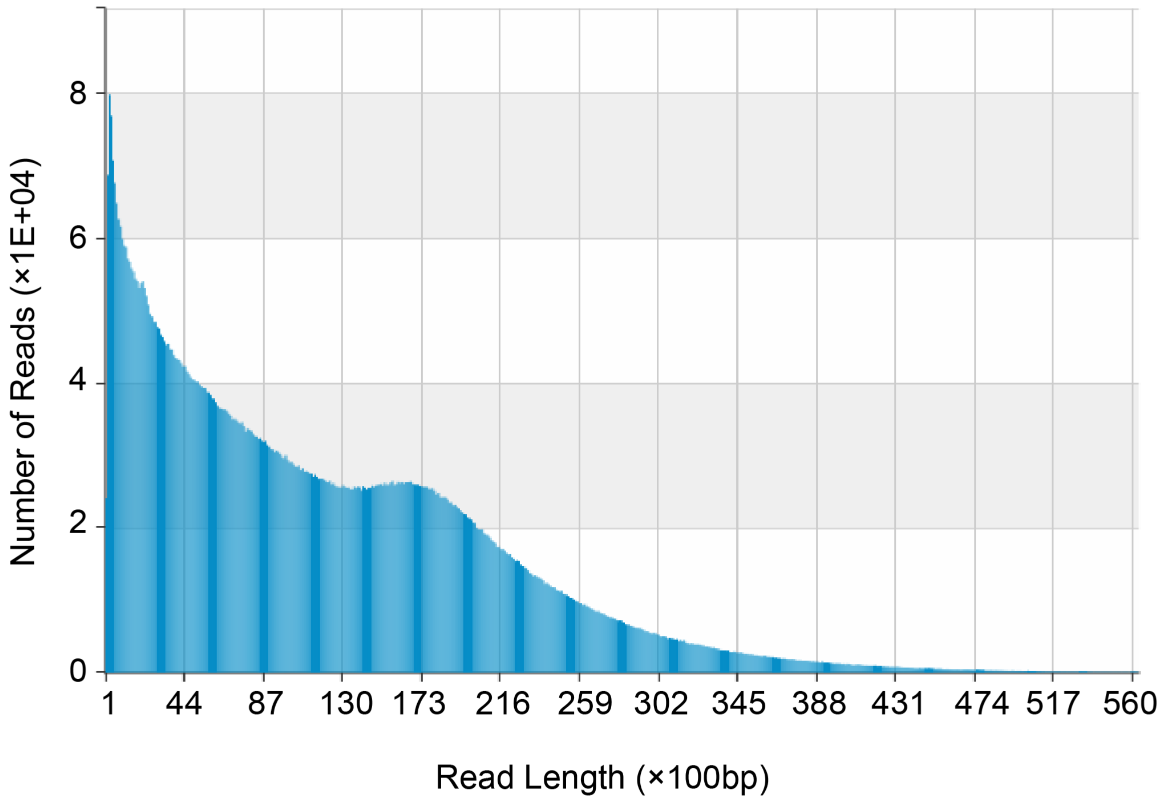

Supplementary Fig. 1. The length distribution of subreads generated by the PacBio sequencing of the saker falcon genome.

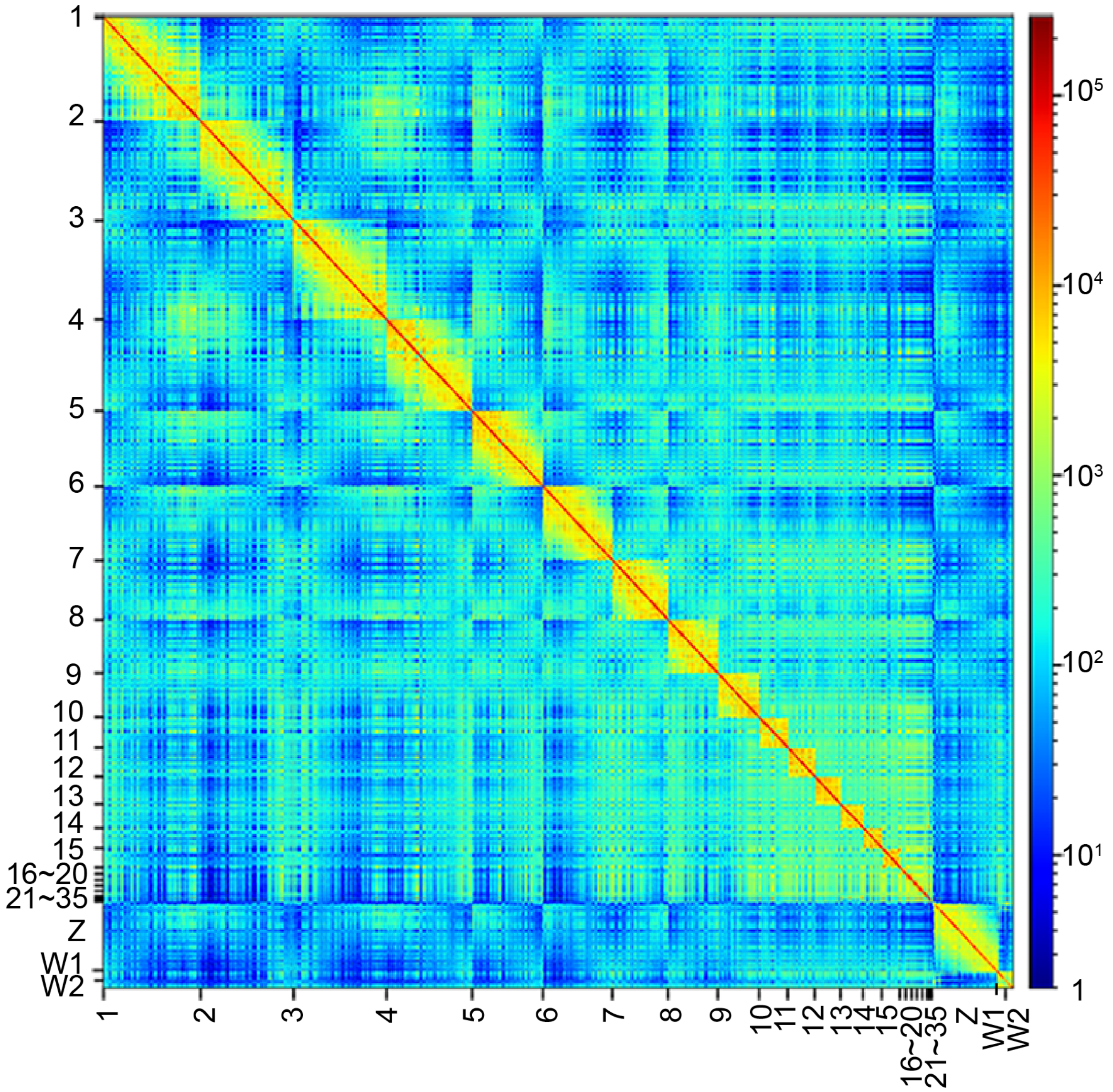

Supplementary Fig. 2. The Hi-C contact map of the assembled super-scaffolds of saker falcon (bin size = 1 Mb). The bar shows the contact frequency between each of the two bins from low (blue) to high (red).

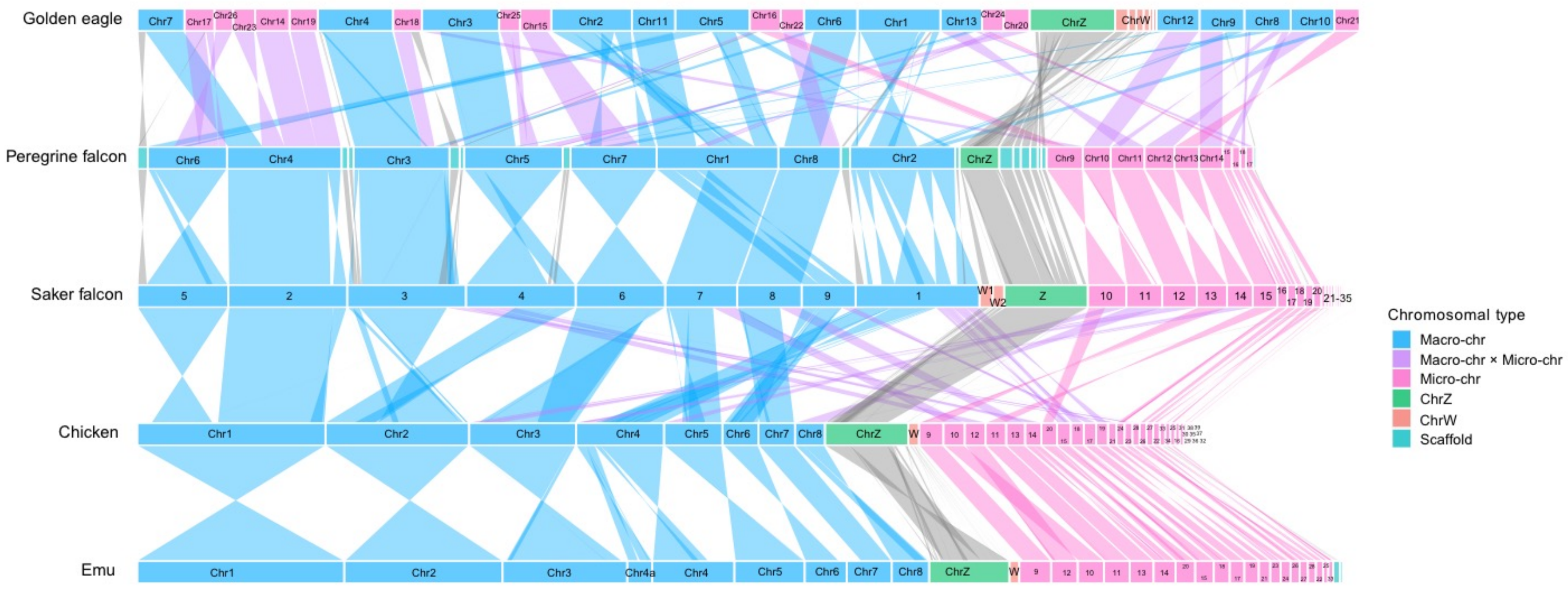

Supplementary Fig. 3. The alignments between super-scaffolds of sakers assembled by Hi-C and chromosomes of other avian species. The blue, pink, green, orange and sky blue blocks show the macro-, micro-, *Z*-, W- chromosomes and unanchored scaffolds respectively. The violet blocks show alignments between macro- and micro- chromosomes in different species.

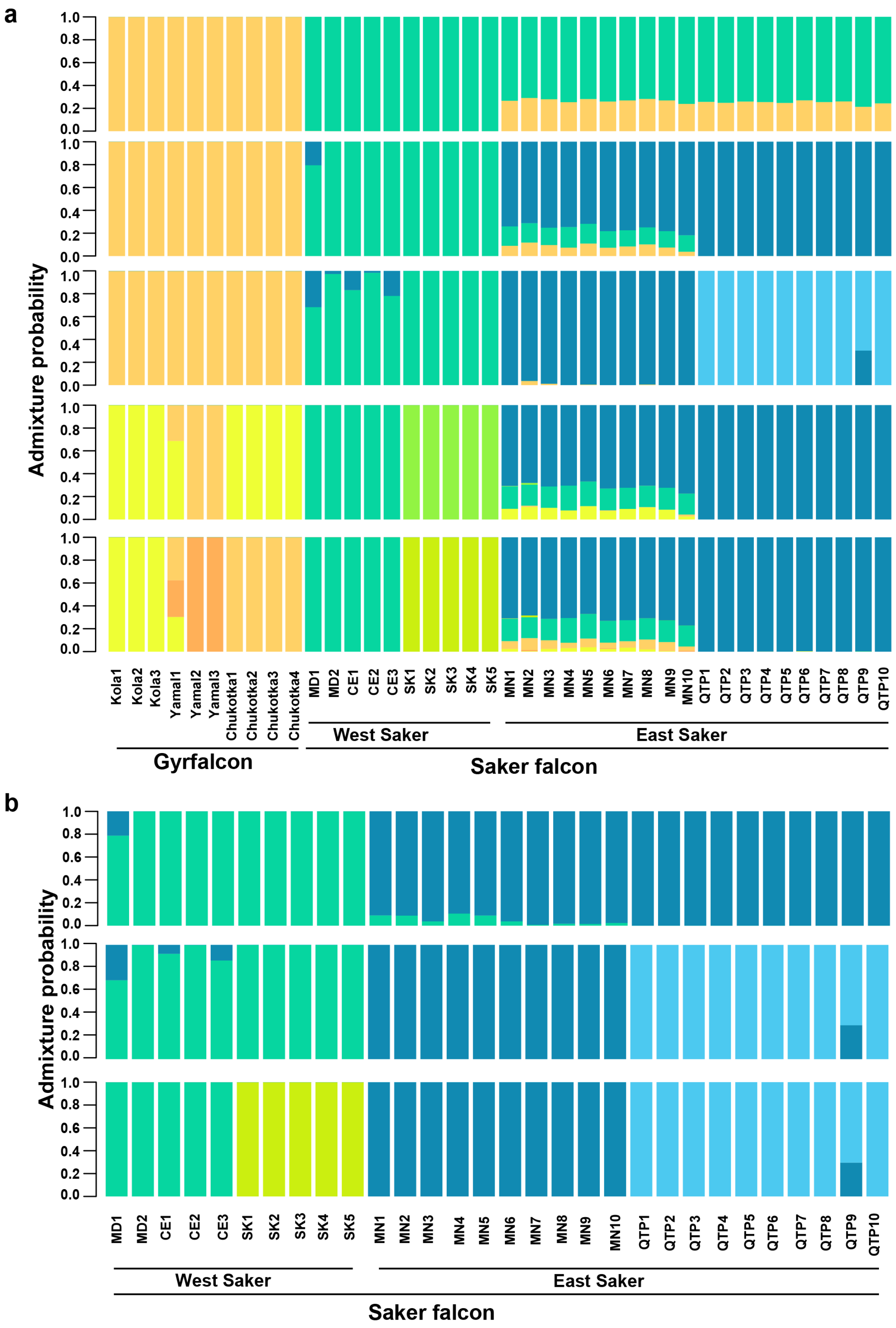
a
b
Admixture probability
1.0
0.8
0.6
0.4
0.2
0.0
Kola1
Kola2
Kola3
Yamal1
Yamal2
Yamal3
Chukotka1
Chukotka2
Chukotka3
Chukotka4
MD1
MD2
CE1
CE2
CE3
SK1
SK2
SK3
SK4
SK5
MN1
MN2
MN3
MN4
MN5
MN6
MN7
MN8
MN9
MN10
QTP1
QTP2
QTP3
QTP4
QTP5
QTP6
QTP7
QTP8
QTP9
QTP10
West Saker
East Saker
Gyrfalcon
Saker falcon

c

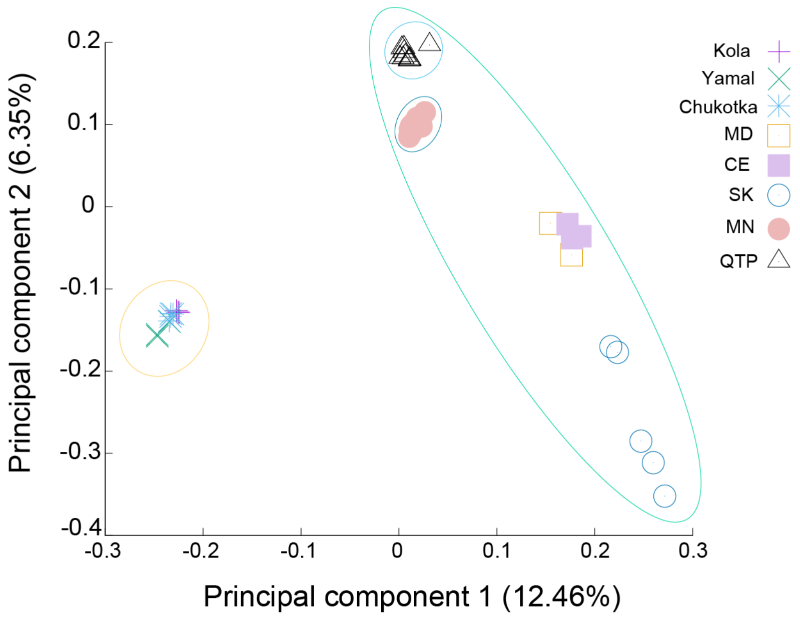

Supplementary Fig. 4. Population genetic structure. a The *Frappe* results with autosomal SNPs identified from the studied gyrfalcons and sakers (K = 2-6), and **b** from sakers only (K = 2-4). **c** PCA results with autosomal SNPs. Wild sakers were sampled from Moldova (MD), Crimea (CE), Slovakia (SK), Mongolia (MN) and Qinghai-Tibet Plateau (QTP). Gyrfalcon samples were collected from Kola, Yamal and Chukotka in Russia.

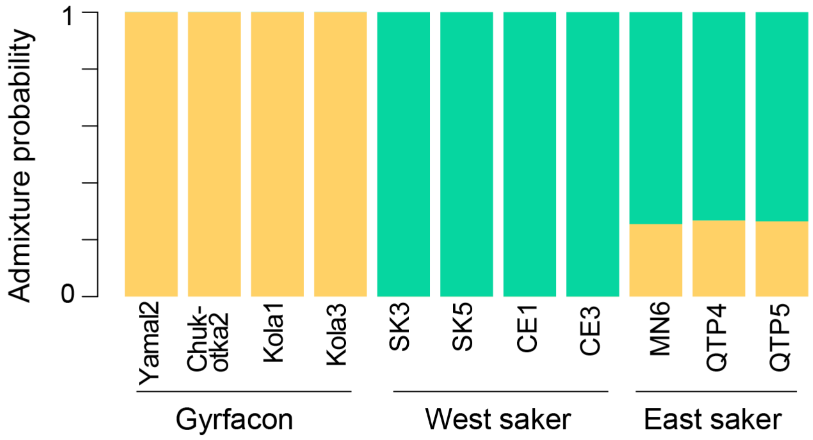

Supplementary Fig. 5. The *Frappe* result of SNPs on Z chromosomes in male falcons (*K* = 2). Wild saker samples were collected from Crimea (CE), Slovakia (SK), Mongolia (MN) and Qinghai-Tibet Plateau (QTP). Gyrfalcon samples were collected from Kola, Yamal and Chukotka.

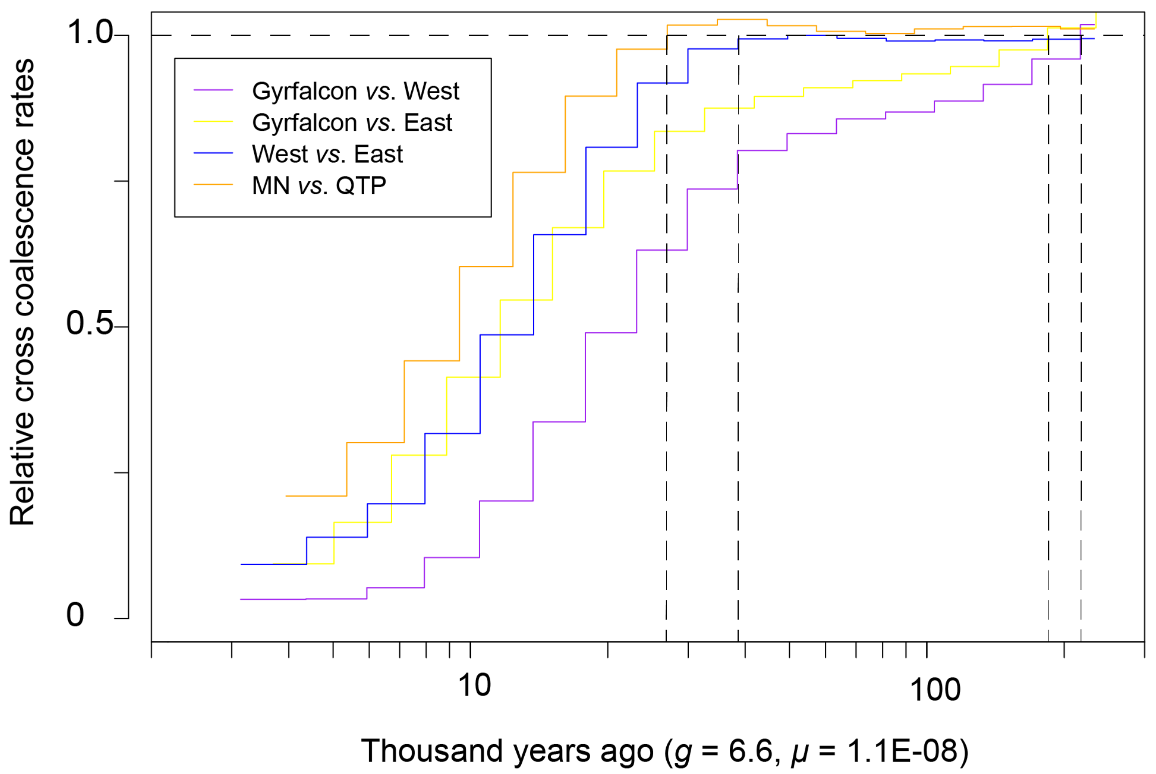

Supplementary Fig. 6. Pairwise comparisons of relative cross coalescence rates using *MSMC*. *g* (generation time) is 6.6 years, and μ (mutation rate per generation) is 1.1E-08. The relative cross coalescence rate is close to one when the two populations are not divergent.

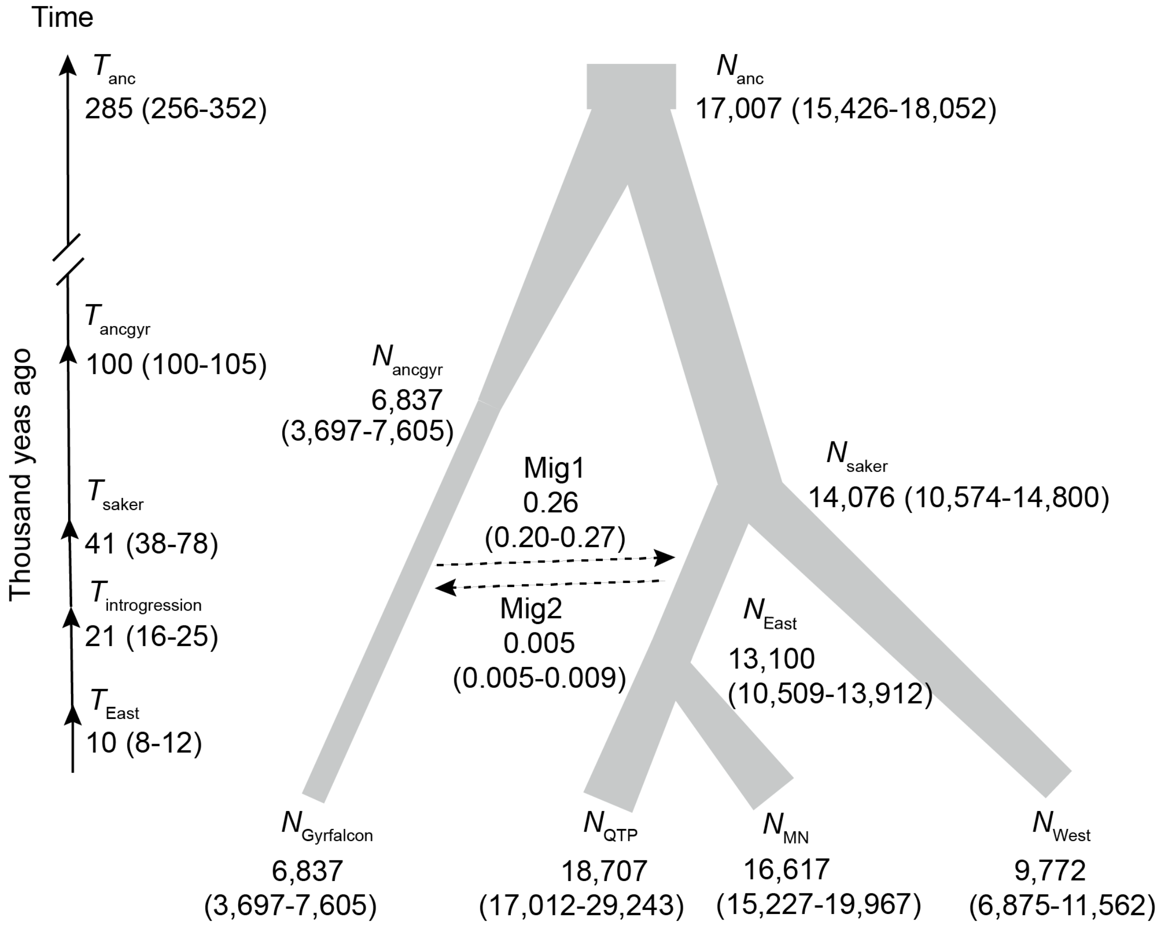

Supplementary Fig. 7. Demographic simulation of saker falcons using *fastsimcoal2*. "Mig" means the estimated introgression rate. "*T*" shows the estimated divergence or hybridization time. "*N*" shows the estimated effective population size. "anc" means the ancestral.

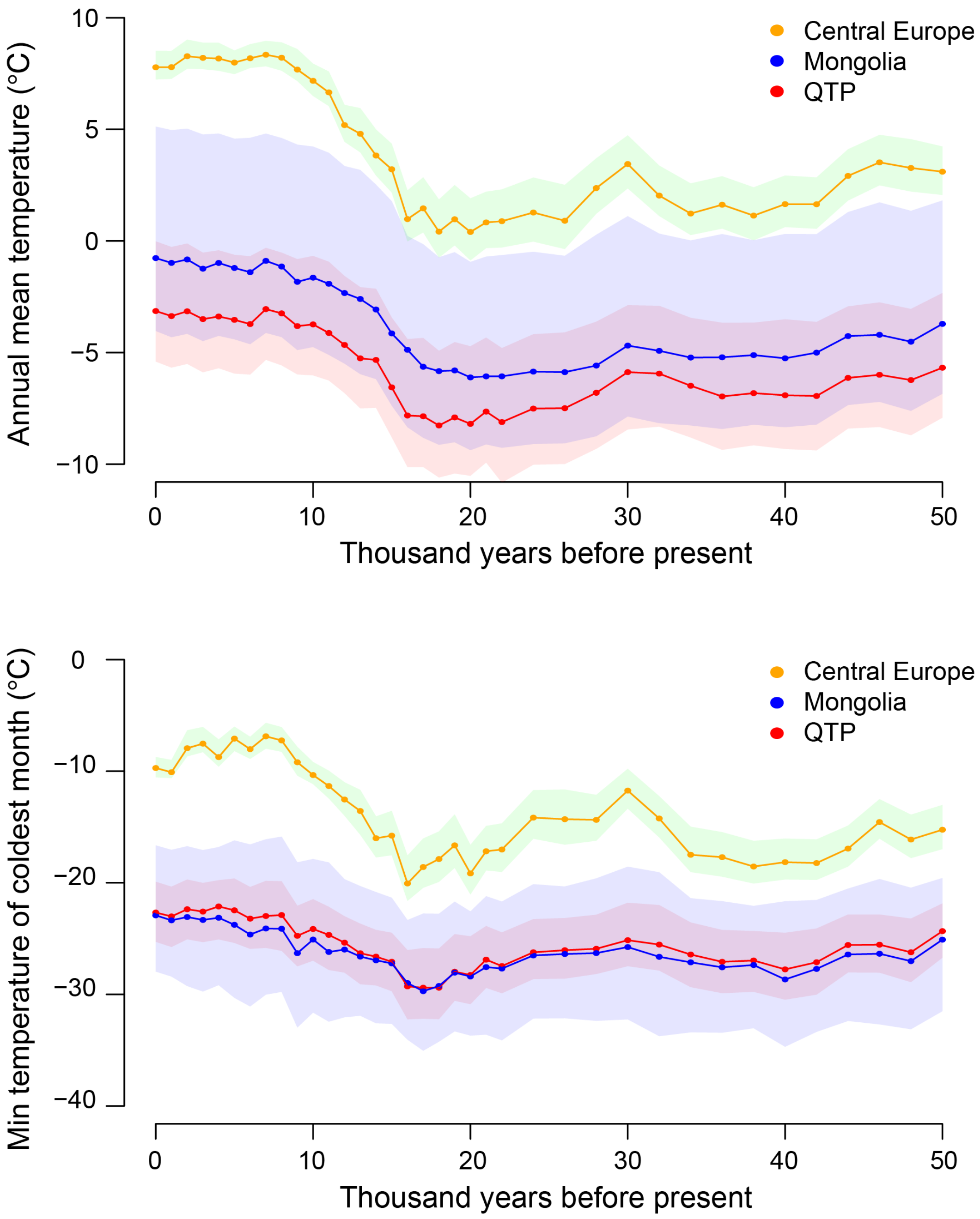

Supplementary Fig. 8. The temperature (annual and minimum of coldest months) in Central Europe, Mongolia and Qinghai-Tibet Plateau (QTP) from *ca*. 50 ka to present. The mean and CI (25%-75%) are shown.

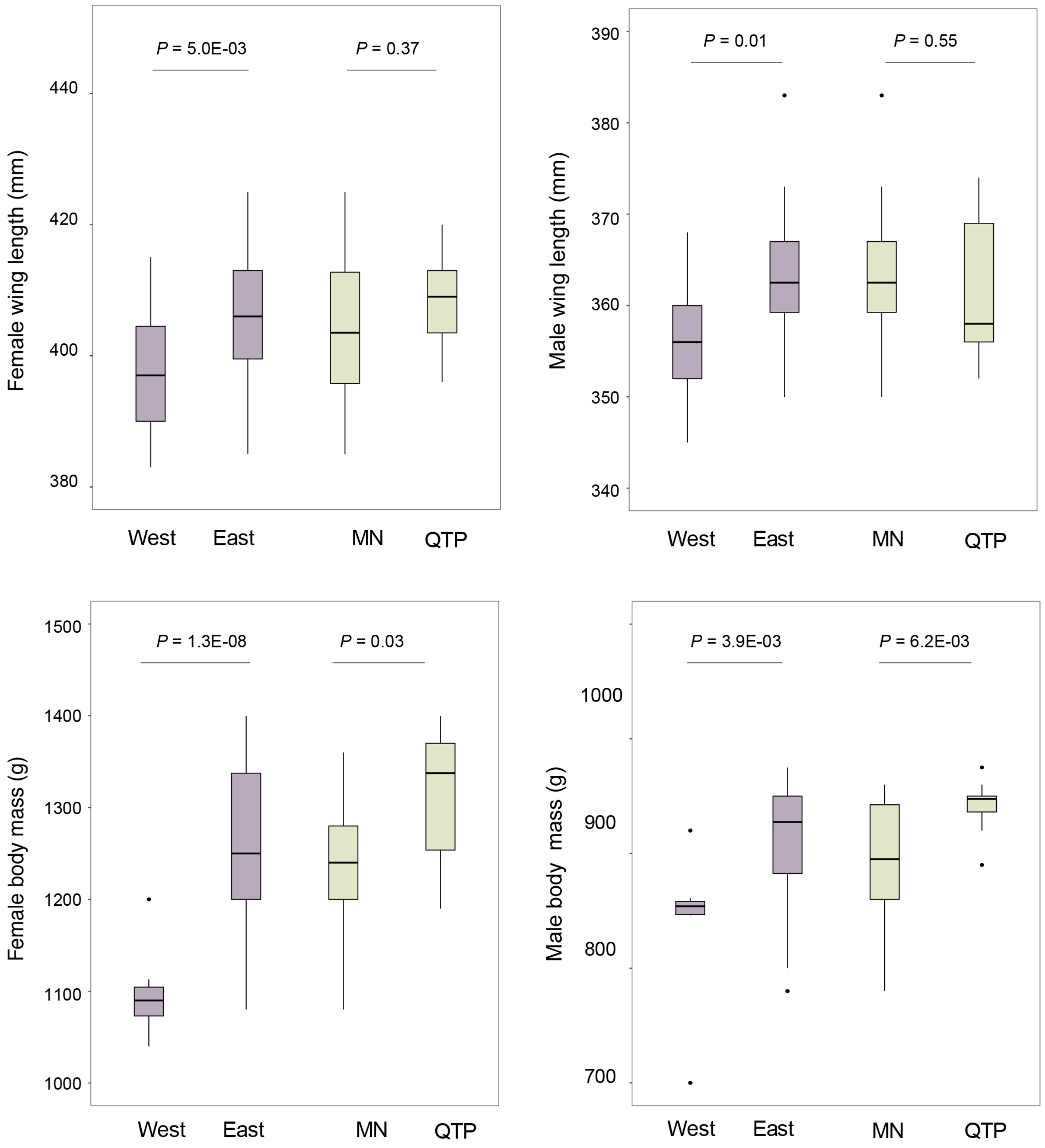

Supplementary Fig. 9. Comparisons of wing length and body mass in female and male sakers between West and East, MN and QTP populations. For wing length comparisons, 19 females and 17 males were from West population[1,2] , and 31 females and 26 males from East population (MN: 20 females and 18 males[3]; QTP: 11 females and eight males). For body mass comparisons, 11 females and eight males were from West population[4], and 23 females and 26 males from East population (MN: 15 females and 16 males from MN[3]; QTP: eight females and ten males). In the box plots, the centre line

represents the median, whiskers represent maximum and minimum values, and box boundaries represent 75th and 25th percentiles. A two-sided *t*-test was used. Source data are provided as a Source Data file.

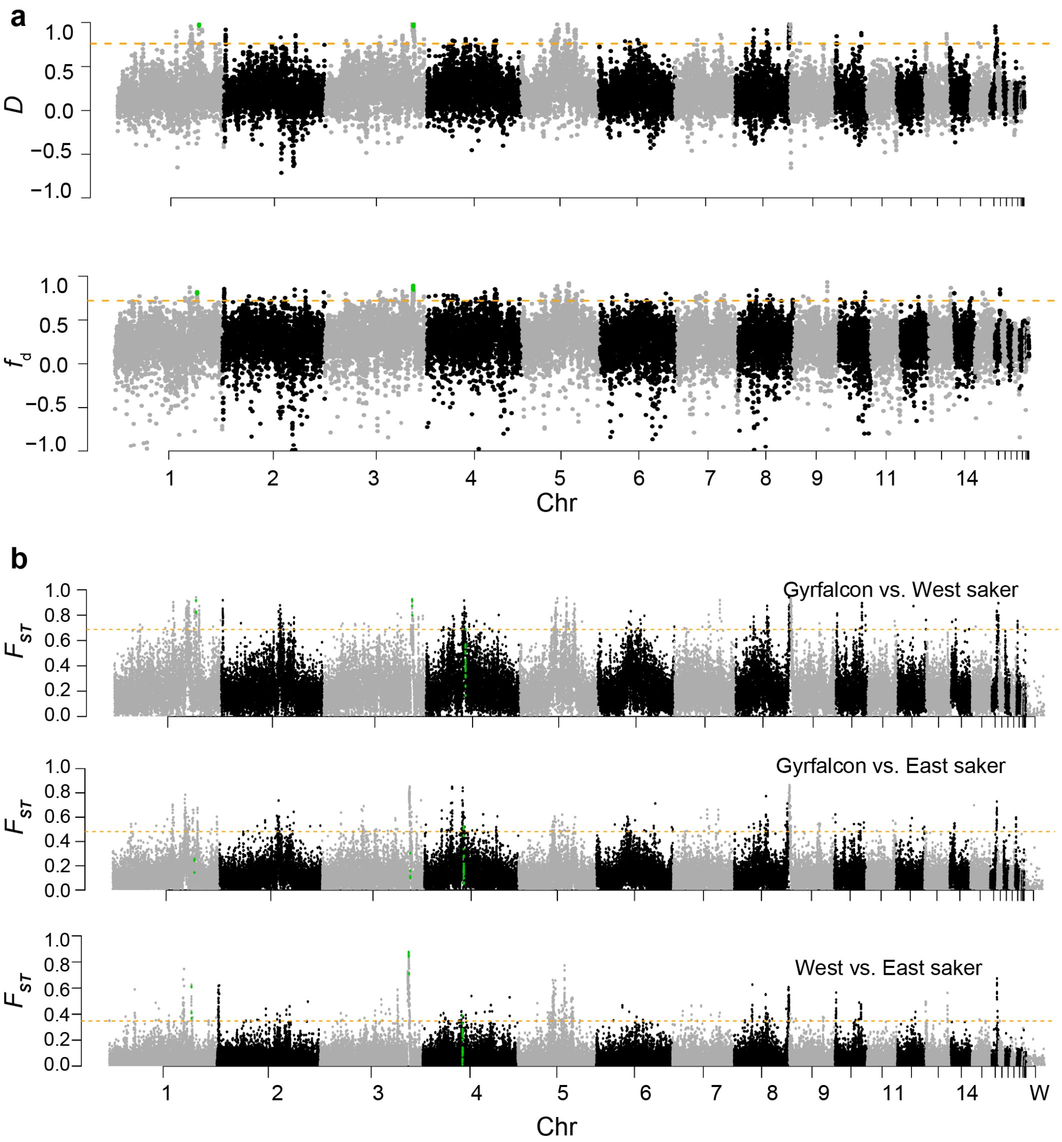

Supplementary Fig. 10. Identification of adaptively introgressed fragments. a The genome-wide distribution of *D* and f_d values based on ABBA-BABA modelling from the (((West saker, East saker), gyrfalcon), peregrine) topology in a window size of 100 Kb with a step size of 50 Kb. The orange lines represent the top 1% cut-off value (0.73 and 0.70, respectively). **b** The genome-wide distribution of F_{ST} values in three comparisons (Gyrfalcon/West saker, Gyrfalcon/East saker and West/East saker) in a sliding window size of 20 Kb. The orange lines represent the top 1% cut-off value (0.69, 0.48, 0.35 respectively). The green dots represent the windows covering introgressed *SCARB1* (Chr 1) and *SCMH1* (Chr 3) genes. In addition, the 500 Kb hard sweep (Chr 4) selected in

QTP sakers (**Supplementary Fig. 22**) but not selected in other falcons are also shown in green dots.

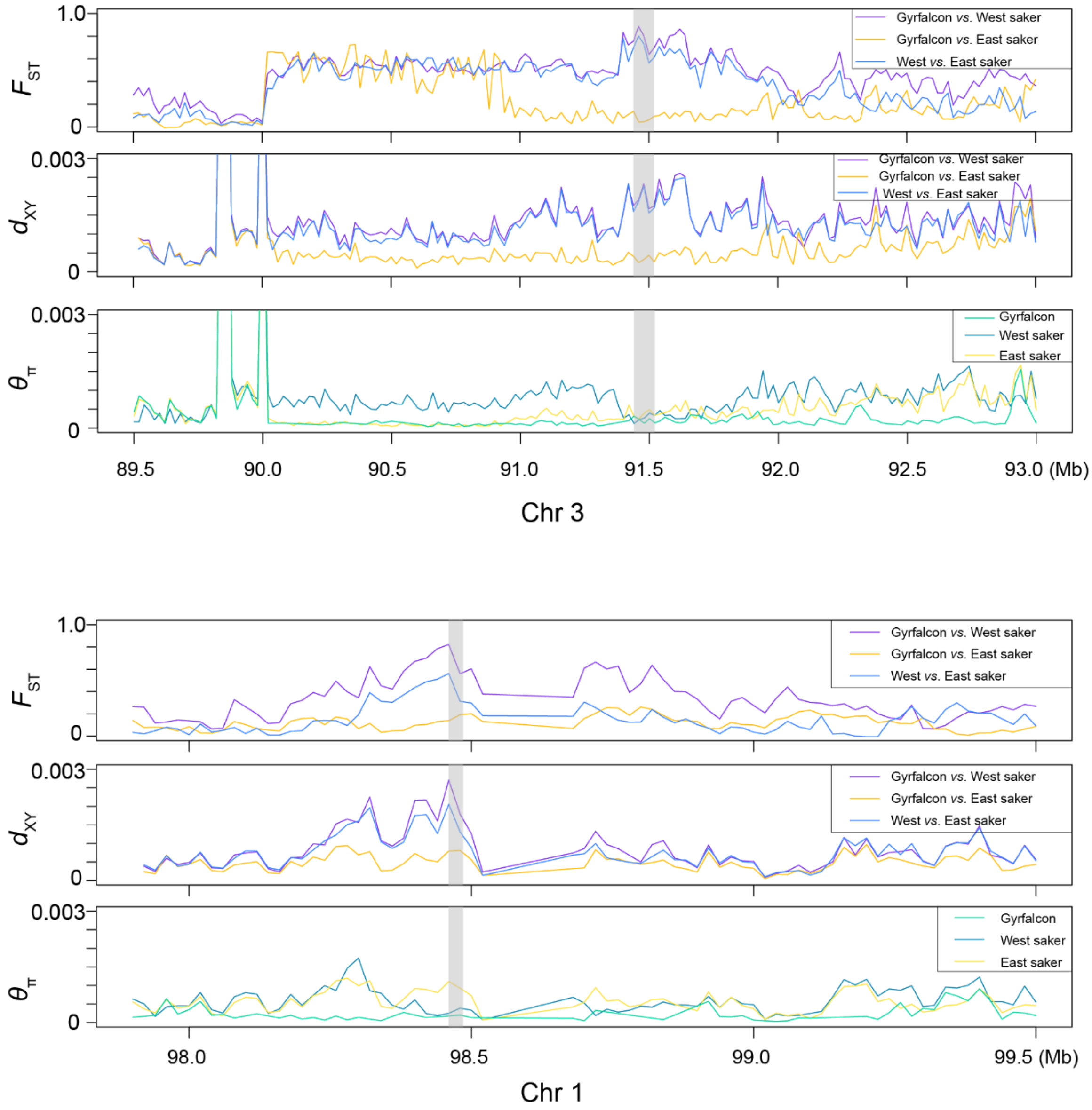

Supplementary Fig. 11. Population differentiation indexes (F_{ST}, d_{XY}) and genetic diversity (θ_π) of the introgressed genomic islands in three comparisons (Gyrfalcon/West saker, Gyrfalcon/East saker and West/East saker) on Chr 1 and Chr 3. The grey blocks show the windows covering *SCARB1* (Chr 1) and *SCMH1* (Chr 3) genes.

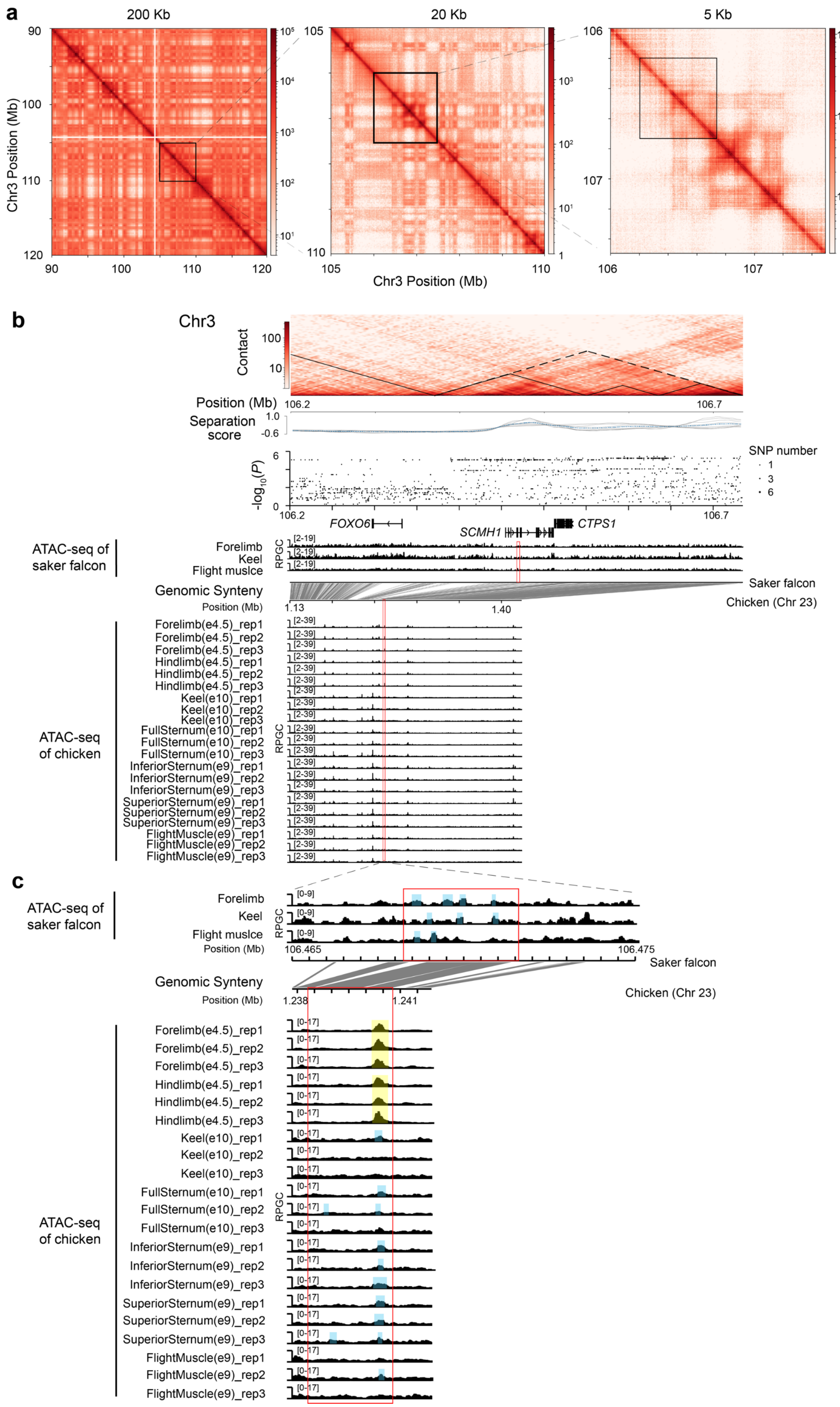
a
200 Kb
20 Kb
5 Kb
Chr3 Position (Mb)
Chr3 Position (Mb)
b
Chr3
Contact
Position (Mb)
Separation score
SNP number
FOXO6
SCMH1
CTPS1
ATAC-seq of saker falcon
Forelimb
Keel
Flight muslce
RPGC
Genomic Synteny
Position (Mb)
Saker falcon
Chicken (Chr 23)
ATAC-seq of chicken
Forelimb(e4.5)_rep1
Forelimb(e4.5)_rep2
Forelimb(e4.5)_rep3
Hindlimb(e4.5)_rep1
Hindlimb(e4.5)_rep2
Hindlimb(e4.5)_rep3
Keel(e10)_rep1
Keel(e10)_rep2
Keel(e10)_rep3
FullSternum(e10)_rep1
FullSternum(e10)_rep2
FullSternum(e10)_rep3
InferiorSternum(e9)_rep1
InferiorSternum(e9)_rep2
InferiorSternum(e9)_rep3
SuperiorSternum(e9)_rep1
SuperiorSternum(e9)_rep2
SuperiorSternum(e9)_rep3
FlightMuscle(e9)_rep1
FlightMuscle(e9)_rep2
FlightMuscle(e9)_rep3
c
ATAC-seq of saker falcon
Forelimb
Keel
Flight muslce
Position (Mb)
Saker falcon
Genomic Synteny
Position (Mb)
Chicken (Chr 23)
ATAC-seq of chicken

Supplementary Fig. 12. Identification of *cis*-regulatory elements (CREs) around *SCMH1* gene using Hi-C and ATAC-seq data. a The heatmaps show the Hi-C contact matrix (bin size = 200 Kb, 20 Kb, 5 Kb) around *FOXO6*/*SCMH1* gene block. **b** A zoom-in view of the black square in the heatmap of 5 Kb bin size of **a**. The solid and dash black triangles in the heatmap show the TAD structures (bin size = 5 Kb, 20 Kb respectively). The curves below the heatmap represent the TAD separation scores between the left and right regions at each bin with different window sizes (grey lines) and mean scores (blue lines). The dot plot shows the logarithmically transformed *P*-value (*hapFLK* test) for each SNP calculated between the West and East saker populations. The ATAC-seq tracks (normalized using reads per genome coverage (RPGC)) around the *SCMH1* gene were identified from saker and chicken embryonic tissues (data are available in the NCBI database under accession code PRJNA433154)[5], and CREs are indicated by peaks. The two red boxes denote a homologous fragment between the saker falcon and chicken, and the fragment in saker is used for the luciferase reporter assay in this study. The grey blocks show the syntenic regions of the focal genomic fragment between saker and chicken genomic sequences. The window size is 1 Kb. **c** The zoom-in view of the ATAC-seq tracks around the focal fragment for the luciferase experiment. The peaks in samples without biological replicates are denoted by sky-blue blocks and the ones in samples with at least two biological replicates are denoted by yellow blocks (irreproducibility discovery rate (IDR) $\leqslant$ 0.05). The window size is 50 bp.

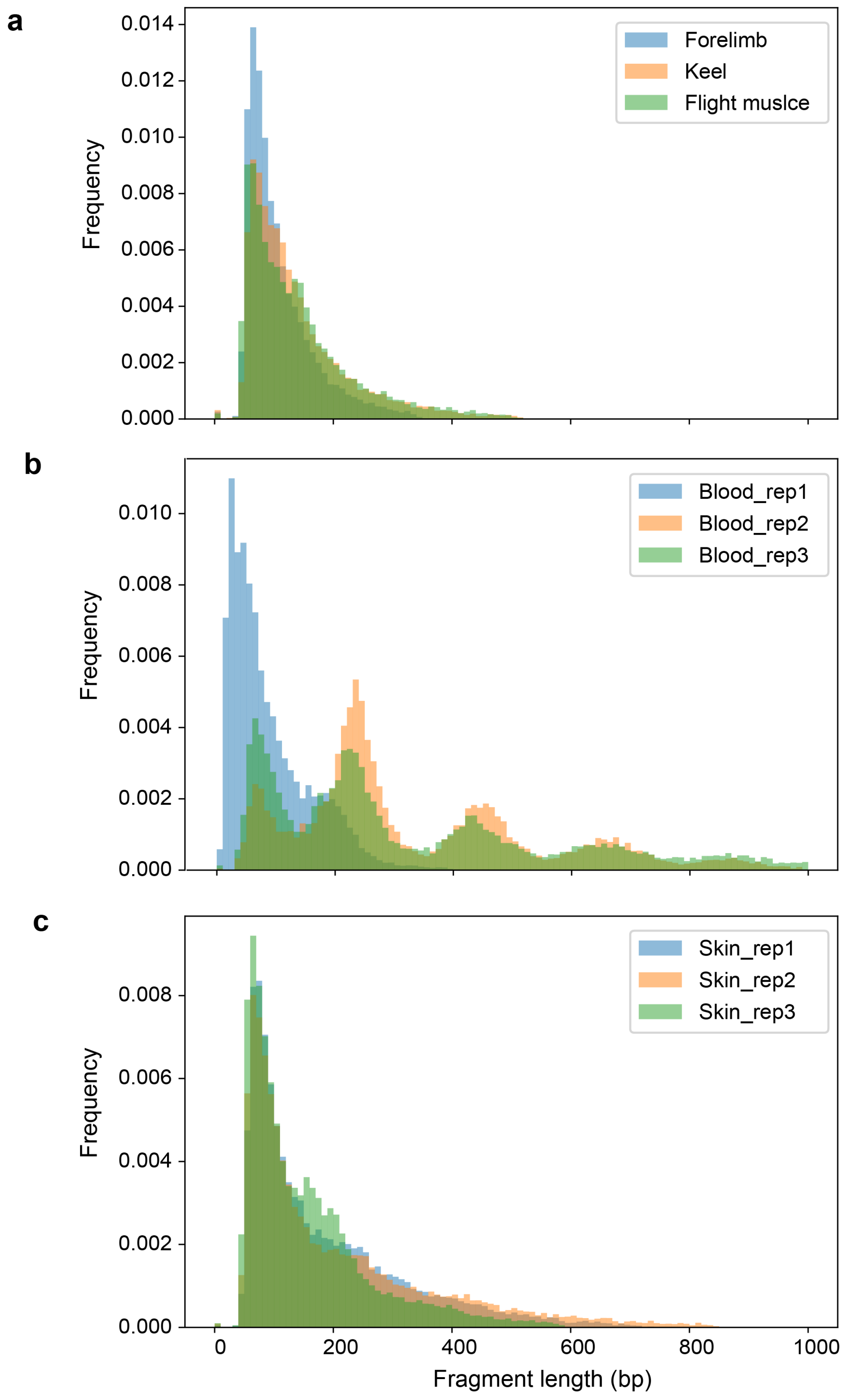

Supplementary Fig. 13. Fragment size distributions of ATAC-seq data from forelimb/keel/flight muscle of a QTP saker embryo (a), three biologically independent blood samples (b) and three biologically independent skin (c) samples of QTP sakers.

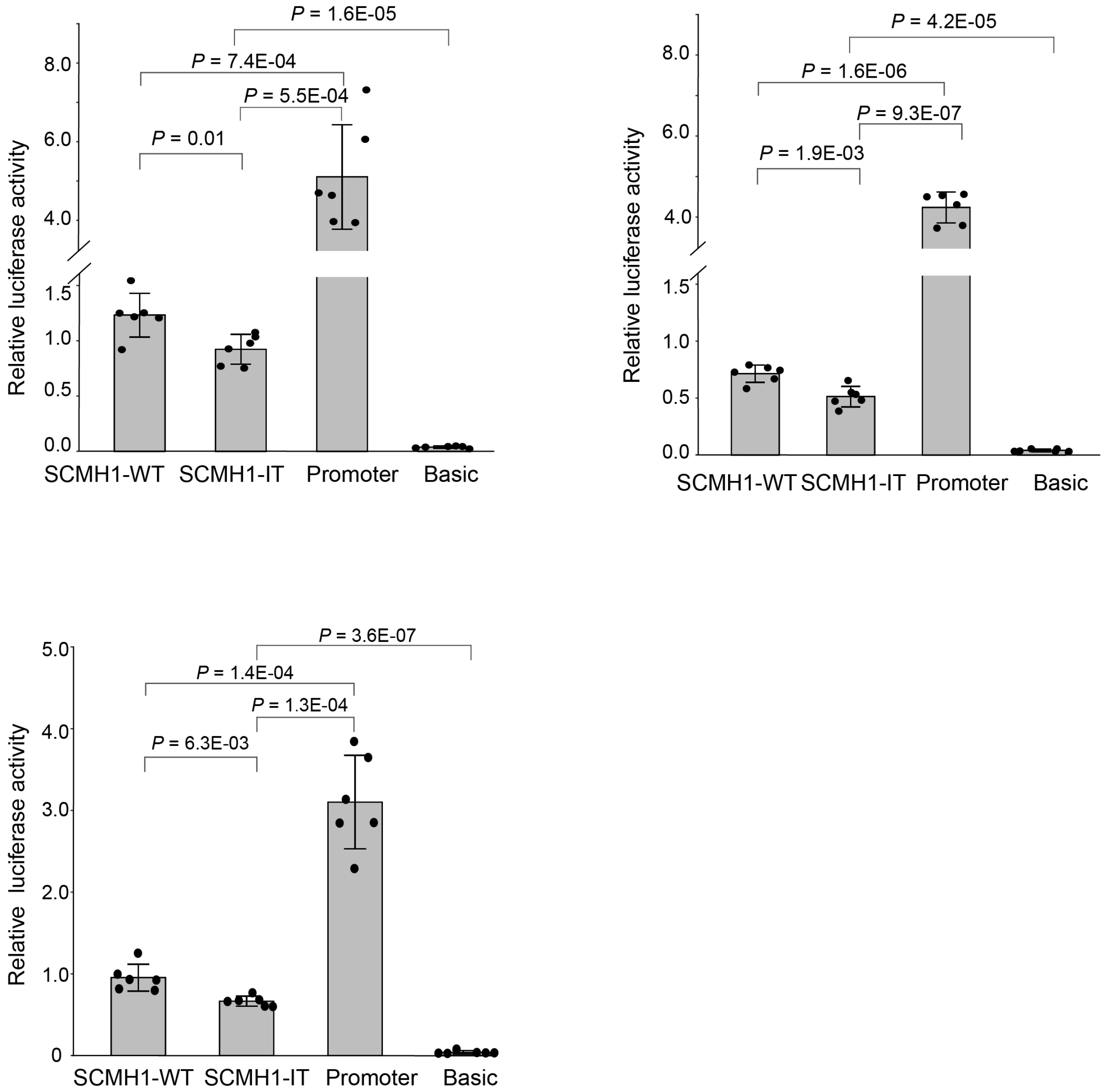

Supplementary Fig. 14. Three biologically independent replicates of luciferase reporter assays for the dominant wild type (WT) and introgressed type (IT) of the focal CRE in *SCMH1* gene. The SCMH1-IT and SCMH1-WT groups were cloned into pGL3-Promoter vectors. Promoter (pGL3-Promoter) and Basic (pGL3-Basic) groups were used as controls respectively. The bars display mean ± SD (*N* = 6 technical replicates). A two-sided *t*-test was used. Source data are provided as a Source Data file.

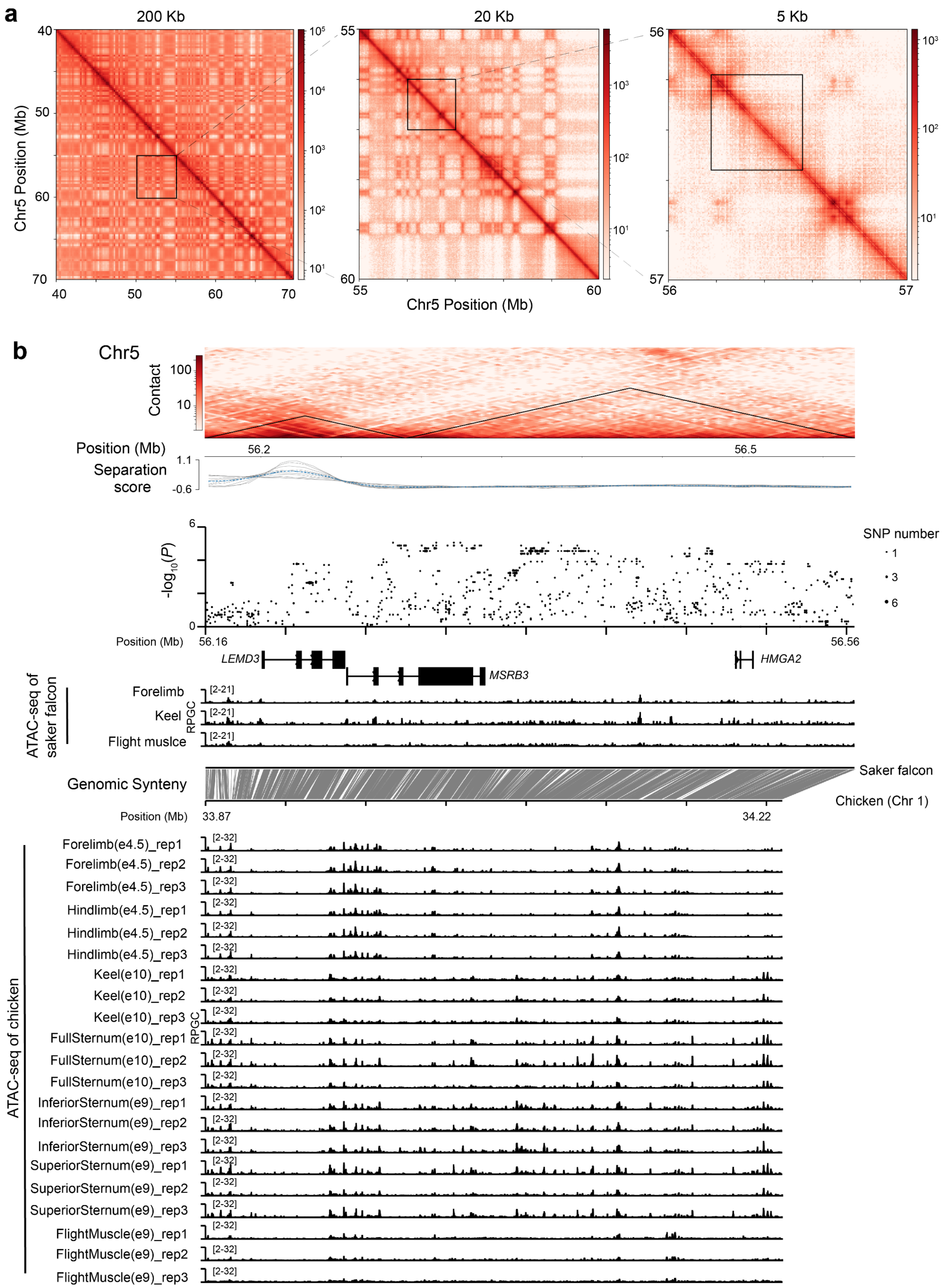

a
200 Kb
20 Kb
5 Kb
Chr5 Position (Mb)
Chr5 Position (Mb)
b
Chr5
Contact
Position (Mb)
56.2
56.5
Separation score
-log10(P)
SNP number
Position (Mb)
56.16
56.56
LEMD3
MSRB3
HMGA2
ATAC-seq of saker falcon
Forelimb
Keel
Flight muslce
RPGC
Genomic Synteny
Saker falcon
Chicken (Chr 1)
Position (Mb)
33.87
34.22
ATAC-seq of chicken
Forelimb(e4.5)_rep1
Forelimb(e4.5)_rep2
Forelimb(e4.5)_rep3
Hindlimb(e4.5)_rep1
Hindlimb(e4.5)_rep2
Hindlimb(e4.5)_rep3
Keel(e10)_rep1
Keel(e10)_rep2
Keel(e10)_rep3
FullSternum(e10)_rep1
FullSternum(e10)_rep2
FullSternum(e10)_rep3
InferiorSternum(e9)_rep1
InferiorSternum(e9)_rep2
InferiorSternum(e9)_rep3
SuperiorSternum(e9)_rep1
SuperiorSternum(e9)_rep2
SuperiorSternum(e9)_rep3
FlightMuscle(e9)_rep1
FlightMuscle(e9)_rep2
FlightMuscle(e9)_rep3

Supplementary Fig. 15. Identification of *cis*-regulatory elements (CREs) around *LEMD3/MSRB3/HMGA2* gene block using Hi-C and ATAC-seq data. a The heatmaps show the Hi-C contact matrix (bin size = 200 Kb, 20 Kb, 5 Kb) around *LEMD3/MSRB3/HMGA2* gene block. **b** A zoom-in view of the black square in the heatmap of 5 Kb bin size of **a**. The black triangles in the heatmap show the TAD structures (bin size = 5 Kb). The curves below the heatmap represent the TAD separation scores between the left and right regions at each bin with different window sizes (grey lines) and mean scores (blue lines). The dot plot shows the logarithmically transformed *P*-value (*hapFLK* test) for each SNP calculated between the West and East saker populations. The ATAC-seq tracks (normalized using RPGC) around the *LEMD3/MSRB3/HMGA2* gene block were identified from saker and chicken embryonic tissue samples (data are available in the NCBI database under accession code PRJNA433154)[5]. The grey blocks show the syntenic regions of the focal genomic fragment between saker and chicken genomic sequences. The window size is 1 Kb.

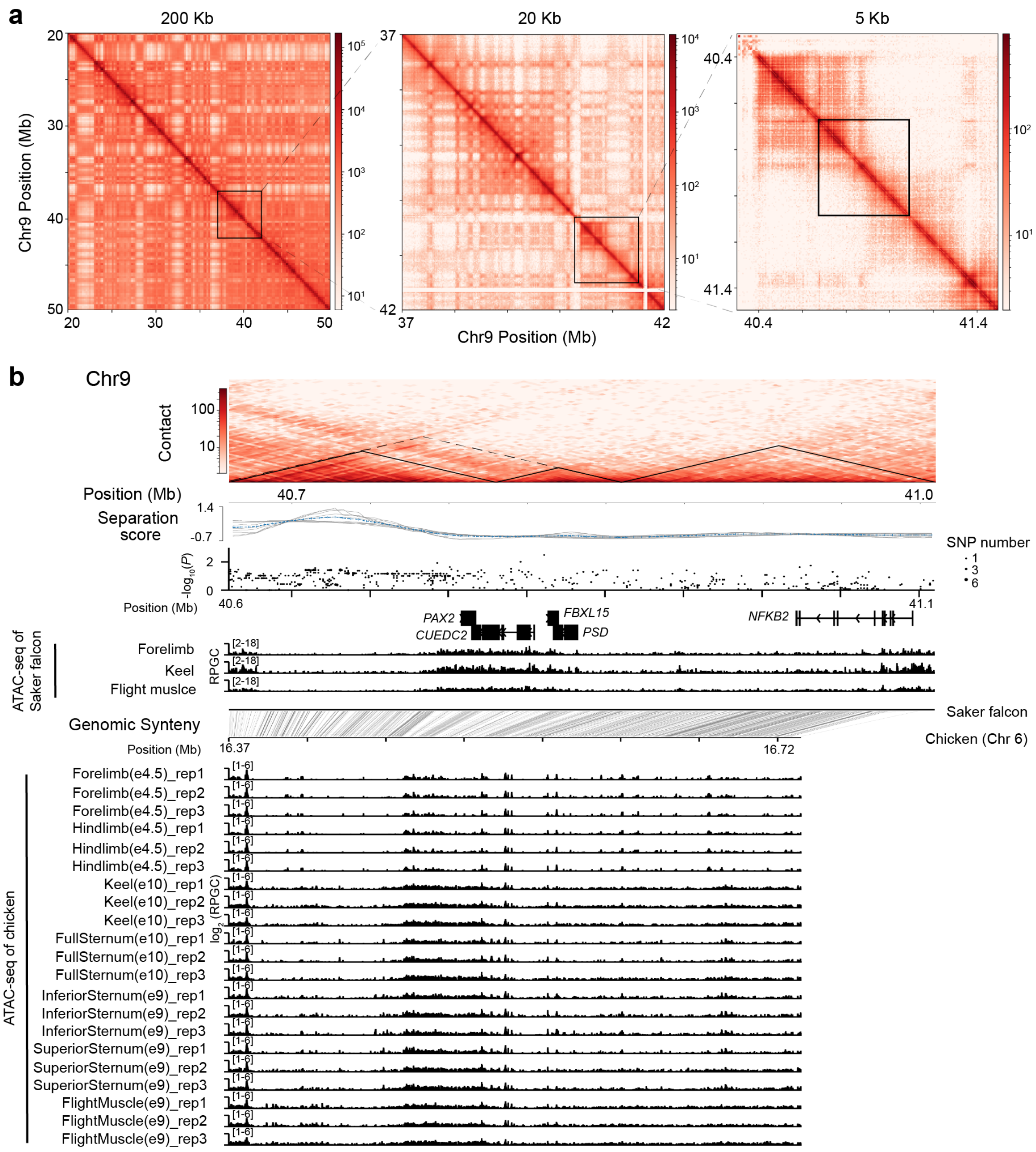

Supplementary Fig. 16. Identification of *cis*-regulatory elements (CREs) around *PAX2/FBXL15/NFKB2* genomic block using Hi-C and ATAC-seq data. a The heatmaps show the Hi-C contact matrix (bin size = 200 Kb, 20 Kb, 5 Kb) around *PAX2/FBXL15/NFKB2* gene block. **b** A zoom-in view of the black square in the 5 Kb bin size of **a**. The black triangles show the TAD structures (bin size = 5 Kb). The curves below the heatmap represent the TAD separation scores between the left and right regions at each bin with different window sizes (grey lines) and mean scores (blue lines).

The dot plot shows the logarithmically transformed *P*-value (*hapFLK* test) for each SNP calculated between the West and East saker populations. The ATAC-seq tracks (normalized using RPGC with $\log_2$ transformed) around the gene block were identified from saker and chicken embryonic tissue samples (data are available in the NCBI database under accession code PRJNA433154)[5]. The grey blocks show the syntenic regions of the focal genomic fragment between saker and chicken genomic sequences. The window size is 1 Kb.

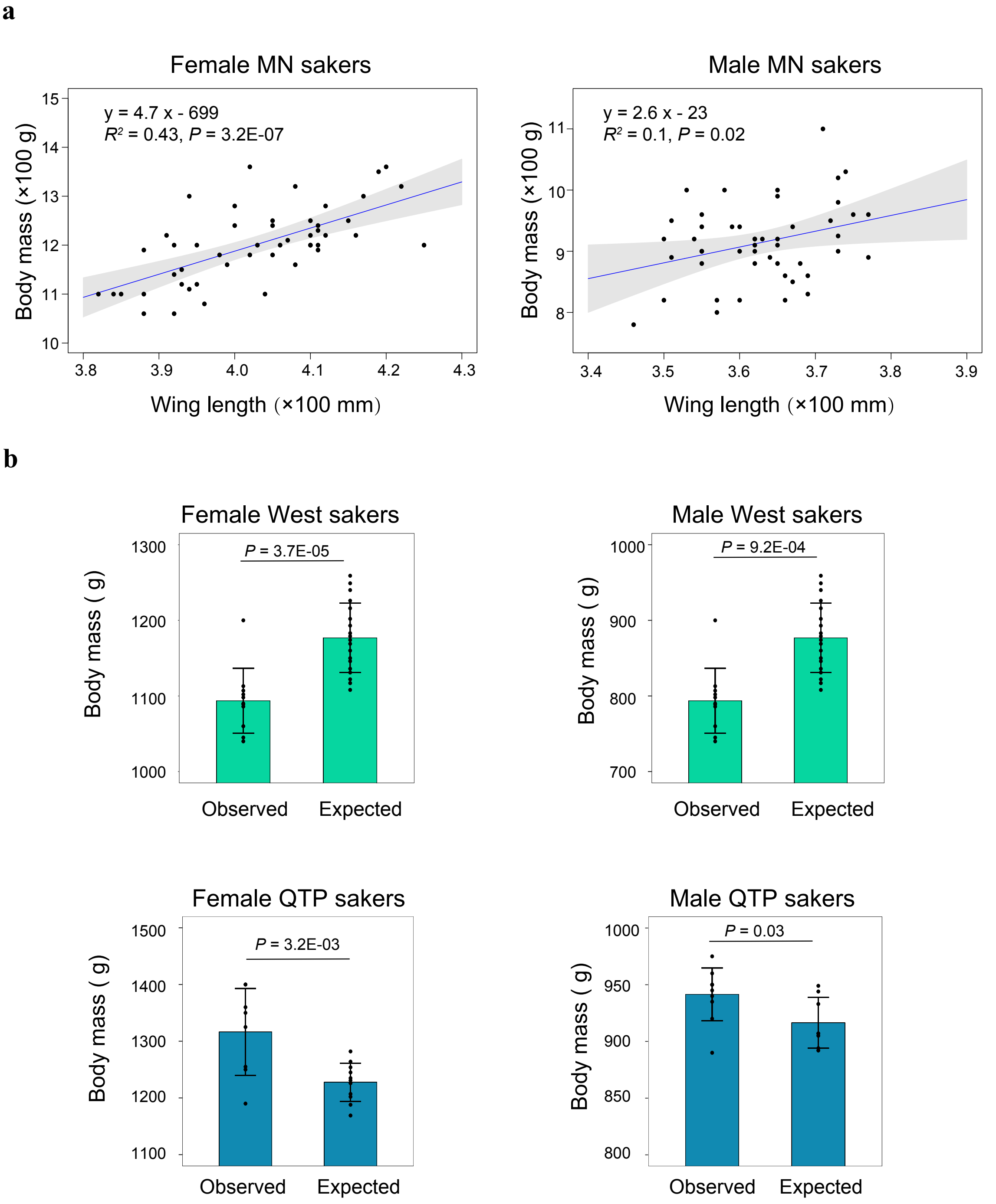

Supplementary Fig. 17. Body mass index analysis on West, MN and QTP saker populations. a Correlation between body mass and wing length for adult female ($N = 49$) and male MN sakers ($N = 48$)[3] using a linear regression model. The mean (blue line) and 95% CI (grey band) are shown. Significance level was calculated using F test. **b** Comparisons of the expected body masses of West[1,2,4] and QTP sakers (predicted using

the coefficient of MN sakers) with those observed in the field. West population: $N = 11$ (observed) and $N =19$ (predicted) for females; $N = 8$ (observed) and $N = 17$ (predicted) for males. QTP population: $N = 8$ (observed) and $N = 11$ (predicted) for females; $N = 10$ (observed) and $N = 8$ (predicted) for males. A two-sided *t*-test was used. The bars display mean ± SD. Source data are provided as a Source Data file.

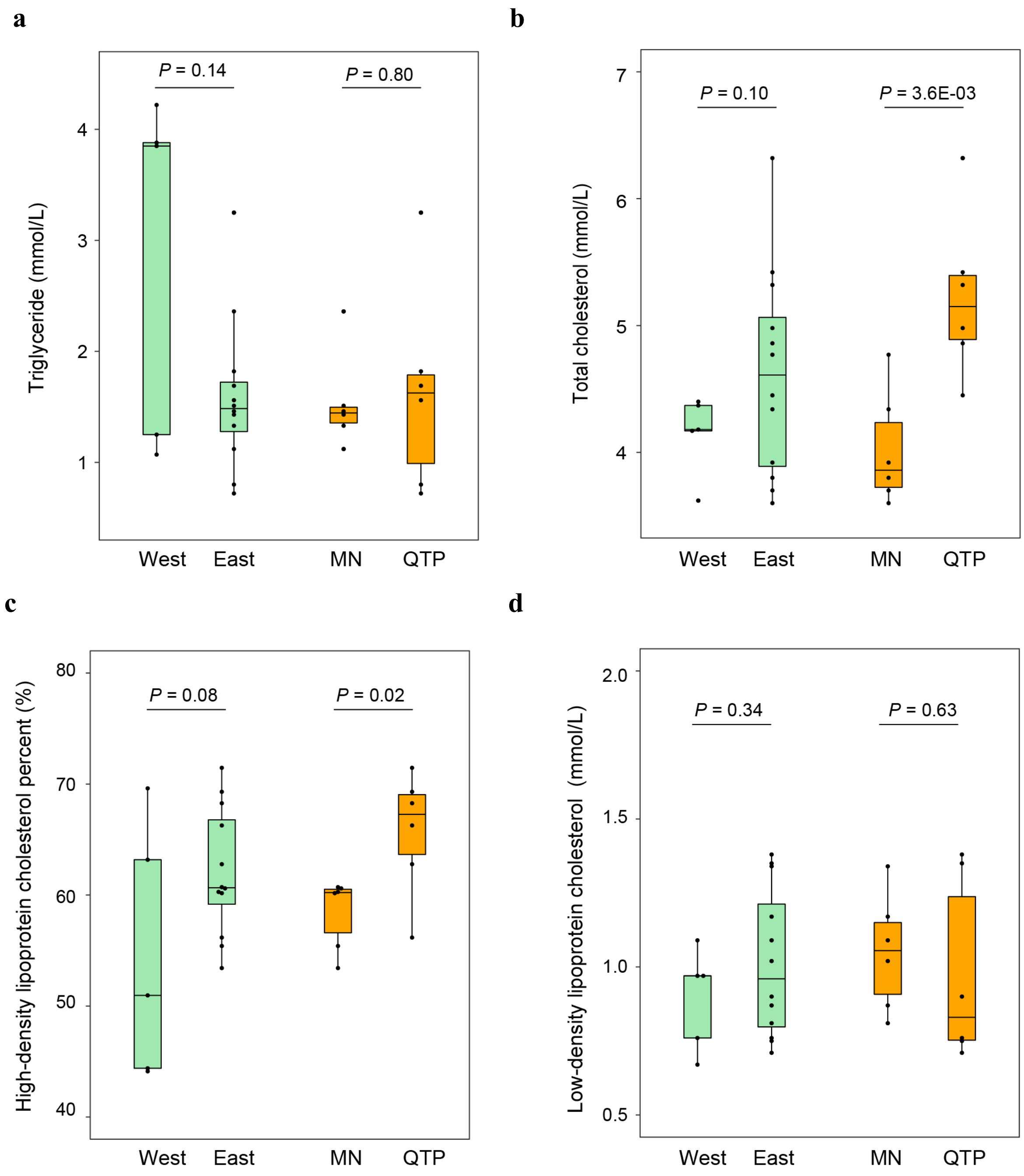

Supplementary Fig. 18. Comparisons of triglyceride (a) and total cholesterol (b) level, high-density lipoprotein cholesterol (HDLC) percent (c) and low-density lipoprotein cholesterol (LDLC) level (d) between West (*N* = 5 biologically independent samples) and East sakers (*N* = 12 biologically independent samples), MN (*N* = 6 biologically independent samples) and QTP saker populations (*N* = 6 biologically independent samples). A two-sided *t*-test was used. In the box plots, the centre line represents the median, whiskers represent maximum and minimum values,

and box boundaries represent 75th and 25th percentiles. Source data are provided as a Source Data file.

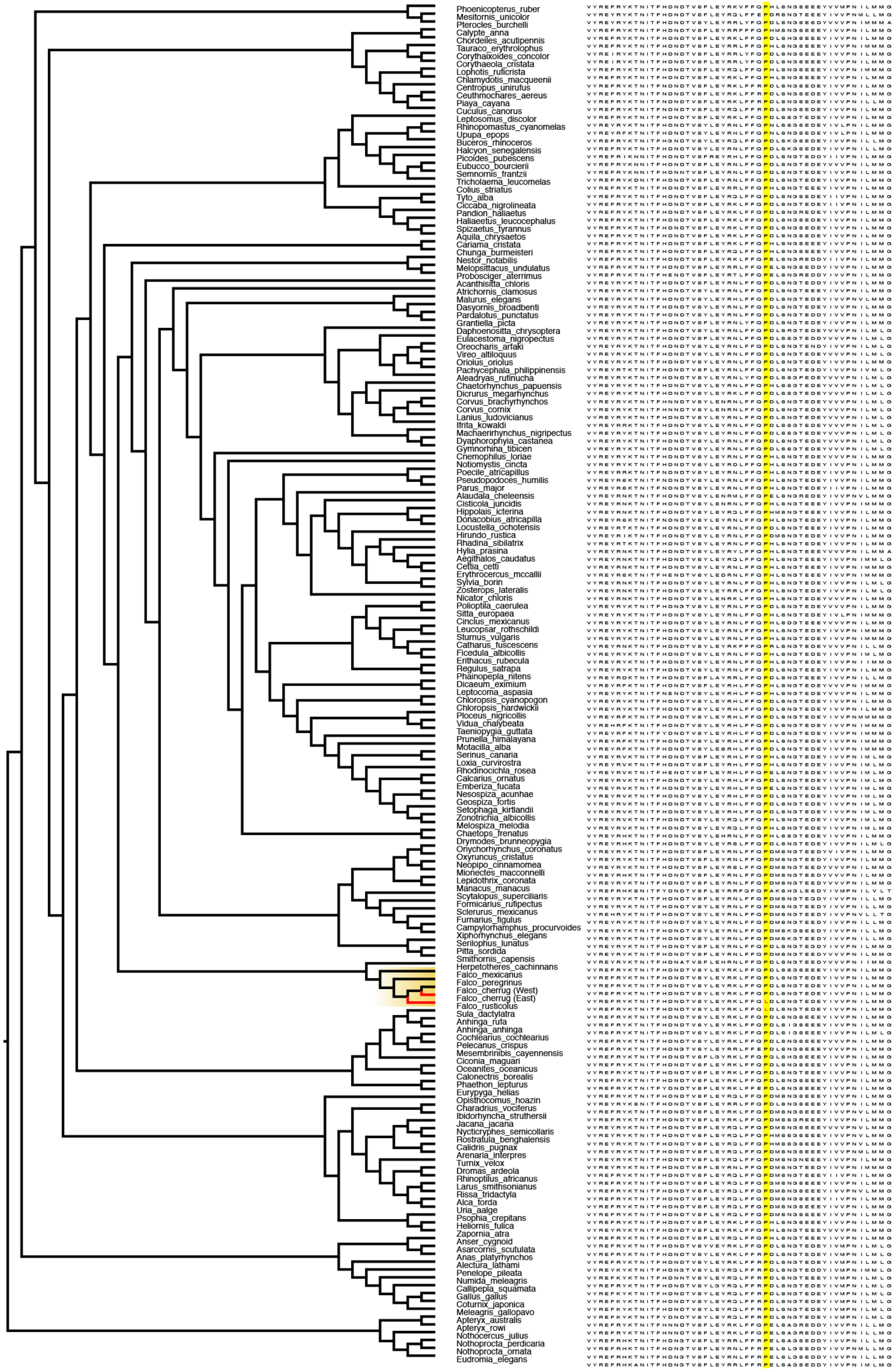

Supplementary Fig. 19. Alignment of the 121st amino acid in the SR-B1 of 172 avian species. The unique mutations in the gyrfalcon and East saker are highlighted.

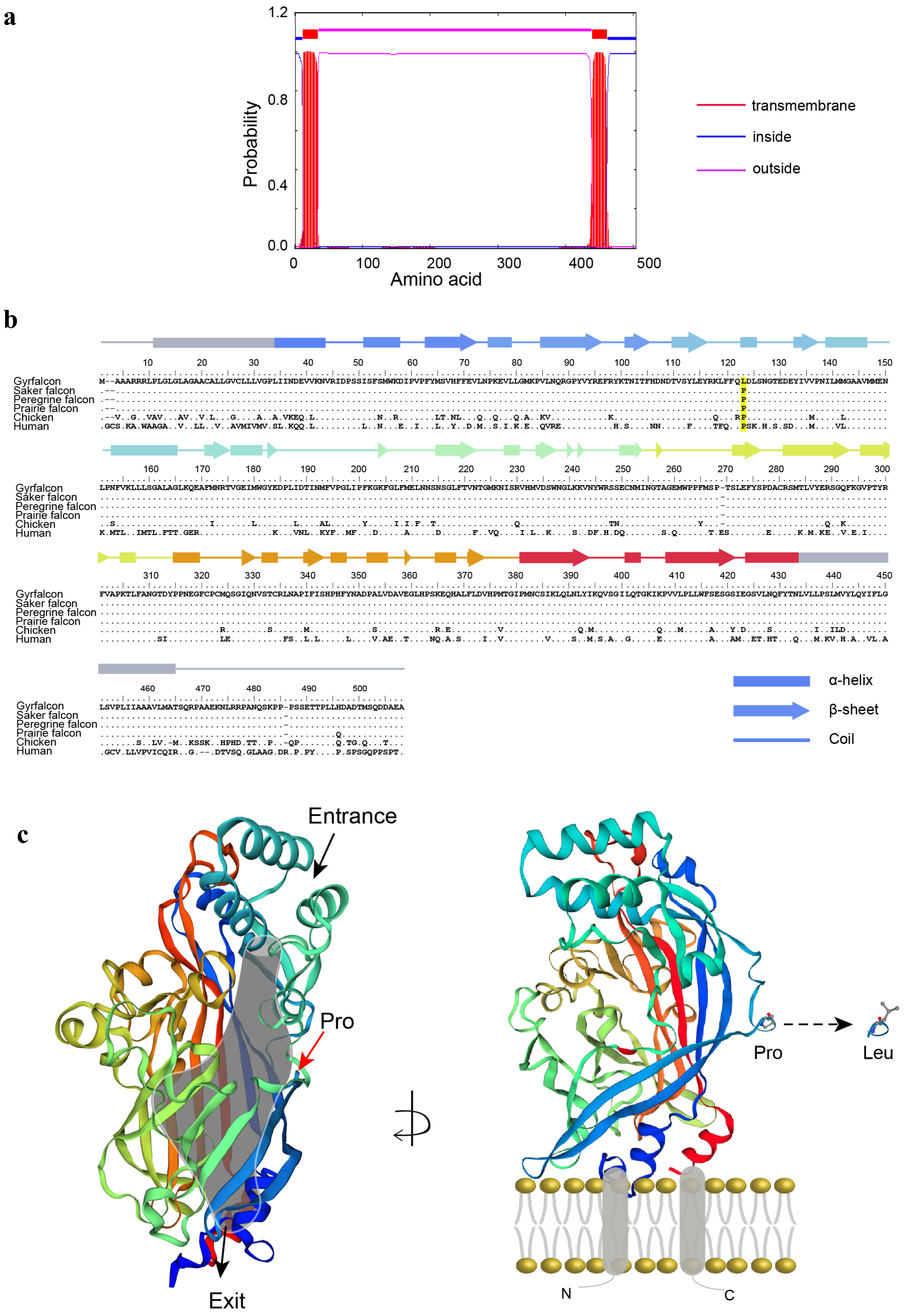

Supplementary Fig. 20. Prediction of the SR-B1 protein structure. a The predicted transmembrane helices of SR-B1 protein from *TMHMM2.0*. The red, blue and magenta represent amino acid residuals predicted trans, inside and outside of the membrane

respectively. **b** The alignment of the orthologous SR-B1 in human, chicken and falcons using *MAFFT*. Dots represent the same amino acids and hyphens mean indels. The SR-$B1^{P121L}$ mutations in falcons are highlighted. The secondary structure of SR-B1 is plotted from the *SWISS-MODEL* result. **c** The predicted 3D structure of SR-B1 from *SWISS-MODEL* result with LIMP-2 (code: 4F7B) model. Arrows show the entrance and exit of the tunnel. Block, arrow and line represent the predicted secondary structures: α-helix, β-sheet and coil, respectively. Different colors of secondary structures were the same as in **b**.

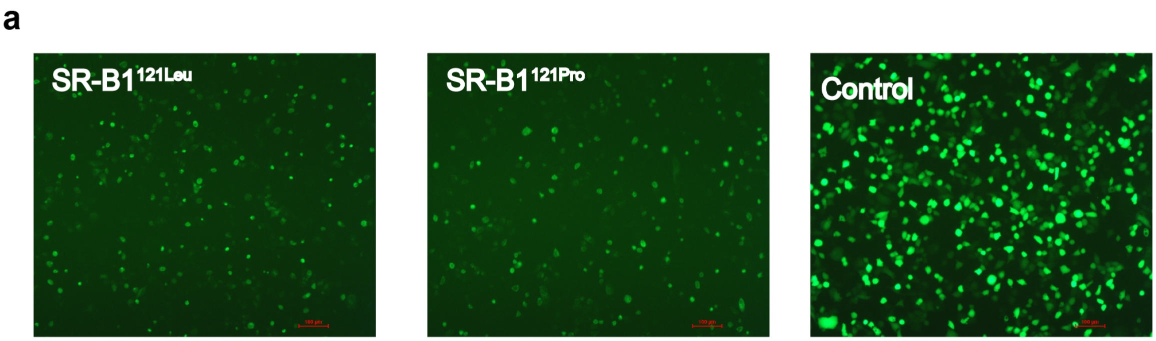

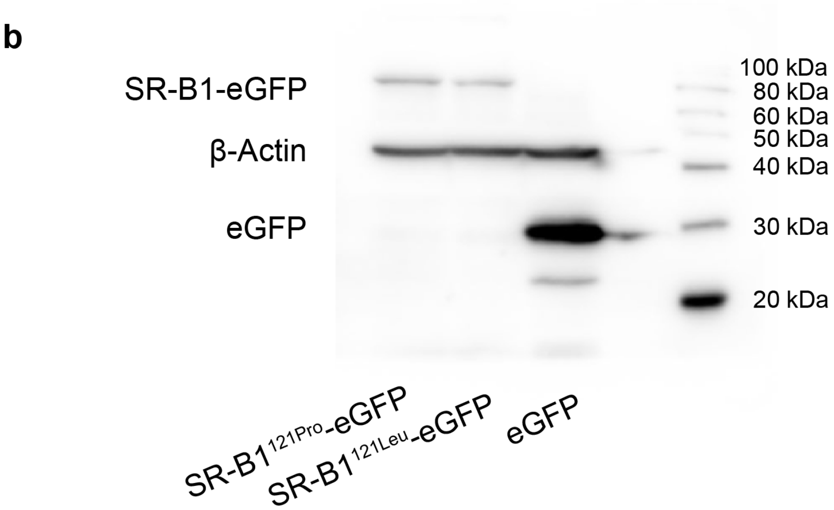

Supplementary Fig. 21. Validation of the SR-B1 protein expression. a The eGFP fluorescence in successfully transfected plasmids into human Hela cells. Expression was observed under a fluorescence optical microscope (10× magnification). Each experiment was repeated independently three times. **b** Western blot testing the expression of SR-B1-eGFP fusion proteins in Hela cells using eGFP antibody. β-Actin is an internal control. Left is the SR-B1-eGFP fusion proteins (~82 kDa) and right is the empty eGFP (~27 kDa). Each experiment was repeated independently three times.

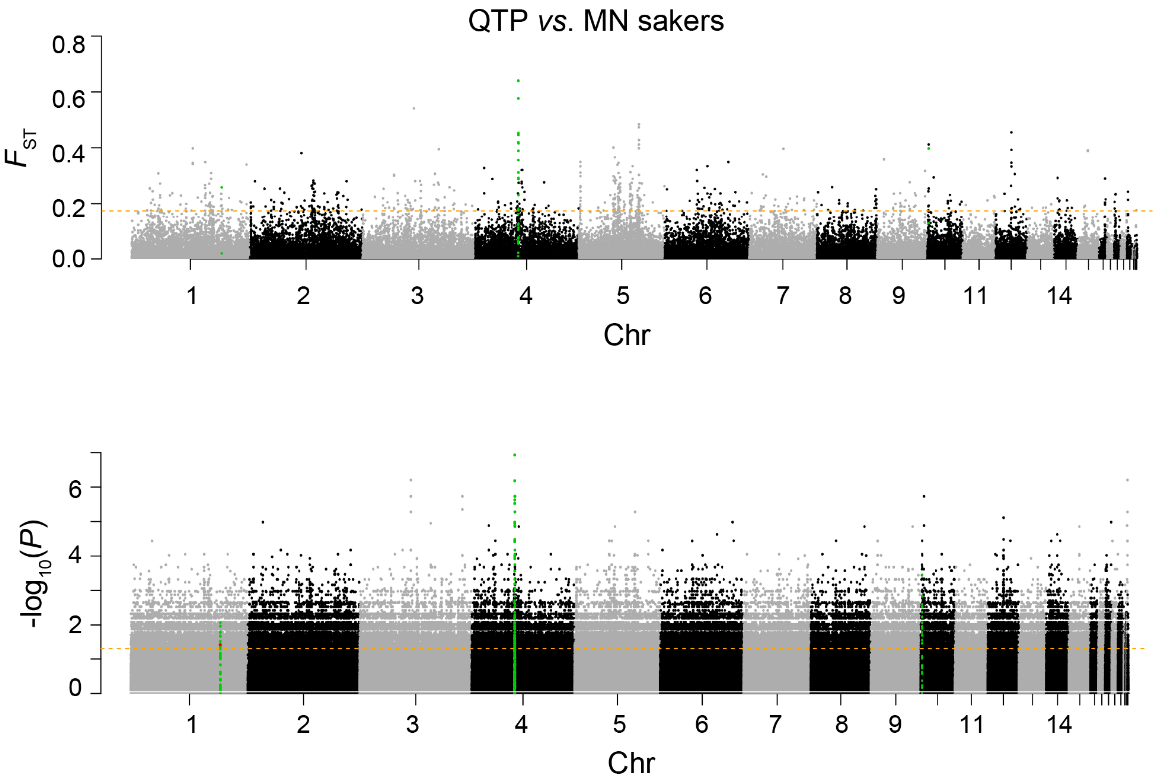

Supplementary Fig. 22. The genome-wide distribution of F_{ST} (20 Kb sliding windows) and logarithmically transformed *P*-value (*hapFLK* test) between the MN and QTP saker populations. The orange lines show the scores for F_{ST} (top 1% = 0.17) and *P*-value of *hapFLK* test ($P = 0.05$) respectively. The green dots represent the SNPs located in the focal sweep (Chr 4), *ASIP* (Chr 10) and *SCARB1* (Chr 1), respectively. The introgressed allele $SCARB1^{362}$ (Chr 1) is highlighted (red dot) in the *hapFLK* plot.

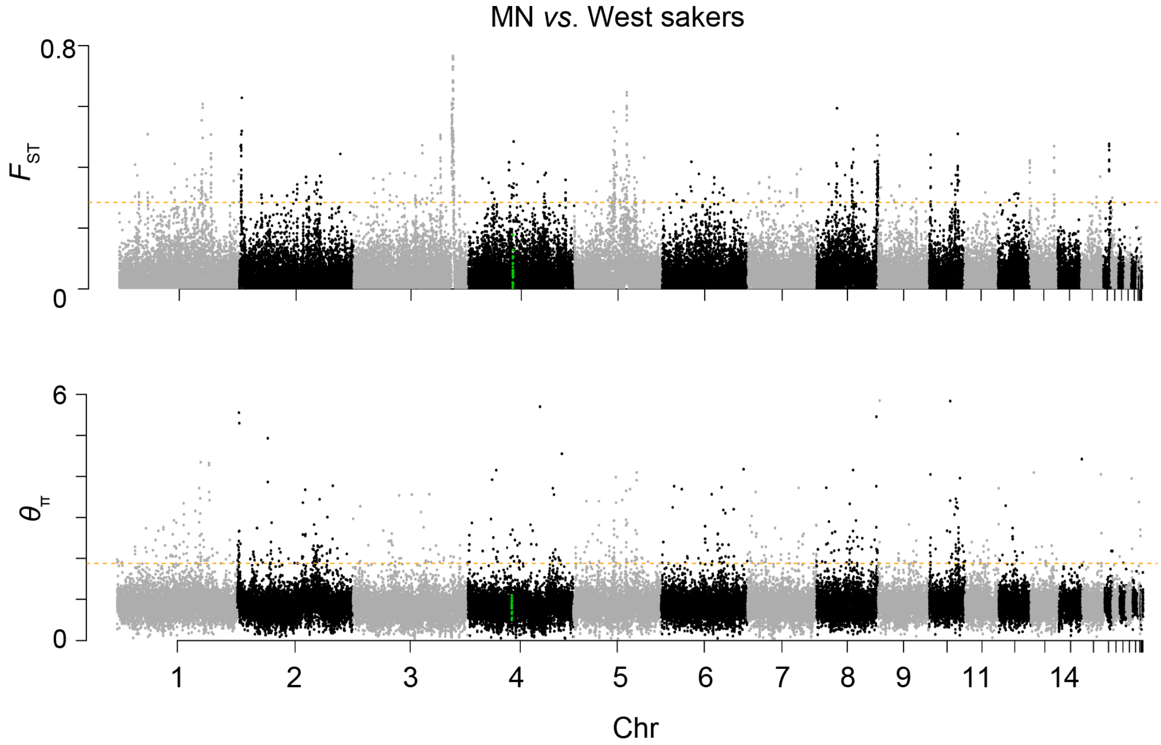

Supplementary Fig. 23. The genome-wide distribution of F_{ST} and θ_π (20 Kb sliding windows) between the MN and West saker populations. The orange lines show the scores for F_{ST} (top 1% = 0.28) and θ_π (top 1% = 1.88), respectively. The green dots represent the SNPs located in the focal sweep (Chr 4). The low F_{ST} and θ_π values of the focal region mean that this region is not selected in the MN and West saker populations.

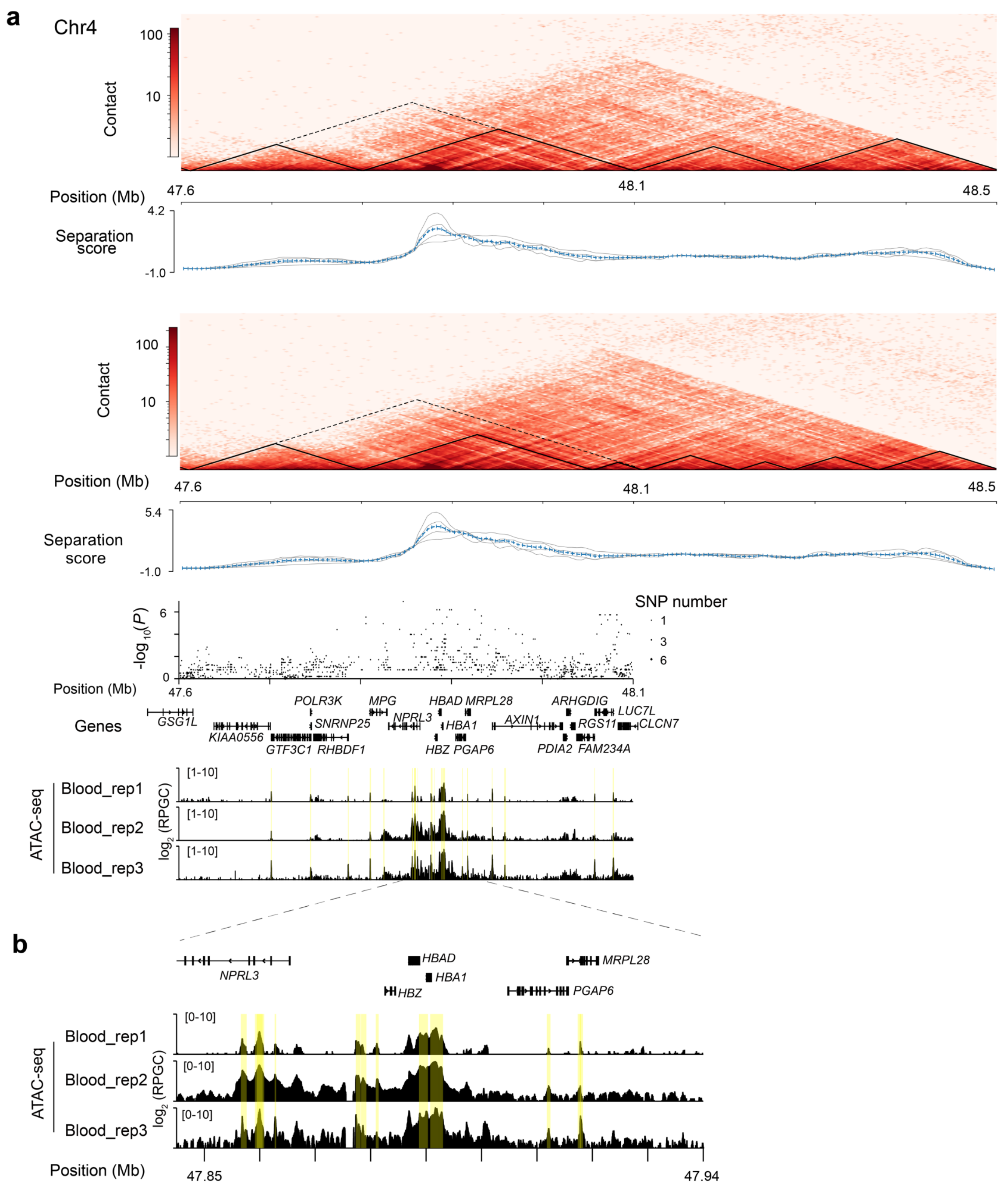

Supplementary Fig. 24. Prediction of CREs in the focal sweep of Chr 4 using ATAC-seq and Hi-C data from saker blood tissues. a The heatmap show the Hi-C contact matrix (bin size = 5 Kb) of the focal sweep in MN and QTP sakers. The solid and dash black triangles show the TAD structures in 5 Kb and 20 Kb bin size respectively. The curves below the heatmap represent the TAD separation scores between the left and right regions at each bin with different window sizes (grey lines) and mean scores (blue lines).

The dot plot shows the logarithmically transformed *P*-value (*hapFLK* test) for each SNP calculated between the MN and QTP saker populations. The ATAC-seq tracks (normalized using RPGC with log_2 transformed) were identified from three blood samples of QTP sakers, and yellow blocks show the reproducible peaks identified in at least two biological replicates. The window size is 1 Kb. **b** The zoom-in figure of the ATAC-seq tracks in the hemoglobin cluster. The window size is 200 bp.

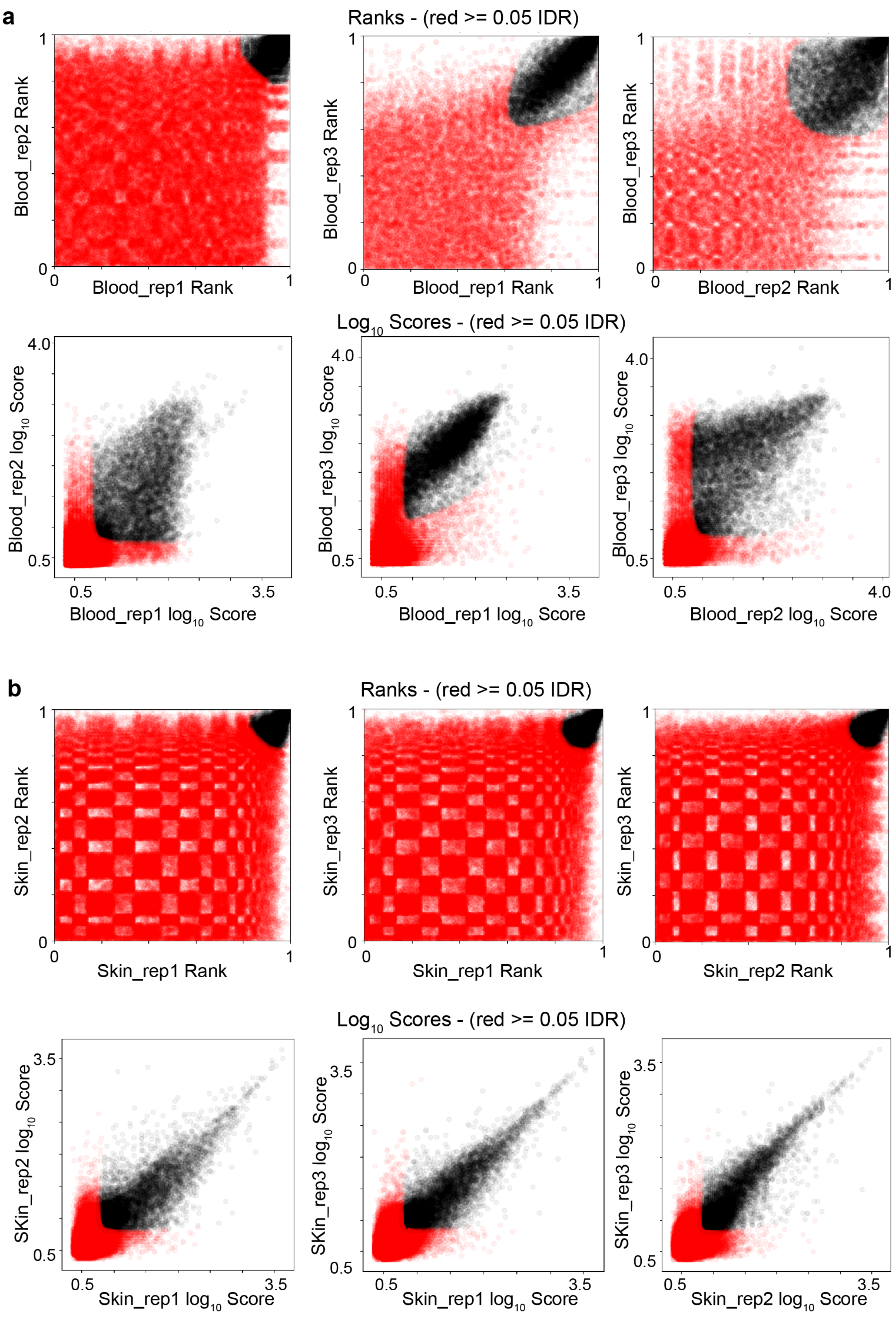
a
Ranks - (red >= 0.05 IDR)
Blood_rep2 Rank
Blood_rep1 Rank
Blood_rep3 Rank
Blood_rep1 Rank
Blood_rep3 Rank
Blood_rep2 Rank
0
1
Log10 Scores - (red >= 0.05 IDR)
Blood_rep2 log10 Score
Blood_rep1 log10 Score
Blood_rep3 log10 Score
Blood_rep1 log10 Score
Blood_rep3 log10 Score
Blood_rep2 log10 Score
0.5
3.5
4.0
b
Ranks - (red >= 0.05 IDR)
Skin_rep2 Rank
Skin_rep1 Rank
Skin_rep3 Rank
Skin_rep1 Rank
Skin_rep3 Rank
Skin_rep2 Rank
Log10 Scores - (red >= 0.05 IDR)
SKin_rep2 log10 Score
Skin_rep1 log10 Score
SKin_rep3 log10 Score
Skin_rep1 log10 Score
SKin_rep3 log10 Score
Skin_rep2 log10 Score

Supplementary Fig. 25. Consistence of peak calls between three biological replicates of blood (a) and skin (b) samples of QTP sakers using irreproducibility discovery rate (IDR) method. The black and red dots mean the reproducible and irreproducible peaks, respectively.

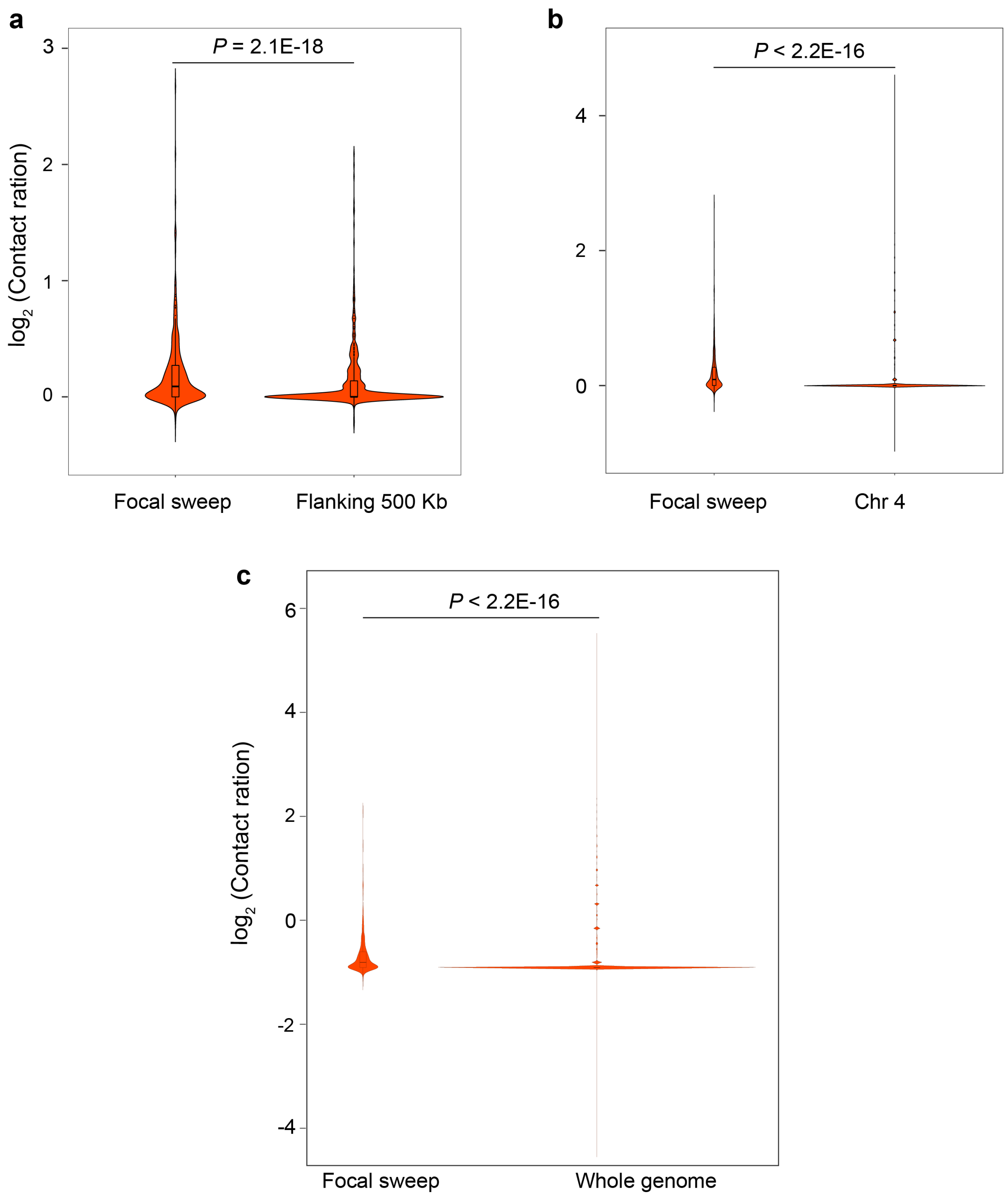

Supplementary Fig. 26. Comparisons of $\log_2$ (contact ratio of QTP ($N = 2$) / MN ($N = 2$) sakers) between the 500 Kb sweep region and flanking 500 Kb regions (a), between the TAD region and the whole Chr 4 (b), and between the TAD region and the whole genome (c), respectively. In the box plots, the centre line represents the median, whiskers represent maximum and minimum values, and box boundaries represent 75th and 25th percentiles. A two-sided *Wilcox* test was used.

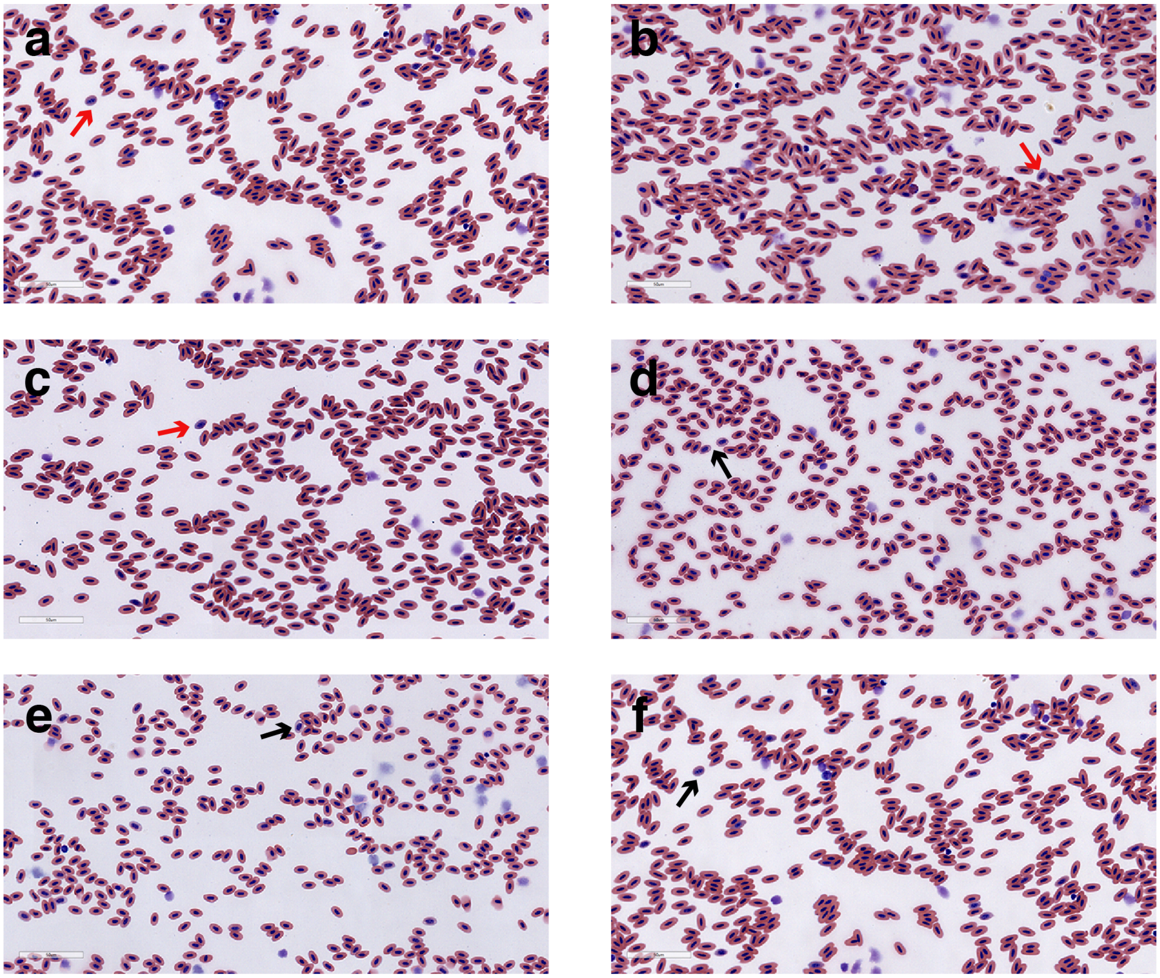

Supplementary Fig. 27. Representative circulating blood smears from saker falcons (a-c) and budgerigars (d-f). a-c one 6 months-old and two 4.5 years-old sakers. **d-f** 1.5, 3 and 6 months-old budgerigars. Five blood smears were produced from each individual, stained by Giemsa (Yeason) and scanned at 40× magnification using Aperio VESA8 system (Leica). For each smear, more than 700 cells were randomly selected for counting and identifying the immature erythrocytes. The proportions of immature erythrocytes in **a-f** individuals were 8.1%, 5.0%, 4.8%, 10.7%, 9.1%, 6.6% respectively. Red and black arrows show typical immature erythrocytes in sakers and budgerigars, respectively. The white bars scale 50 μm.

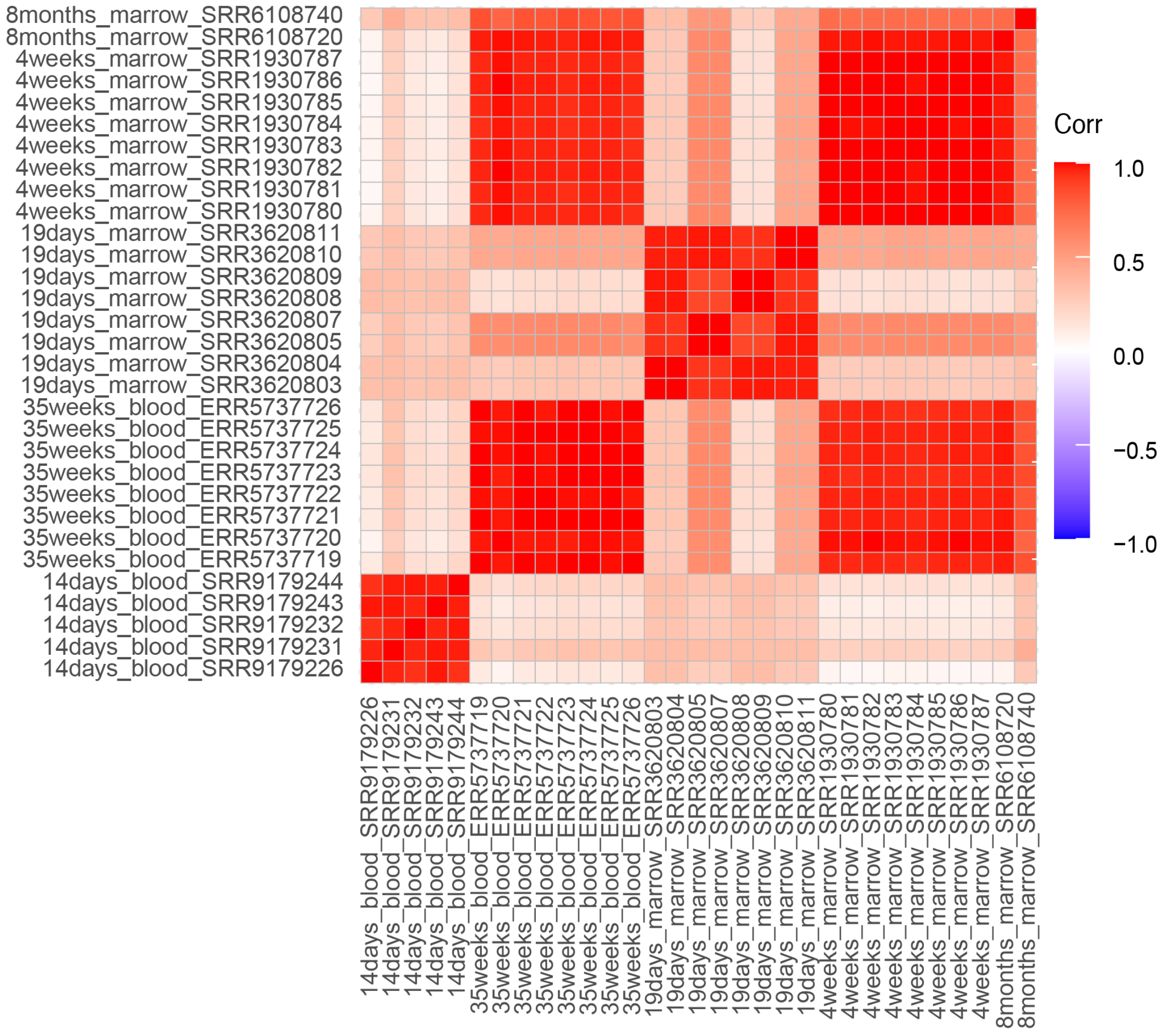

Supplementary Fig. 28. The positive correlation of expression pattern between chicken circulating blood and bone marrow transcriptomes. The transcriptome data including blood (14-days-old[5] and 35-weeks-old) and marrow tissues (19-days-old, 4-weeks-old[6] and 8-months-old[7]) were downloaded from NCBI. The bar shows the Spearman's correlation coefficient from low (blue) to high (red). Data used in this analysis are available in the NCBI database under accession codes PRJNA542984, PRJEB44038, PRJNA323973, PRJNA279487, and PRJNA412404.

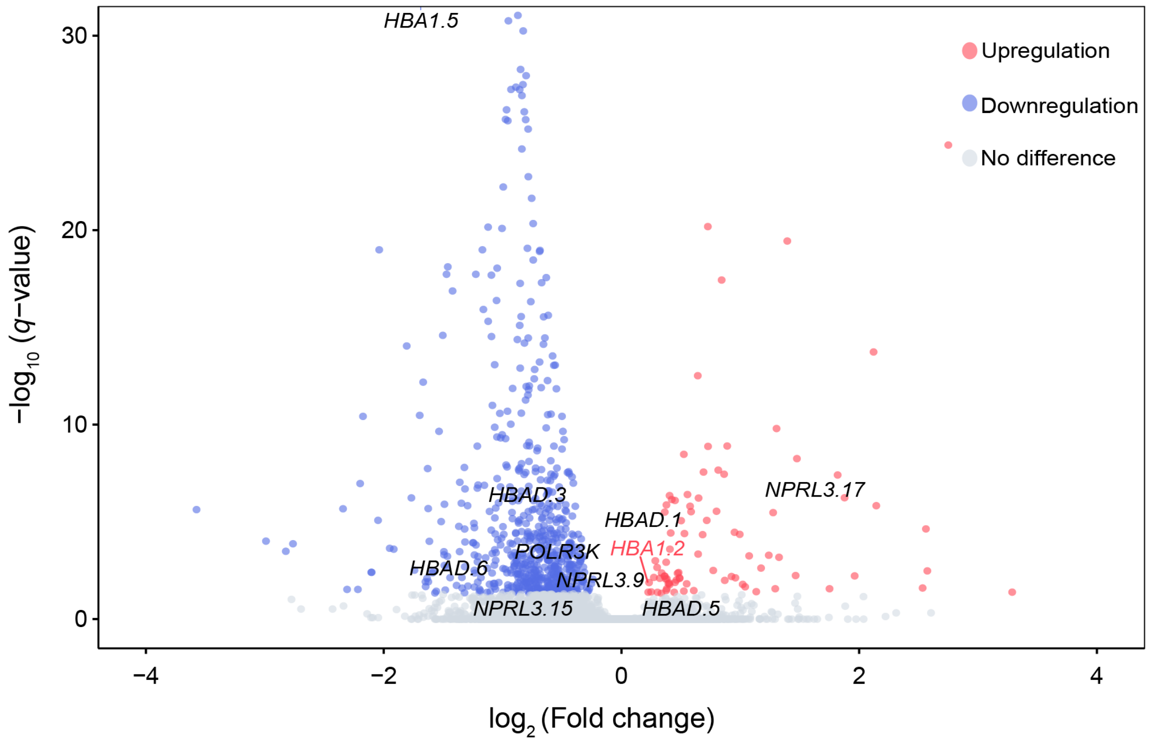

Supplementary Fig. 29. Volcano plot for differential expression level of each transcript in blood samples of MN (*N* = 9 biologically independent samples) and QTP sakers (*N* = 10 biologically independent samples). The x-axis is the fold change, and y-axis is the *q*-value, the probability that a transcript has a statistical significance.

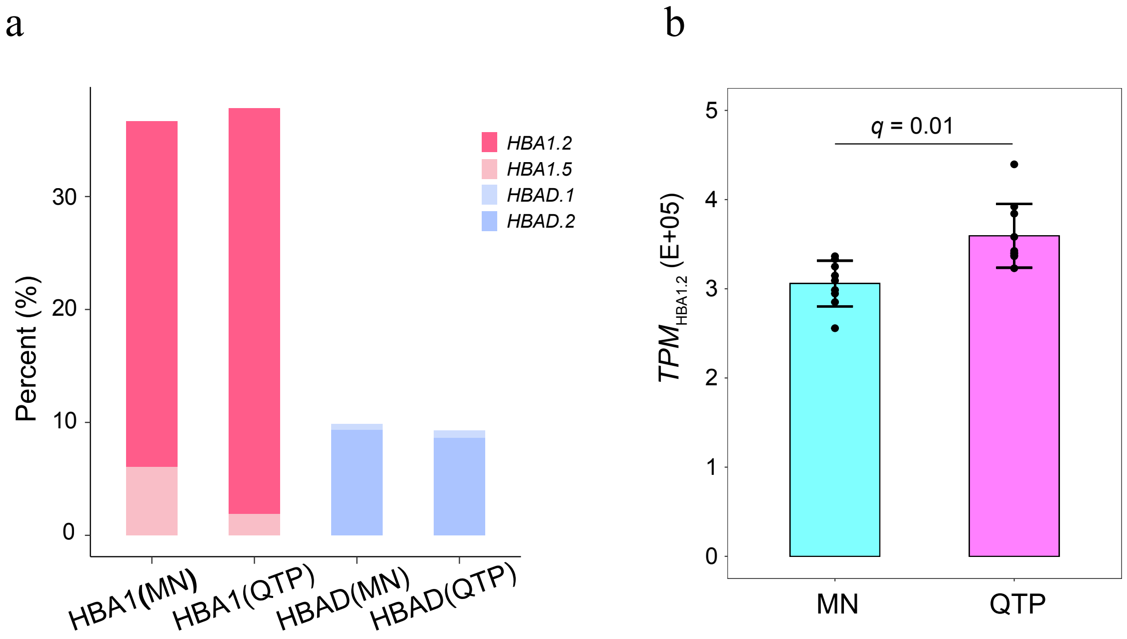

Supplementary Fig. 30. Expression of different hemoglobin transcripts. a The contributions of main expressed transcripts of *HBA1* and *HBAD* genes to the total expression of the whole transcriptome in the blood samples of MN (N = 9 biologically independent samples) and QTP sakers (N = 10 biologically independent samples). **b** Differential expression of the dominant transcript of *HBA1* gene (*HBA1.2*) between MN (N = 9 biologically independent samples) and QTP (N = 10 biologically independent samples) saker populations. The bars display mean ± SD. A differentially expressed transcript was detected using *edgeR* based on an exact test and P-value was adjusted by the FDR method.

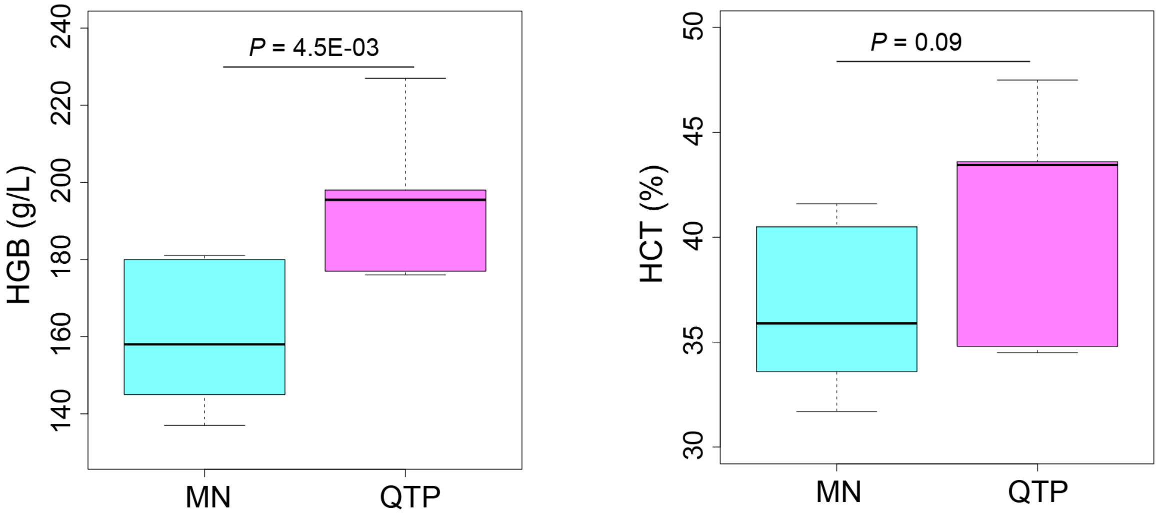

Supplementary Fig. 31. The HGB and HCT measurements in the blood samples of MN (*N* = 8 biologically independent samples) and QTP sakers (*N* = 6 biologically independent samples). HGB and HCT are abbreviations for hemoglobin and hematocrit. A two-sided *t*-test was used. In the box plots, the centre line represents the median, whiskers represent maximum and minimum values, and box boundaries represent 75th and 25th percentiles. Source data are provided as a Source Data file.

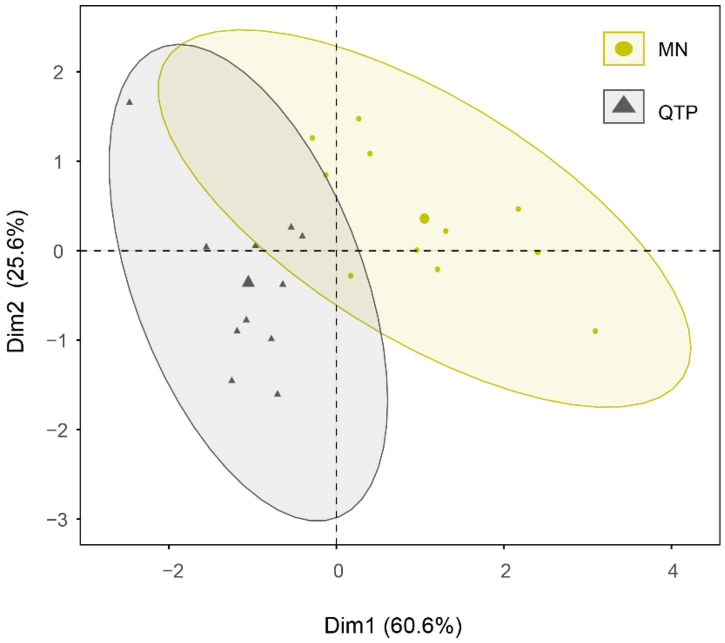

Supplementary Fig. 32. The PCA result of plumage colours (mean L*, a* and b* values) for all saker individuals in MN (*N* = 11) and QTP populations (*N* = 11). The black triangles and yellow dots show QTP and MN individuals, respectively. Source data are provided as a Source Data file.

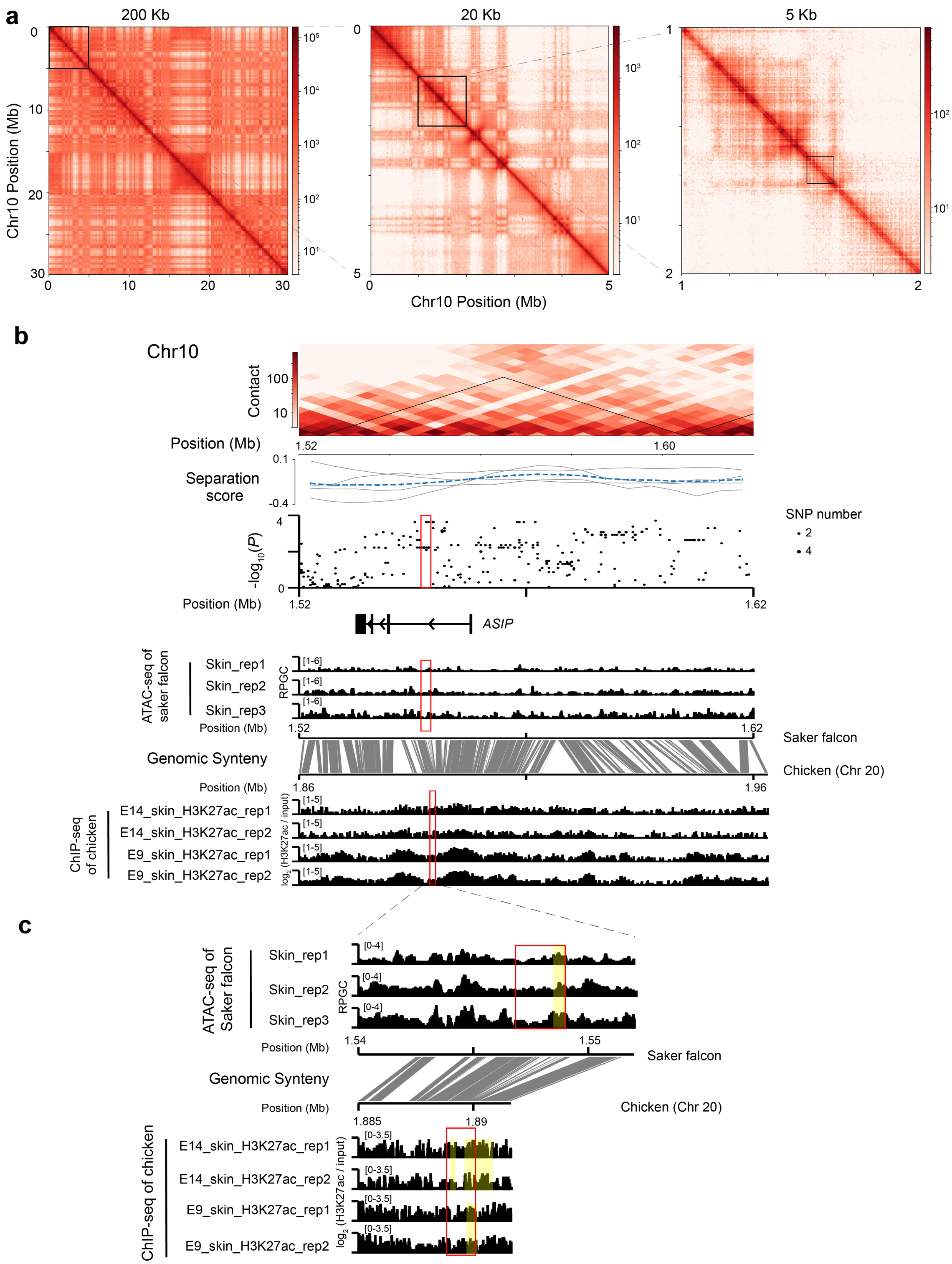

Supplementary Fig. 33. Identification of CREs around *ASIP* gene using ATAC-seq and Hi-C data. a The heatmaps show the Hi-C contact matrix (bin size = 200 Kb, 20 Kb, 5 Kb) around *ASIP* gene. **b** A zoom-in view of the black square in the heatmap of 5 Kb

bin size of **a**. The black triangles show the TAD structures (bin size = 5 Kb). The curves below the heatmap represent the TAD separation scores between the left and right regions at each bin with different window sizes (grey lines) and mean scores (blue lines). The dot plot shows the logarithmically transformed *P*-value (*hapFLK* test) for each SNP calculated between the MN and QTP saker populations. The ATAC-seq tracks (normalized using RPGC) of saker around *ASIP* gene were identified from dorsal skin samples of three juveniles. The ChIP-seq tracks of chicken[9] ((normalized using log_2(read counts of (H3K27ac/input))) around *ASIP* gene were identified from embryonic leg scale skin tissues (data used in this analysis are available in the NCBI database under accession code PRJNA561632). The red box circles the fragment cloned for the luciferase reporter assay in saker and orthologous sequences in chicken. The grey blocks show the syntenic regions between saker and chicken genomic sequences. The window size is 200 bp. **c** The zoom-in view of the ATAC-seq tracks of saker and H3K27ac ChIP-seq tracks of chicken around the focal fragment for luciferase experiment. The yellow blocks show the reproducible peaks identified in at least in two biological replicates using BEDTools (> 50% overlapping rate). The window size is 50 bp.

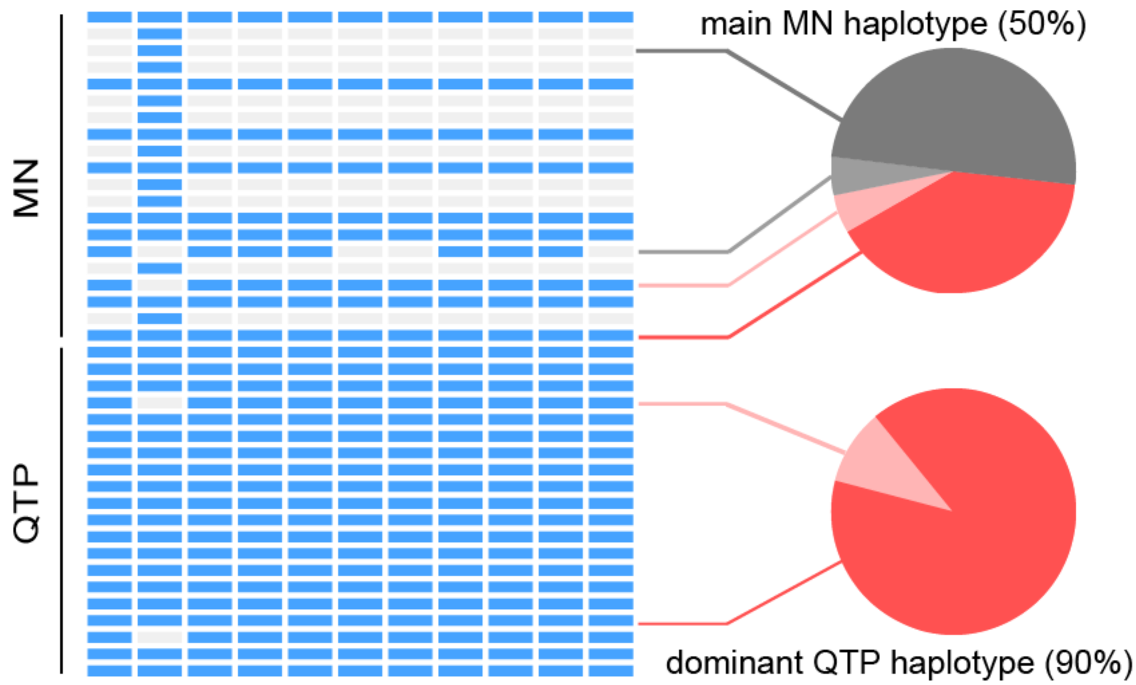

Supplementary Fig. 34. Haplotypes of the focal CRE on *ASIP* gene in MN and QTP saker populations. The blue and light grey squares symbol different alleles in each column (SNP). The pie plots show the haplotype frequency in the identified segment.

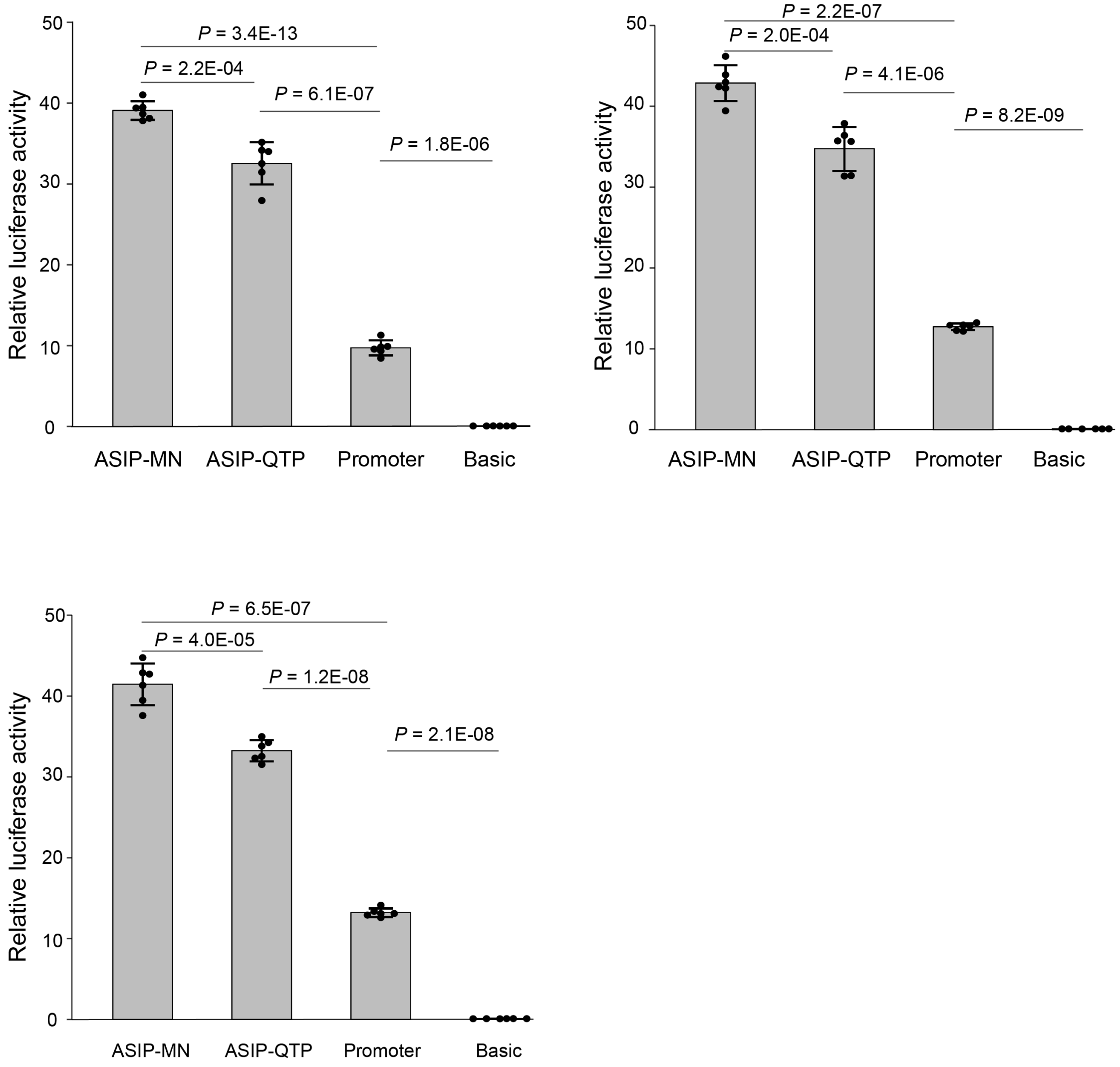

Supplementary Fig. 35. Three biologically independent replicates of luciferase reporter assay for the MN-dominant haplotype and QTP-dominant haplotype of the focal *cis*-regulatory element in *ASIP* gene. The ASIP-MN and ASIP-QTP groups were cloned into pGL3-Promoter vectors. The Promoter (pGL3-Promoter) and Basic (pGL3-Basic) groups were used as controls respectively. The bars display mean ± SD ($N = 6$ technical replicates). A two-sided *t*-test was used. Source data are provided as a Source Data file.

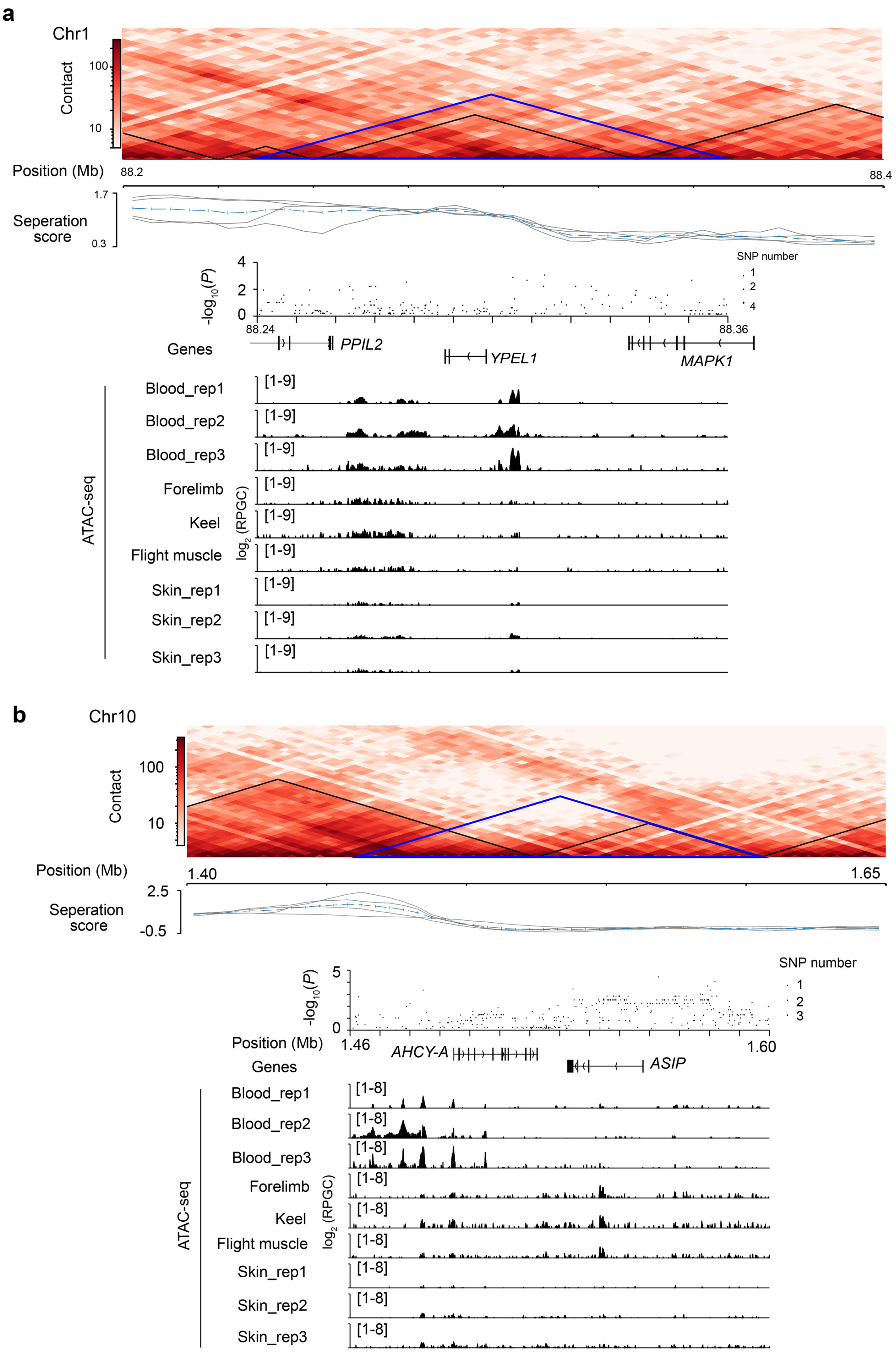
a
Chr1
Contact
100
10
Position (Mb)
88.2
88.4
Seperation score
1.7
0.3
-log10(P)
4
2
0
SNP number
1
2
4
88.24
88.36
Genes
PPIL2
YPEL1
MAPK1
ATAC-seq
log2 (RPGC)
Blood_rep1
Blood_rep2
Blood_rep3
Forelimb
Keel
Flight muscle
Skin_rep1
Skin_rep2
Skin_rep3
[1-9]
b
Chr10
Contact
100
10
Position (Mb)
1.40
1.65
Seperation score
2.5
-0.5
-log10(P)
5
0
SNP number
1
2
3
Position (Mb)
1.46
1.60
Genes
AHCY-A
ASIP
ATAC-seq
log2 (RPGC)
Blood_rep1
Blood_rep2
Blood_rep3
Forelimb
Keel
Flight muscle
Skin_rep1
Skin_rep2
Skin_rep3
[1-8]

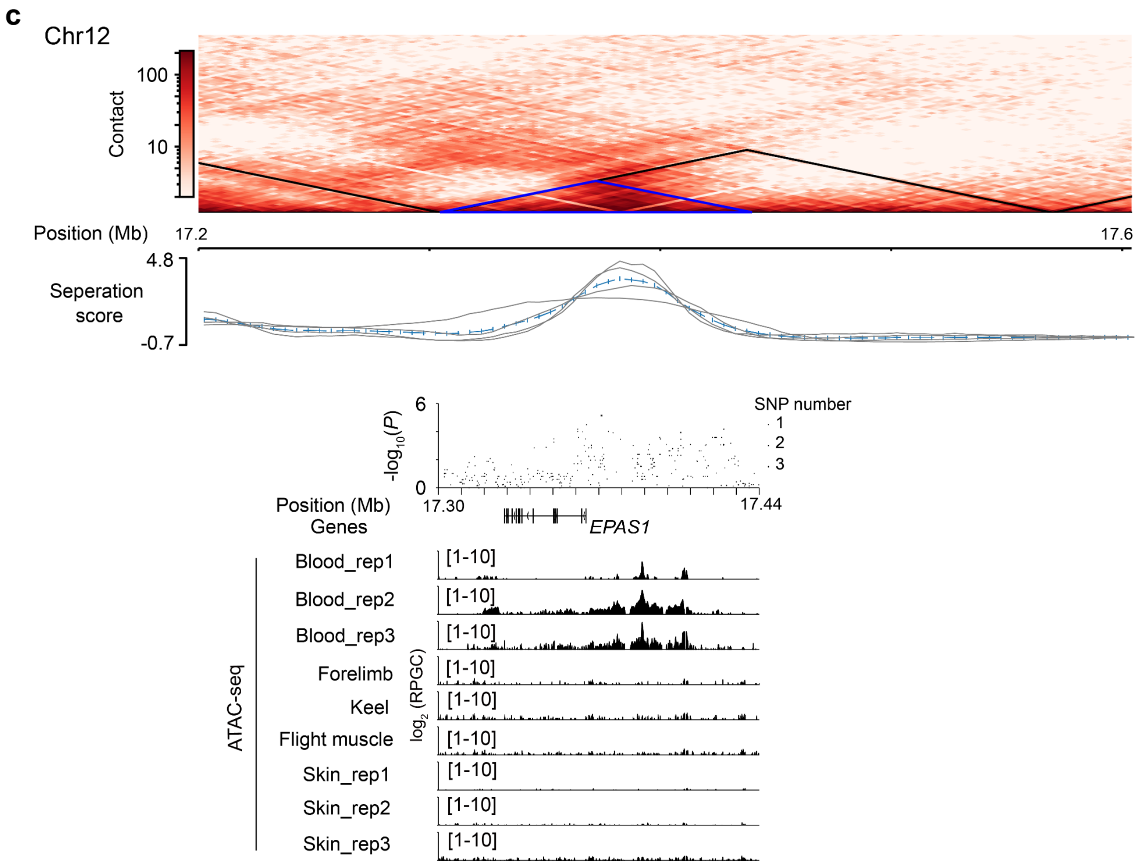

Supplementary Fig. 36. TAD structure in three selected sweeps. The heatmaps show the Hi-C contact matrix (bin size = 5 Kb) around *PPIL2/YPEL1/MAPK1* (a), *AHCY-A/ASIP* (b) and *EPAS1* (c) gene blocks selected in QTP sakers. The blue triangles show the bins covering the sweeps in QTP saker populations. The black triangles represent the TAD structures. The curves below the heatmap represent the TAD separation scores between the left and right regions at each bin with different window sizes (grey lines) and mean scores (blue lines). The dot plot shows the logarithmically transformed *P*-value (*hapFLK* test) for each SNP calculated between the MN and QTP saker populations. The ATAC-seq tracks (normalized using RPGC and with log_2 transformed) around genes were identified using data from different tissues (three biologically independent blood samples, forelimb, keel, flight muscle, and three biologically independent dorsal skin samples) of QTP sakers. The window size for both dot plots and ATAC-seq tracks is 200 bp.

Supplementary Tables

Supplementary Table 1. Statistics of multi-platform sequencing data for the saker genome assembly

Platform	Sequencing data			
PacBio	Subread bases (Gb)	Subread number (M)	Mean subread length (Kb)	Subread N50 length (Kb)
	97.52	8.37	11.65	18.19
HiSeq	Insert size (bp)	Read number (M)	Read length (bp)	Total bases (Gb)
	350	597.18	150	89.58
Bionano	Clean data quantity (Gb)	Clean data AvgLab (/100 Kb)	Clean data N50 (Kb)	
	307.45	15.65	235	

Note: AvgLab means the average number of labels per 100 Kb for total DNA.

Supplementary Table 2. Statistics of Hi-C data from MN and QTP sakers

Sample	Clean read number (G)	Clean bases (Gb)	Clean Q30 base rate (%)	Proportion of unique mapping paired-end reads (%)
MN1	2.1	341.4	90.94	73.20
MN2	1.1	166.5	92.20	78.57
QTP1	1.6	239.6	95.80	77.30
QTP2	2.8	449.4	91.95	75.14

Note: QTP1 sample was used for the genome assembly.

Supplementary Table 3. Summary of the saker genome assembled using PacBio and Bionano data

	PacBio		Bionano			
Size	Contig length (bp)	Contig number	Contig length (bp)	Contig number	Scaffold length (bp)	Scaffold number
N50	28,269,230	15	24,410,233	16	36,046,907	12
N60	22,682,656	20	18,391,551	22	24,891,951	17
N70	13,636,086	28	12,684,479	30	22,682,656	22
N80	9,572,453	38	8,469,167	41	14,224,935	29
N90	4,323,552	57	3,969,850	61	6,470,921	41
Longest	81,963,286	1	81,963,286	1	81,963,286	1
Total	1,227,251,291	1,356	1,227,250,147	1,390	1,235,113,680	1,313

Note: the lengths of scaffolds and total genome include "*N*".

Supplementary Table 4. Summary of the final assembled saker genome

ChrID	Scaffold number	Length (bp)	ChrID	Scaffold number	Length (bp)
Chr 1	10	128,708,988	Chr 15	5	23,859,303
Chr 2	7	123,578,221	Chr 16	3	8,317,540
Chr 3	18	123,083,686	Chr 17	2	7,709,136
Chr 4	21	113,721,770	Chr 18	11	7,087,382
Chr 5	14	93,972,130	Chr 19	4	6,885,157
Chr 6	5	92,336,655	Chr 20	2	6,426,764
Chr 7	7	73,931,608	Chr 21	10	1,924,614
Chr 8	4	65,992,661	Chr 22	9	1,329,381
Chr 9	4	54,773,600	Chr 23	5	970,468
Chr 10	2	38,426,598	Chr 24	14	788,709
Chr 11	6	36,121,347	Chr Z	28	86,275,077
Chr 12	4	34,217,874	Chr W	49	21,988,858
Chr 13	4	30,074,843	UN	976	27,485892
Chr 14	15	25,156,918			

Note: the length includes "*N*". "UN" means the un-anchored scaffolds.

Supplementary Table 5. Comparisons of the current and previous saker genome assemblies

	Current assembly	Current approach	Previous assembly	Previous approach
Contig N50 number	16	PacBio/ Illumina /Bionano	10,998	Illumina (170bp, 500bp, 800bp, 2 Kb, 5 Kb, 10 Kb, 20 Kb libraries)[10]
Contig N50 length (bp)	24,410,233		31,237	
Scaffold N50 number	12		84	
Scaffold N50 length (bp)	36,046,907		4,154,532	
Longest scaffold length (bp)	81,963,286		19,410,955	
Total scaffold number	1,239		30,431	
Total assembly genome size (bp) includes *N*	1,235,113,680		1,177,902,945	
Anchored chromosome number	24 autosomes + ZW pair	Hi-C	19 autosomes + Z	RACA/FISH[11]
Anchored genome length (bp)	1,207,659,088		1,055,312,481	
Gene number	16,449	Homolog/ *De novo*/RNA method	16,204	Homolog/*De novo*/RNA method
Average gene length (bp)	20,336		19,314	

Supplementary Table 6. Summary of the f_3-statistic

Target (A)	Source 1 (B)	Source 2 (C)	f_3 mean	Std. err	*Z*-score
East saker	Gyrfalcon	West saker	-0.021390	0.000893	-23.956
West saker	Gyrfalcon	East saker	0.089363	0.003261	27.408
Gyrfalcon	West saker	East saker	0.265560	0.008164	32.527

Note: West sakers are assumed to be ancestors of East saker. If f_3 (A; B, C) is significantly negative (*Z*-score < -3), then A is considered as an admixed strain from B and C. Std. err is abbreviated for standard error.

Supplementary Table 7. Simulation parameters used for *fastsimcoal2*

Variable	Parameters	Minimum	Maximum
Population effective size	Recent MN sakers	15,000	20,000
	Recent QTP sakers	15,000	30,000
	Ancestor of MN and QTP sakers	10,000	15,000
	Recent West sakers	6,000	11,000
	Ancestor of West and East sakers	10,000	15,000
	Recent gyrfalcons	3,000	8,000
	Ancestor of sakers and gyrfalcons	15,000	20,000
Generation time of historical events	Divergence between QTP and MN sakers	1,200	2,000
	Hybridization between gyrfalcons and East sakers	2,100	4,000
	The divergence between East and West sakers	5,500	13,000
	Population contraction of gyrfalcons	15,000	16,600
	Divergence between sakers and gyrfalcons	36,000	53,000
Introgression rate	Gyrfalcons to East sakers	0.2	0.3
	East sakers to Gyrfalcons	0.005	0.01

Supplementary Table 8. Statistics of ATAC-seq data from QTP sakers

Sample name	Clean read number (M)	Clean base (Gb)	Clean Q30 base rate (%)	Mapping rate (%)
Forelimb	92.85	10.11	90.72	56.29
Keel	88.40	9.43	92.28	43.42
Flight muscle	160.50	16.45	88.81	48.92
Blood_rep1	146.88	16.67	93.84	44.98
Blood_rep2	75.55	9.29	91.03	85.52
Blood_rep3	52.60	8.57	93.69	93.69
Skin_rep1	89.53	9.58	92.82	82.39
Skin_rep2	88.13	9.91	93.36	84.75
Skin_rep3	81.34	9.00	92.98	85.24

Supplementary Table 9. Adaptively introgressed genes related to body size development

Gene name	Functional description
SCMH1	Sex comb on midleg homolog 1, acts as an E3 ubiquitin ligase to suppress the expression of *HOX* genes[12].
FOXO6	Forkhead box O6, regulates Hippo signaling. The *FOXO6*$^{-/-}$ mice have larger heads[13].
HMGA2	High mobility group AT-hook 2, variant of *HMGA2* is associated with adult and childhood height in human, and *HMGA2*$^{-/-}$ mice exhibit smaller size[14,15].
MSRB3	Methionine sulfoxide reductase B3, is involved in the body length of cattle breeds[16].
LEMD3	LEM domain containing 3, may be important for the activation of bone lining cells leading to modeling-based bone formation[17].
FBXL15	F-Box and leucine rich repeat protein 15, a key regulator of BMP signaling during embryonic development and adult bone formation[18].
NFKB2	Nuclear factor kappa B subunit 2, is associated with human skeletal size[19].

Supplementary Table 10. Main preys in different saker populations

Population	Main prey	Percent (%) of total food (Mean)	Percent of body fat (%)
Slovakia	*Columba livia domestica*	55.3[20]	3.1-3.8[21]
	Sturnus vulgaris	9.5[20]	4.0-5.6[22]
Mongolia	*Lasiopodomys brandtii*	50.2[23]	7.5-11.7[24]
Qinghai-Tibet Plateau	*Ochotona curzoniae*	> 90[25]	14.6-17.4[26]

Supplementary Table 11. Adaptively introgressed genes related to lipid metabolism

Gene name	Functional description
SCARB1	Scavenger receptor class B type I, mediates selective uptake of high-density lipoprotein cholesterol from blood to liver[27].
MED15	Mediator complex subunit 15, a mediator subunit required for *SPEBP* control of cholesterol and lipid homeostasis[28].
MGAT4C	*MGAT4* family member C, is associated with the APOB (main apolipoprotein of chylomicrons and low-density lipoprotein) level in human[29].
LRP1B	Low-density lipoprotein receptor-related protein 1B, binds to APOE which is a component of lipoproteins[30].

Supplementary Table 12. GO enrichment of the positively selected genes in QTP sakers

GO ID	GO Term	GO Class	Adjusted *P*-value	Gene name
GO:0005344	Oxygen transporter activity	MF	3.62E-06	*HBZ*, *HBAD*, *HBA1*
GO:0019825	Oxygen binding	MF	1.80E-05	*HBZ*, *HBAD*, *HBA1*
GO:0005833	Hemoglobin complex	CC	2.40E-05	*HBZ*, *HBAD*, *HBA1*
GO:0015671	Oxygen transport	BP	4.02E-05	*HBZ*, *HBAD*, *HBA1*
GO:0044464	Cell part	CC	1.17E-03	*GSG1L*, *GTF3C1*, *SNRNP25*, *RHBDF1*, *HBZ*, *HBAD*, *HBA1*, *PGAP6*, *MRPL28*, *AXIN1*, *PDIA2*, *ARHGDIG*, *FAM234A*, *LUC7L*, *CLCN7*, *EPAS1*
GO:0005737	Cytoplasm	CC	1.74E-03	*RHBDF1*, *HBZ*, *HBAD*, *HBA1*, *MRPL28*, *AXIN1*, *PDIA2*, *ARHGDIG*, *EPAS1*
GO:0009968	Negative regulation of signal transduction	BP	2.22E-03	*NPRL3*, *AXIN1*, *RGS11*
GO:0044424	Intracellular part	CC	4.33E-03	*GTF3C1*, *SNRNP25*, *RHBDF1*, *HBZ*, *HBAD*, *HBA1*, *MRPL28*, *AXIN1*, *PDIA2*, *ARHGDIG* *LUC7L*, *CLCN7*, *EPAS1*
GO:0020037	Heme binding	MF	9.66E-03	*HBZ*, *HBAD*, *HBA1*
GO:0009966	Regulation of signal transduction	BP	2.10E-02	*GSG1L*, *NPRL3*, *AXIN1*, *RGS11*
GO:0044444	Cytoplasmic part	CC	2.70E-02	*RHBDF1*, *HBZ*, *HBAD*, *HBA1*, *MRPL28*, *PDIA2*
GO:0030529	Ribonucleoprotein complex	CC	3.58E-02	*SNRNP25*, *MRPL28*, *LUC7L*
GO:0044428	Nuclear part	CC	4.46E-02	*SNRNP25*, *LUC7L*, *CLCN7*, *EPAS1*

Note: MF is the molecular function, CC is the cellular component, and BP is the biological process. A *Chi-square* test was used and *P*-value was adjusted by FDR method.

Supplementary Table 13. Coding variants in the focal sweep under positive selection between QTP and MN saker populations

Chromosome	Position	Gene	*P*-value	Amino acid substitution
Chr 4	47,669,880	*KIAA0556*	0.01	Leu->Ser
Chr 4	47,716,501	*GTF3C1*	0.03	Gly->Ser
Chr 4	47,717,136	*GTF3C1*	0.03	Ser->Thr
Chr 4	47,720,644	*GTF3C1*	0.01	Synonymous
Chr 4	47,755,512	*RHBDF1*	0.03	Cys->Tyr
Chr 4	47,883,633	*HBZ*	0.02	Synonymous
Chr 4	47,887,585	*HBAD*	6.6E-07	Val->Met
Chr 4	47,890,830	*HBA1*	4.8E-04	Val->Met
Chr 4	47,907,389	*PGAP6*	3.0E-03	Synonymous
Chr 4	47,909,129	*PGAP6*	3.0E-03	Val->Ile
Chr 4	47,947,310	*AXIN1*	0.04	Synonymous

Note: The significance level was calculated using a *hapFLK* test.

Supplementary Table 14. Statistics of Iso-Seq data from a QTP saker

Read number (M)	Total base (Gb)	Average read length (Kb)	Mapping rate in the genome (%)	Transcript number in the focal hard sweep	Expressed gene number in the focal hard sweep
14.57	15.49	1.06	27.04	49	11

Reference

1. Glutz von Blotzheim, U. N., Bauer, K. M. & Bezzel, E. Handbuch der Vfgel Mitteleuropas, Band 4. Akademische Verlagsgesellschaft, Frankfurt am Main (1971).
2. Gamauf, A. & Dosedel, R. Satellite telemetry of saker falcons (*Falco cherrug*) in Austria: juvenile dispersal at the westernmost distribution limit of the species. *Aquila* **119**, 65-78 (2012).
3. Dixon, A. *et al*. Variation in electrocution rate and demographic composition of Saker Falcons electrocuted at power lines in Mongolia. *J. Raptor Res.* **54**, 136-146 (2020).
4. Kenward, E. R., Pfeffer, R. H., Al-Bowardi, M. A. & Fox, N. Settting harness sizes and other marking techniques for a falcon with strong sexual dimorphism. *J. Field Ornithol.* **72**, 244-257 (2001).
5. Sackton, T. B. *et al*. Convergent regulatory evolution and loss of flight in paleognathous birds. *Science* **364**, 74-78 (2019).
6. Lee, M., Park, H., Heo, J. M., Choi, H. J., Seo, S. Multi-tissue transcriptomic analysis reveals that L-methionine supplementation maintains the physiological homeostasis of broiler chickens than D-methionine under acute heat stress. *PLoS One* **16**: e0246063 (2021).
7. Sun, H., Liu, P., Nolan, L. K., Lamont, S. J. Avian pathogenic Escherichia coli (APEC) infection alters bone marrow transcriptome in chickens. *BMC Genom*. **16**: 690 (2015).
8. Hong, H. *et al*. Non-coding transcriptome maps across twenty tissues of the Korean black chicken, Yeonsan Ogye. *Int. J. Mol. Sci*. **19**: 2359 (2018).
9. Liang, Y. *et al*. Folding keratin gene clusters during skin regional specification. *Dev. Cell* **53**, 561-576 (2020).
10. Zhan, X. *et al*. Peregrine and saker falcon genome sequences provide insights into evolution of a predatory lifestyle. *Nat. Genet.* **45**, 563-566 (2013).
11. O'Connor, R. E. *et al*. Chromosome-level assembly reveals extensive rearrangement in saker falcon and budgerigar, but not ostrich genomes. *Genome Biol.* **19**, 171 (2018).
12. Pitulescu, M., Kessel, M. & Luo, L. The regulation of embryonic patterning and DNA replication by geminin. *Cell Mol. Life Sci.* **62**, 1425-33 (2005).
13. Sun, Z. *et al*. *FoxO6* regulates Hippo signaling and growth of the craniofacial complex. *PLoS Genet*. **14**, e1007675 (2018).
14. Weedon, M. N. *et al*. A common variant of *HMGA2* is associated with adult and childhood height in the general population. *Nat. Genet.* **39**, 1245-1250 (2007).
15. Federico, A. *et al*. *Hmga1*/*Hmga2* double knock-out mice display a "superpygmy" phenotype. *Biol. Open* **3**, 372-378 (2014).

16. Wu, M. *et al*. Exploring insertions and deletions (indels) of *MSRB3* gene and their association with growth traits in four Chinese indigenous cattle breeds. *Arch. Anim. Breed* **62**, 465-475 (2019).
17. Frost, M. *et al*. Modeling-based bone formation transforms trabeculae to cortical bone in the sclerotic areas in Buschke-Ollendorff syndrome. A case study of two females with *LEMD3* variants. *Bone* **135**, 115313 (2020).
18. Cui, Y. *et al*. SCF^{FBXL15} regulates BMP signalling by directing the degradation of HECT-type ubiquitin ligase Smurf1. *EMBO J.* **30**, 2675-2689 (2011).
19. Huang, Q. Y. *et al*. Genome scan for QTLs underlying bone size variation at 10 refined skeletal sites: genetic heterogeneity and the significance of phenotype refinement. *Physiol. Genomics* **17**, 326-331 (2004).
20. Chavko, J., Obuch, J., Lipták, J., Slobodník, R. & Baláž, M. Changes in nesting habitat of the saker falcon (*Falco cherrug*) influenced its diet composition and potentially threatened its population in Slovakia in the years 1976-2016. *Raptor J.* **13**, 75-10 (2019).
21. Liu, X. *et al*. Comparison of the nutritive composition and histological structure of the meat of hazel grouse (*Tetrastes bonasia amurensis*) and pigeon. *Bulletin of Veterinary College of PLA* **11**, 31-36 (1991).
22. Coleman, J. D. & Robson, A. B. Variations in body weight, fat-free weights and fat deposition of starlings in New Zealand. *Proc. N. Z. Ecol. Soc.* **22**, 7-13 (1975).
23. Gombobaatar, S., Sumiya, D., Shagdarsuren, O., Potapov, E. & Fox, N. Diet studies of Saker Falcon (*Falco cherrug*) in Mongolia. Proceedings of the II International Conference on the Saker Falcon and Houbara Bustard, Mongolia, Ulaanbaatar, Jul. 2000, 116-127 (2001).
24. Liu, X. *et al*. Photoperiod induced obesity in the Brandt's vole (*Lasiopodomys brandtii*): a model of 'healthy obesity'? *Dis. Model. Mech.* **9**, 1357-1366 (2016).
25. Smith, A. T. & Foggin, J. M. The plateau pika (*Ochotona curzoniae*) is a keystone species for biodiversity on the Tibet plateau. *Anim. Conserv.* **2**, 235-240 (1999).
26. Wang, J. Seasonal regulations and influence factors in energy metabolism, thermogenic capacity and body mass in plateau pikas and root voles from the Qinghai-Tibet Plateau. PhD. Thesis, Northwest Institute of Plateau Biology, Chinese Academy of Sciences (2006).
27. Shen, W., Azhar, S., & Kraemer, F. B. SR-B1: A unique multifunctional receptor for cholesterol influx and efflux. *Annu. Rev. Physiol*. **80**, 95-116 (2018).
28. Yang, F. *et al*. An ARC/Mediator subunit required for *SREBP* control of cholesterol and lipid homeostasis. *Nature* **442**, 700-704 (2006).
29. Hamzić, E. *et al*. Genome-wide association study and biological pathway analysis of the Eimeria maxima response in broilers. *Genet. Sel. Evol.* **47**, 91 (2015).

30. Haas, J. *et al*. *LRP1b* shows restricted expression in human tissues and binds to several extracellular ligands, including fibrinogen and apoE-carrying lipoproteins. *Atherosclerosis* **216**, 342-347 (2011).
