# Supplementary material for: Arctic introgression and chromatin regulation facilitated rapid Qinghai-Tibet Plateau colonization by an avian predator
Source: Nat Commun. 2022 Oct 27;13:6413. doi: 10.1038/s41467-022-34138-3 (PMC9613686; doi:10.1038/s41467-022-34138-3)
Supplement: Supplementary file 2 — Description of Additional Supplementary Files [file 41467_2022_34138_MOESM2_ESM.pdf]

Description of Additional Supplementary Files

File Name: Supplementary Data 1
Description: Statistics of the whole genome resequencing data for the falcons.

File Name: Supplementary Data 2
Description: Genes in the adaptively introgressed regions.

File Name: Supplementary Data 3
Description: Positively selected genes in the QTP sakers identified by XP-EHH method.

File Name: Supplementary Data 4
Description: Genes in the focal selective sweep of Chr 4.
