# Supplementary material for: Arctic introgression and chromatin regulation facilitated rapid Qinghai-Tibet Plateau colonization by an avian predator
Source: Nat Commun. 2022 Oct 27;13:6413. doi: 10.1038/s41467-022-34138-3 (PMC9613686; doi:10.1038/s41467-022-34138-3)
Supplement: Supplementary file 7 — Reporting Summary [file 41467_2022_34138_MOESM7_ESM.pdf]

nature research

Corresponding author(s): Xiangjiang Zhan
Last updated by author(s): Oct 9, 2022

Reporting Summary

Nature Research wishes to improve the reproducibility of the work that we publish. This form provides structure for consistency and transparency in reporting. For further information on Nature Research policies, seeAuthors & Referees and theEditorial Policy Checklist .

Statistics

For all statistical analyses, confirm that the following items are present in the figure legend, table legend, main text, or Methods section.

n/a	Confirmed	
☐	☒	The exact sample size (*n*) for each experimental group/condition, given as a discrete number and unit of measurement
☐	☒	A statement on whether measurements were taken from distinct samples or whether the same sample was measured repeatedly
☐	☒	The statistical test(s) used AND whether they are one- or two-sided<br>*Only common tests should be described solely by name; describe more complex techniques in the Methods section.*
☒	☐	A description of all covariates tested
☐	☒	A description of any assumptions or corrections, such as tests of normality and adjustment for multiple comparisons
☐	☒	A full description of the statistical parameters including central tendency (e.g. means) or other basic estimates (e.g. regression coefficient) AND variation (e.g. standard deviation) or associated estimates of uncertainty (e.g. confidence intervals)
☐	☒	For null hypothesis testing, the test statistic (e.g. *F*, *t*, *r*) with confidence intervals, effect sizes, degrees of freedom and *P* value noted<br>*Give P values as exact values whenever suitable.*
☒	☐	For Bayesian analysis, information on the choice of priors and Markov chain Monte Carlo settings
☒	☐	For hierarchical and complex designs, identification of the appropriate level for tests and full reporting of outcomes
☐	☒	Estimates of effect sizes (e.g. Cohen's *d*, Pearson's *r*), indicating how they were calculated

Our web collection on statistics for biologists contains articles on many of the points above.

Software and code

Policy information about availability of computer code

Data collection	Samples for genome sequencing (PacBio, Bionano and HiSeq), ATAC-seq, Hi-C, Iso-seq data were prepared in the lab. The sequencing was outsourced to specialized companies (PacBio and Hi-C at ANOROAD; Bionano at Nextomics Biosciences; HiSeq and Iso-Seq at BGI and Novogene; ATAC-seq at Shanghai Jiayin Biotechnology), and the clean sequencing data were transferred to our lab for downstream analyses.
Data analysis	SMRTlink (v5.0), wtdbg2 (v1.2.8), BWA (v0.7.12), pilon (v1.22), Solve (v3.1), HiC-Pro (v2.7.8) for the genome assembly; RepeatMasker (v 4.0.8), GENEWISE (v2.2.0), InterProScan (v4.7) for the genome annotation; Samtools (v1.9), Genome Analysis Toolkit (v3.3.0), Picard (v1.95) for the SNP calling; SMC++ (v1.9.2), MSMC (v2.1.2), BEAGLE (v4.1), Frappe (v1.1), EIGENSOFT (v3.0), Admixtools (v5.1), Ancestry_HMM (v0.94), fastsimcoal2 (v2.6) for the population genetic analysis; Figtree (v1.4.3) for the phylogeny of W chromosome; MaxEnt26 (v3.3.3k) for the Ecological Niche Modeling analysis; TMHMM (v2.0) for the transmembrane domains of SCARB1 protein analysis; HAPFLK (v1.4), Selscan (v1.2.0), VCFtools (v0.1.13) for the selection analysis; BWA (v0.7.12) and MACS (v2), BEDTools (v2.25.0), LASTZ140 (v1.04.00), deepTools (v3.5.0) for the ATAC-Seq analysis; HiC-pro (v2.7.8) and HiCExplorer (v3) and HICCUPS (v1.0.0) for the Hi-C analysis; Minimap2 (v2.13) and cDNA_Cupcake (v5.8) for the Iso-Seq analysis; Bowtie (v2.3.4.3), RSEM (version 1.3.1), edgeR (v3.32.0) for the RNA-seq analysis; OceanView (v1.6.7), R (v4.0.3) packages for plumage measurements and comparisons.

For manuscripts utilizing custom algorithms or software that are central to the research but not yet described in published literature, software must be made available to editors/reviewers. We strongly encourage code deposition in a community repository (e.g. GitHub). See the Nature Research guidelines for submitting code & software for further information.

Data

Policy information about availability of data

All manuscripts must include a data availability statement. This statement should provide the following information, where applicable:

- Accession codes, unique identifiers, or web links for publicly available datasets
- A list of figures that have associated raw data
- A description of any restrictions on data availability

All sequencing data generated and/or analysed during the study and the genome assembly have been deposited in the CNCB database under accession code PRJCA010321 (https://ngdc.cncb.ac.cn/bioproject/browse/PRJCA010321).

Field-specific reporting

Please select the one below that is the best fit for your research. If you are not sure, read the appropriate sections before making your selection.

☒ Life sciences ☐ Behavioural & social sciences ☐ Ecological, evolutionary & environmental sciences

For a reference copy of the document with all sections, see nature.com/documents/nr-reporting-summary-flat.pdf

Life sciences study design

All studies must disclose on these points even when the disclosure is negative.

Sample size

For the saker reference genome assembly: a blood sample from an adult female saker falcon was collected for the PacBio, HiSeq and Bionano sequencing.
For the genome resequencing: 30 saker samples were collected across Eurasia (2 from Moldova, 5 from Slovakia, 3 from Crimea, 10 from Mongolia and 10 from Qinghai-Tibet Plateau (QTP), China). 10 blood samples from gyrfalcon across Eurasian Arctic (3 from Kola, 3 from Yamal and 4 from Chukotka in Russia. Sampling was conducted across Eurasian in order to cover the main breeding areas of the study populations.
For other analyses: ATAC-seq (3 QTP sakers); Hi-C (2 sakers from Mongolia and 2 from QTP); hemoglobin measurement (8 sakers from Mongolia and 6 from QTP); Iso-Seq (1 saker from QTP); high density lipoprotein cholesterol (HDLC) measurements (5 sakers from Slolvakia, 6 from Mongolia and 6 from QTP).
Morphological analysis on sakers: plumage color (11 from Mongolia and 11 QTP) and body mass measurements (19 from QTP).
For the population genomics analysis: sample size larger than 5 individuals in each study location was used.
For ATAC-seq and Hi-C: sample size of 2-3 individuals in each study population was used for downstream analysis.
For Iso-Seq: one sample was used to identify the full length transcript.
For HDLC, hemoglobin and plumage color measurements, sample size larger than 5 individuals in each study population was used for significance test.

Data exclusions

None.

Replication

We performed the luciferase reporter experiments (n = 6) and in vitro over expression experiments (n = 3) with multiple repeats in each batch and three independent batches in total to confirm reproducibility. All attempts at replication were successful. The number of replications is noted in Methods.

Randomization

We used the duck embryo fibroblast cells (ATCC CCL-141) and human HeLa cells (ATCC CCL-2) ordered from ATCC for experiments. The cells were selected randomly for cell culture. Experimental groups were randomly allocated for the following lipofection, luciferase reporter assay and in vitro over expression experiments.

Blinding

Data included in this study were generated by different teams, and analyzed by different coauthors.

Reporting for specific materials, systems and methods

We require information from authors about some types of materials, experimental systems and methods used in many studies. Here, indicate whether each material, system or method listed is relevant to your study. If you are not sure if a list item applies to your research, read the appropriate section before selecting a response.

Materials & experimental systems

n/a	Involved in the study	
☐	☒	Antibodies
☐	☒	Eukaryotic cell lines
☒	☐	Palaeontology
☐	☒	Animals and other organisms
☒	☐	Human research participants
☒	☐	Clinical data

Methods

n/a	Involved in the study	
☒	☐	ChIP-seq
☒	☐	Flow cytometry
☒	☐	MRI-based neuroimaging

Antibodies

Antibodies used	The Anti-GFP antibody (Abcam, Cat# ab183734, Lot# GR254056-32) was used to detect the expression of GFP fusion protein, β-Actin monoclonal antibody (Gene-Protein Link, Cat# P01L03, Lot# GP002L) was used to detect the expression of endogenous β-Actin protein, Goat anti-rabbit IgG-HRP (Gene-Protein Link, Cat# P03S02S, Lot# 04001) and Horse anti-mouse IgG-HRP (Cell Signaling Technology, Cat# 7076, Lot# 34) were used as the secondary antibodies. Anti-GFP antibody (EPR14104); β-Actin monoclonal antibody (5B7).
Validation	The Anti-GFP antibody (Abcam, ab183734) was ordered from Abcam and β-Actin monoclonal antibody from Gene-Protein Link. All antibodies were used based on the respective statements for species (rabbit or mouse). All antibodies were validated by the manufacturers. The Anti-GFP antibody was also validated in the Hao et al., Nature communications 2019. Anti-GFP antibody (EPR14104) is suitable for WB, IHC-P, ICC/IF and Flow Cyt. β-Actin monoclonal antibody (5B7) is suitable for IF, WB and IHC-P.

Eukaryotic cell lines

Policy information about cell lines

Cell line source(s)	The duck embryo fibroblast cells (ATCC CCL-141) and human HeLa cells (ATCC CCL-2) were ordered from ATCC.
Authentication	The duck embryo fibroblast cells were authenticated using morphology (fibroblast) and species determination (COI assay) by the manufacturer. The HeLa cells were authenticated using morphology (epithelial) and STR method by the manufacturer.
Mycoplasma contamination	NA.
Commonly misidentified lines (See ICLAC register)	NA.

Animals and other organisms

Policy information about studies involving animals; ARRIVE guidelines recommended for reporting animal research

Laboratory animals	This study does not involve laboratory animals.
Wild animals	Nestling falcons aged ca. 4-7 weeks old were temporarily removed from the nest and were worn caps in order to take samples/measure plumage, and were replaced after the procedure, which took no more than 30 minutes. No case of adverse reaction, injury, nest desertion or mortality was recorded due to this procedure.
Field-collected samples	Blood samples were taken from nestling falcons in the field via brachial venipuncture. The brachial vein area was swabbed with 75% ethanol and the needle of a syringe aligned with the longitudinal axis of the vein towards body. The vein was penetrated with the needle at a shallow angle and up to 0.2 ml of blood was collected. On withdrawing the needle, a cotton wool swab was gently compressed over the puncture wound until bleeding ceased. Blood was then evacuated from the syringe into storage tubes (4 ml BD Plastic Whole Blood tube with spray-coated K2EDTA 7.2 mg for DNA; QIAGEN RNA protect Animal Blood Tubes for RNA). 2-3 Plucked chick feathers were collected from chest and stored in 75% ethanol.
Ethics oversight	All lab experiment procedures were under the guidance of the Ethics Committee of the Institute of Zoology, Chinese Academy of Sciences (IoZ, CAS). The collection and processing of falcon tissues in this study were conducted in accordance with the guidelines of Institutional Animal Care and Use Committee of the Institute of Zoology, Chinese Academy of Sciences.

Note that full information on the approval of the study protocol must also be provided in the manuscript.
